# Effects of spatial heterogeneity on bacterial genetic circuits

**DOI:** 10.1371/journal.pcbi.1008159

**Published:** 2020-09-14

**Authors:** Carlos Barajas, Domitilla Del Vecchio

**Affiliations:** Department of Mechanical Engineering, Massachusetts Institute of Technology, Cambridge, MA 02139-4307, USA; Rutgers University, UNITED STATES

## Abstract

Intracellular spatial heterogeneity is frequently observed in bacteria, where the chromosome occupies part of the cell’s volume and a circuit’s DNA often localizes within the cell. How this heterogeneity affects core processes and genetic circuits is still poorly understood. In fact, commonly used ordinary differential equation (ODE) models of genetic circuits assume a well-mixed ensemble of molecules and, as such, do not capture spatial aspects. Reaction-diffusion partial differential equation (PDE) models have been only occasionally used since they are difficult to integrate and do not provide mechanistic understanding of the effects of spatial heterogeneity. In this paper, we derive a reduced ODE model that captures spatial effects, yet has the same dimension as commonly used well-mixed models. In particular, the only difference with respect to a well-mixed ODE model is that the association rate constant of binding reactions is multiplied by a coefficient, which we refer to as the binding correction factor (BCF). The BCF depends on the size of interacting molecules and on their location when fixed in space and it is equal to unity in a well-mixed ODE model. The BCF can be used to investigate how spatial heterogeneity affects the behavior of core processes and genetic circuits. Specifically, our reduced model indicates that transcription and its regulation are more effective for genes located at the cell poles than for genes located on the chromosome. The extent of these effects depends on the value of the BCF, which we found to be close to unity. For translation, the value of the BCF is always greater than unity, it increases with mRNA size, and, with biologically relevant parameters, is substantially larger than unity. Our model has broad validity, has the same dimension as a well-mixed model, yet it incorporates spatial heterogeneity. This simple-to-use model can be used to both analyze and design genetic circuits while accounting for spatial intracellular effects.

## Author summary

A general and simple modeling framework to determine how spatial heterogeneity modulates the dynamics of gene networks is currently lacking. To this end, we provide a simple-to-use ordinary differential equation (ODE) model that can be used to both analyze and design genetic circuits while accounting for spatial intracellular effects. We apply our model to several core biological processes and determine that transcription and its regulation are more effective for genes located at the cell poles than for genes located on the chromosome and this difference increases with regulator size. For translation, we predict the effective binding between ribosomes and mRNA is higher than that predicted by a well-mixed model, and it increases with mRNA size. We provide examples where spatial effects are significant and should be considered but also where a traditional well-mixed model suffices despite severe spatial heterogeneity. Finally, we illustrate how the operation of well-known genetic circuits is impacted by spatial effects.

## Introduction

Deterministic models of gene circuits typically assume a well-mixed ensemble of species inside the cell [1, 2]. This assumption allows one to describe genetic circuit dynamics through a set of ODEs, for which a number of established analysis tools are available [1]. However, it is well known that spatial heterogeneity is prevalent inside bacterial cells [3–8]. Depending on the origin of replication, plasmids tend to localize within bacterial cells [9–11]. Furthermore, chromosome genes (endogenous and synthetically integrated ones [12]) are distributed in the cell according to the chromosome complex spatial structure. In bacterial cells, any molecule freely diffusing through the chromosome (e.g., mRNA, ribosome, and protease) experiences what are known as *excluded volume effects*, which capture the tendency of species to be ejected from the nucleoid due to the space occupied by the dense DNA mesh [13]. These excluded volume effects for ribosomes and RNAP in bacteria have been observed experimentally [14].

Despite the strong evidence in support of spatial heterogeneity within bacterial cells, a convenient modeling framework that captures the spatio-temporal organization of molecules inside the cell is largely lacking. As a consequence, how spatial effects modulate genetic circuit dynamics remains also poorly understood. Partial differential equation (PDE) models have been employed on an *ad hoc* basis to numerically capture intracellular spatial dynamics for specific case studies [15–17]. Although a general PDE model of a gene regulatory network (GRN) can be constructed, it is difficult to analyze and impractical for design [18]. Recently, the method of matched asymptotic expansions was used to simplify the PDEs to a set of ODEs to analyze ribosome-mRNA interactions [19]. Similarly, [20] used a compartmentalized model to capture spatial heterogeneity in sRNA-mRNA interactions. However, these results have not been generalized, relied on simulation, and specific parameter values.

In this paper, we provide a general framework to model spatial heterogeneity through an ODE that has the same structure and hence dimensionality as a well-mixed ODE model. To this end, we first introduce a PDE model that captures spatial dynamics. Next, we exploit the time scale separation between molecule diffusion and biochemical reactions to derive a reduced order ODE model of the space averaged dynamics. This model accounts for spatial heterogeneity by multiplying the association rate constant of binding reactions by a factor that depends on the size of freely diffusing species and on the location of spatially fixed species. We call this factor the *binding correction factor* (BCF). Thus, this reduced model has the same dimensionality as traditional well-mixed models, yet it captures spatial effects.

We demonstrate the effects of spatial heterogeneity in genetic circuit behavior by modeling and analyzing several core biological processes. We show that the transcription rate of a gene and the affinity at which transcription factors bind to it, is lower (higher) when the gene is located near mid-cell (cell poles) with respect to the well-mixed model. We show that compared to a well-mixed model, translation rate is always higher and increases with mRNA size. Finally, we consider a genetic clock, a circuit that produces sustained oscillations. We show that for a parameter range where a well-mixed model predicts sustained oscillations, a model that accounts for spatial heterogeneity of DNA may not show oscillations. All of these phenomena can be recapitulated by our reduced ODE model.

## Materials and methods

We use mathematical models to investigate the effects of spatial heterogeneity, specifically DNA localization and excluded volume effects, on genetic circuit behavior. The first part of this section introduces the mathematical model used, a set of nonlinear PDEs. Model reduction is performed on the resulting PDEs to obtain the reduced ODE model that we use to predict how molecule size and location affect genetic circuit’s behavior. The numerical method used to simulate the PDEs in this study is discussed in S1 Text: Section 6.

### Reaction-diffusion model

A reaction-diffusion model describes the concentration of a species at a given time and location in the cell. We focus on enzymatic-like reactions since they can be used to capture most core processes in the cell. We specialize the model to the cases where the reacting species both freely diffuse or where one freely diffuses while the other one is fixed. For example, mRNA and ribosomes are both freely diffusing, while for RNA polymerase and DNA, one is freely diffusing and the other one is fixed.

#### Enzymatic-like reactions that model core biological processes

Let S be a substrate being shared by *n* enzymes E_i_, to form product P_i_ where *i* = 1,…, *n*. The rate at which E_i_ and S are produced is given by *α*_*i*_ and *α*_*s*_, respectively. The decay rates of E_i_ and S are given by *γ*_*i*_ and *γ*_*s*_, respectively. Here, we assume that E_i_ and S can be degraded even in complex form, that is, the complex is not protecting them from degradation. Finally, all species are diluted as the cell divides at a rate *μ*. The biochemical reactions corresponding to this process are given by:

$$\begin{array}{c} \emptyset \overset{\alpha_{i}}{⟶}E_{i}\overset{\gamma_{i}+\mu }{⟶}\emptyset ,\;\emptyset \overset{\alpha_{s}}{⟶}S\overset{\gamma_{s}+\mu }{⟶}\emptyset ,\;c_{i}\overset{\gamma_{i}}{⟶}S,\;c_{i}\overset{\gamma_{s}}{⟶}E_{i},\\ c_{i}\overset{\mu }{⟶}\emptyset ,\;E_{i}+S\overset{a_{i}}{\underset{d_{i}}{⇌}}c_{i}\overset{\kappa_{i}}{⟶}P_{i}+E_{i}+S, \end{array}$$ (1)

where c_i_ is the complex formed when E_i_ binds to S, *a*_*i*_ is the association rate constant, *d*_*i*_ is the dissociation rate constant, and *κ*_*i*_ is the catalytic rate constant of product formation. These enzymatic-like reactions capture many core biological processes such as genes transcribed by RNA polymerase, mRNA translated by ribosomes, or proteins degraded by a common protease [1]. Notice that they differ from the classical enzymatic reactions since the substrate is not converted into product [1].

**Fig 1:**
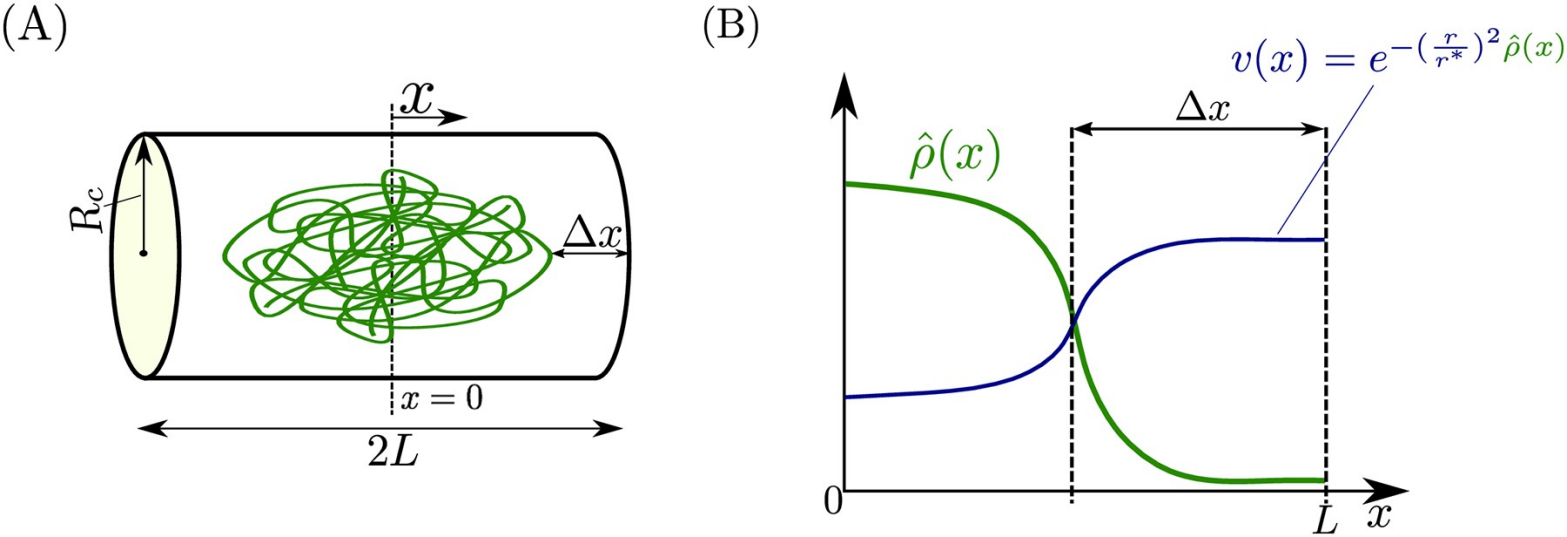
Intracellular spatial geometry. (A) We model the cell as a cylinder of radius *R*_*c*_ and length 2*L*. The distance between the end of the chromosome and the cell poles is Δ*x*. (B) The spatial profiles for the normalized local density of DNA length $\hat{\rho }\left(x\right)$ and the fraction of available volume *v*(*x*) of a freely diffusing species with radius of gyration *r* within the chromosomal mesh. These two quantities are related by $v\left(x\right)=e^{-{\left(r/r^{*}\right)}^{2}\hat{\rho }\left(x\right)}$, where r* is a length scale dependent on the averaged chromosome density in the cell given by (2). The chromosome density is assumed to be monotonically decreasing from mid-cell to the cell poles (as in [13]), thus the available volume profile are monotonically increasing.

*E. coli* actively regulates its geometry to achieve a near-perfect cylindrical shape [21]. Thus, we model the cell as a cylinder of length 2*L* and radius *R*_*c*_. This geometry is shown in Fig 1A. We assume angular and radial homogeneity ((*R*_*c*_/*L*)^2^ ≪ 1) such that the concentration of a species varies only axially (the spatial *x* direction). Symmetry relative to mid-cell is assumed and hence only half of the cell is considered, that is, *x* ∈ [0, *L*], where *x* = 0 is at mid-cell and *x* = *L* is at the cell poles. Furthermore, we assume a constant cross-sectional area along the axial direction.

In [14] it was shown that polysomes were excluded from the dense chromosomal DNA mesh onto the cell poles. These phenomena is generalized for any species that freely diffuses within the DNA mesh and is referred to as “excluded volume effects”. Leveraging the diffusion modeling framework from [13], we now specify the model to capture excluded volume effects. Let *v*(*x*) ∈ (0, 1] be the volume fraction (dimensionless) available to a species to diffuse within the chromosome (Fig 1B). As derived in [13] and discussed in S1 Text: Section 2, the available volume profile *v*(*x*) of a species with a radius of gyration *r*, takes the form

$$\begin{array}{c} v\left(x\right)=e^{-{\left(r/r^{*}\right)}^{2}\hat{\rho }\left(x\right)},\;{\left(r^{*}\right)}^{2}=\frac{V_{p}}{2\kappa \pi L_{p}}, \end{array}$$ (2)

where $\hat{\rho }\left(x\right)$ is the normalized local density of chromosome DNA length such that $\frac{2}{L}\int_{0}^{L}\hat{\rho }\left(x\right)dx=1$, *L*_*p*_ is the total length of chromosome DNA, *V*_*p*_ the volume where the DNA polymer is confined, such that *L*_*p*_/*V*_*p*_ is the total DNA length per volume, and *κ* is an empirically determined correction factor (see [13] and S1 Text: Section 2). The quantity (r*)^2^ is inversely proportional to the total DNA length per volume. For all simulations in the main text of this study, we model the normalized chromosome density as

$$\begin{array}{c} \hat{\rho }\left(x\right)=\frac{1}{1+e^{20(x/L-1/2)}}, \end{array}$$

as experimentally determined in [13]. This model for $\hat{\rho }\left(x\right)$ is monotonically decreasing (i.e, the chromosome is more dense near mid-cell than at the cell poles as shown in Fig 1B). Therefore by (2), the available volume profile is higher near mid-cell than at the cell poles (i.e., *v*(0) < *v*(*L*)) as shown in Fig 1B and furthermore, the discrepancy between *v*(0) and *v*(*L*) increases with *r*/r*. We note that the specific expressions of $\hat{\rho }\left(x\right)$ and *r*/r* do not affect the model reduction result of this paper. The main results in this paper are presented for a constant cell length *L* and chromosome DNA density $\hat{\rho }\left(x\right)$, however in S1 Text: Section 9 we relax these assumptions and allow these quantities to vary in time as the cell divides.

For any given species with concentration per unit length given by *y*(*t*, *x*), free to diffuse, with available volume *v*(*x*), an expression for the flux term, derived in [13] is given by:

$$\begin{array}{c} J\left(x,y\right)=D(\underset{\text{towards}\;\text{low}\;\text{concentration}}{\underset{︸}{-\frac{\partial y(t,x)}{\partial x}v\left(x\right)}}+\underset{\text{towards}\;\text{high}\;\text{available}\;\text{volume}}{\underset{︸}{y\left(t,x\right)\frac{\partial v(x)}{\partial x}}})=-v^{2}\left(x\right)\frac{d}{dx}[\frac{y(t,x)}{v(x)}], \end{array}$$ (3)

where *D* is the diffusion coefficient. The flux is driven by two mechanisms: the first is concentration gradient, which pushes molecules from high to low concentrations and the second drives molecules to regions with a higher volume fraction. This second term is referred to as the excluded volume effect [13]. From (3), if $\left|\frac{\partial y(t,x)}{\partial x}v\left(x\right)|<|y\left(t,x\right)\frac{\partial v(x)}{\partial x}\right|$ and $\frac{\partial y(t,x)}{\partial x}\frac{\partial v(x)}{\partial x}>0$, then the net flux is from low to high concentration, which is the case when species are repelled from the chromosome to high concentration areas in the cell poles. As we will show, this mechanism dictates intracellular heterogeneity in the limit of fast diffusion.

For species S, we denote by *S*(*t*, *x*) its concentration per unit length at time *t* at location *x* (similarly for E_i_ and c_i_). Assuming sufficently high molecular counts, the reaction-diffusion dynamics corresponding to (1) describing the rate of change of the species concentrations at position *x*, are given by [22]:

$$\begin{array}{cc} \frac{\partial E_{i}\left(t,x\right)}{\partial t}= & -\frac{d}{dx}[J(x,E_{i})]-a_{i}E_{i}\left(t,x\right)S\left(t,x\right)+\left(\gamma_{s}+d_{i}+\kappa_{i}\right)c_{i}\left(t,x\right)\\ & +\alpha_{i}\left(t,x\right)-\left(\gamma_{i}+\mu \right)E_{i}\left(t,x\right),\\ \frac{\partial c_{i}\left(t,x\right)}{\partial t}= & -\frac{d}{dx}[J(x,c_{i})]+a_{i}E_{i}\left(t,x\right)S\left(t,x\right)-\left(\gamma_{i}+\gamma_{s}+d_{i}+\kappa_{i}+\mu \right)c_{i}\left(t,x\right),\\ \frac{\partial S(t,x)}{\partial t}= & -\frac{d}{dx}[J(x,S)]+\sum_{j=1}^{n}[-a_{j}E_{j}\left(t,x\right)S\left(t,x\right)+\left(\gamma_{j}+d_{j}+\kappa_{j}\right)c_{j}\left(t,x\right)]\\ & +\alpha_{s}\left(t,x\right)-\left(\gamma_{s}+\mu \right)S\left(t,x\right), \end{array}$$ (4)

where *J*(*x*, ⋅) is the flux per unit area per unit time, within the cell. If the species is freely diffusing *J*(*x*, ⋅) is given by (3), otherwise if the species is spatially fixed, then *J*(*x*, ⋅) = 0 for all *x* ∈ [0, *L*]. The boundary conditions associated with freely diffusing species of (4) are zero flux at the cell poles and cell center due to the assumed left-right symmetry, which corresponds to:

$$\begin{array}{c} J(0,\cdot )=J(L,\cdot )=0. \end{array}$$ (5)

Notice that none of the parameters in (4) appearing in (1) depend explicitly on time and space except for the production terms *α*_*i*_(*t*, *x*) and *α*_*s*_(*t*, *x*). The explicit time dependence of the production terms allows us to capture how genes can be activated or repressed externally with a time varying signal [23]. The explicit dependence of the production terms on *x* allows us to capture where the species is produced within the cell (e.g., DNA in the chromosome or DNA in pole localized plasmid genes).

**Dimensionless model**: Depending on the parameter regimes, the dynamics of (4) can display time scale separation. For example, diffusion occurs in the order of mili-seconds compared to minutes for dilution due to cell-growth and mRNA degradation [2]. Therefore, we are interested in determining the behavior of (4) in the limit of fast diffusion. We thus rewrite (4) in dimensionless form to make time scale separation explicit. We nondimensionalize the system variables using dilution (1/*μ*) as the characteristic time scale, the length of the cell (*L*) as the characteristic length, and *μ*/*a*_1_ as the characteristic concentration per length scale: $t^{*}=t\mu ,\;y^{*}=y\frac{a_{1}}{\mu },\;x^{*}=\frac{x}{L},$ where *y* denotes concentration per unit length and the superscript “*” is used on the dimensionless variable. Concentrations are nondimensionalized through *a*_1_ because this parameter contains a concentration scale, it is fixed in time, and it is assumed to be nonzero. The dimensionless form of (4) is given by

$$\begin{array}{cc} \frac{\partial E_{i}^{*}\left(t^{*},x^{*}\right)}{\partial t^{*}} & =-\frac{d}{dx^{*}}[J^{*}\left(x^{*},E_{i}^{*}\right)]+\frac{1}{\eta_{i}}[-E_{i}^{*}\left(t^{*},x^{*}\right)S^{*}\left(t^{*},x^{*}\right)\frac{a_{i}^{*}}{{\tilde{d}}_{i}}+c_{i}^{*}\left(t^{*},x^{*}\right)]\\ & \;+\alpha_{i}^{*}\left(t^{*},x^{*}\right)-\left(\gamma_{i}^{*}+1\right)\left(E_{i}^{*}\left(t^{*},x^{*}\right)+c_{i}^{*}\left(t^{*},x^{*}\right)\right),\\ \frac{\partial c_{i}^{*}\left(t^{*},x^{*}\right)}{\partial t^{*}} & =-\frac{d}{dx^{*}}[J^{*}\left(x^{*},c_{i}^{*}\right)]+\frac{1}{\eta_{i}}[E_{i}^{*}\left(t^{*},x^{*}\right)S^{*}\left(t^{*},x^{*}\right)\frac{a_{i}^{*}}{{\tilde{d}}_{i}}-c_{i}^{*}\left(t^{*},x^{*}\right)],\\ \frac{\partial S^{*}\left(t^{*},x^{*}\right)}{\partial t^{*}} & =-\frac{d}{dx^{*}}[J^{*}\left(x^{*},S^{*}\right)]+\sum_{j=1}^{n}\frac{1}{\eta_{j}}[-E_{j}^{*}\left(t^{*},x^{*}\right)S^{*}\left(t^{*},x^{*}\right)\frac{a_{j}^{*}}{{\tilde{d}}_{j}}+c_{j}^{*}\left(t^{*},x^{*}\right)]\\ & \;+\alpha_{s}^{*}\left(t^{*},x^{*}\right)-\left(\gamma_{s}^{*}+1\right)\left(S^{*}\left(t^{*},x^{*}\right)+\sum_{j=1}^{n}c_{j}^{*}\left(t^{*},x^{*}\right)\right), \end{array}$$ (6)

where $a_{i}^{*}=a_{i}/a_{1}$, $\gamma_{s}^{*}=\gamma_{s}/\mu$, $\gamma_{i}^{*}=\gamma_{i}/\mu$, $d_{i}^{*}=d_{i}/\mu$, $\kappa_{i}^{*}=\kappa_{i}/\mu$, $\alpha_{i}^{*}=\alpha_{i}a_{1}/\mu^{2}$, $\alpha_{s}^{*}=\alpha_{s}a_{1}/\mu^{2}$, ${\tilde{d}}_{i}=\gamma_{i}^{*}+\gamma_{s}^{*}+d_{i}^{*}+\kappa_{i}^{*}+1$, $\eta_{i}=1/{\tilde{d}}_{i}$, and J* = *Ja*_1_/(*μ*^2^ *L*). For a freely diffusing species with diffusion coefficient *D*, the dimensionless parameter that determines the relative speed of diffusion is denoted by *ϵ* = *μL*^2^/*D* and fast diffusion corresponds to *ϵ* ≪ 1. Likewise, *η*_*i*_ in (4) determines the relative speed of the binding dynamics, where *η*_*i*_ ≪ 1 implies these reactions are fast. From hereon, unless otherwise specified, we work with variables in their dimensionless form and drop the star superscript for simplifying notation.

**Space averaged concentrations**: Concentrations per cell are usually the quantities measured experimentally [24] and are the primary quantities of interest. We now derive the space averaged dynamics corresponding to (6), which describe the dynamics of concentrations per half of the cell. We define ${\bar{E}}_{i}\left(t\right)$, $\bar{S}\left(t\right)$, and ${\bar{c}}_{i}\left(t\right)$ to be the *space averaged enzyme, substrate, and complex concentrations*, respectively, and are given by

$$\begin{array}{c} {\bar{E}}_{i}\left(t\right)=\int_{0}^{1}E_{i}\left(t,x\right)dx,\;{\bar{c}}_{i}\left(t\right)=\int_{0}^{1}c_{i}\left(t,x\right)dx,\;\bar{S}\left(t\right)=\int_{0}^{1}S\left(t,x\right)dx, \end{array}$$

also giving the concentrations per half of the cell. The dynamics governing these space averaged variables are derived by integrating (6) in space and applying the boundary conditions (5) and are given by:

$$\begin{array}{cc} & \frac{d{\bar{E}}_{i}\left(t\right)}{dt}={\bar{\alpha }}_{i}\left(t\right)-\frac{1}{\eta_{i}}[{\bar{E}}_{i}\left(t\right)\bar{S}\left(t\right)\frac{a_{i}\theta_{i}\left(t\right)}{{\tilde{d}}_{i}}-{\bar{c}}_{i}\left(t\right)]-\left(\gamma_{i}+1\right)\left({\bar{E}}_{i}\left(t\right)+{\bar{c}}_{i}\left(t\right)\right),\\ & \frac{d{\bar{c}}_{i}\left(t\right)}{dt}=\frac{1}{\eta_{i}}[{\bar{E}}_{i}\left(t\right)\bar{S}\left(t\right)\frac{a_{i}\theta_{i}\left(t\right)}{{\tilde{d}}_{i}}-{\bar{c}}_{i}\left(t\right)],\\ & \frac{d\bar{S}\left(t\right)}{dt}={\bar{\alpha }}_{s}\left(t\right)-\sum_{j=1}^{n}\frac{1}{\eta_{j}}[{\bar{E}}_{j}\left(t\right)\bar{S}\left(t\right)\frac{a_{j}\theta_{j}\left(t\right)}{{\tilde{d}}_{j}}-{\bar{c}}_{j}\left(t\right)]-\left(\gamma_{s}+1\right)\left(\bar{S}\left(t\right)+\sum_{j=1}^{n}{\bar{c}}_{j}\left(t\right)\right), \end{array}$$ (7)

where overbars denote spatially averaged variables and

$$\begin{array}{c} \theta_{i}\left(t\right)=\frac{\int_{0}^{1}E_{i}\left(t,x\right)S\left(t,x\right)dx}{\left[\int_{0}^{1}E_{i}\left(t,x\right)dx][\int_{0}^{1}S\left(t,x\right)dx\right]}. \end{array}$$ (8)

**Fig 2:**
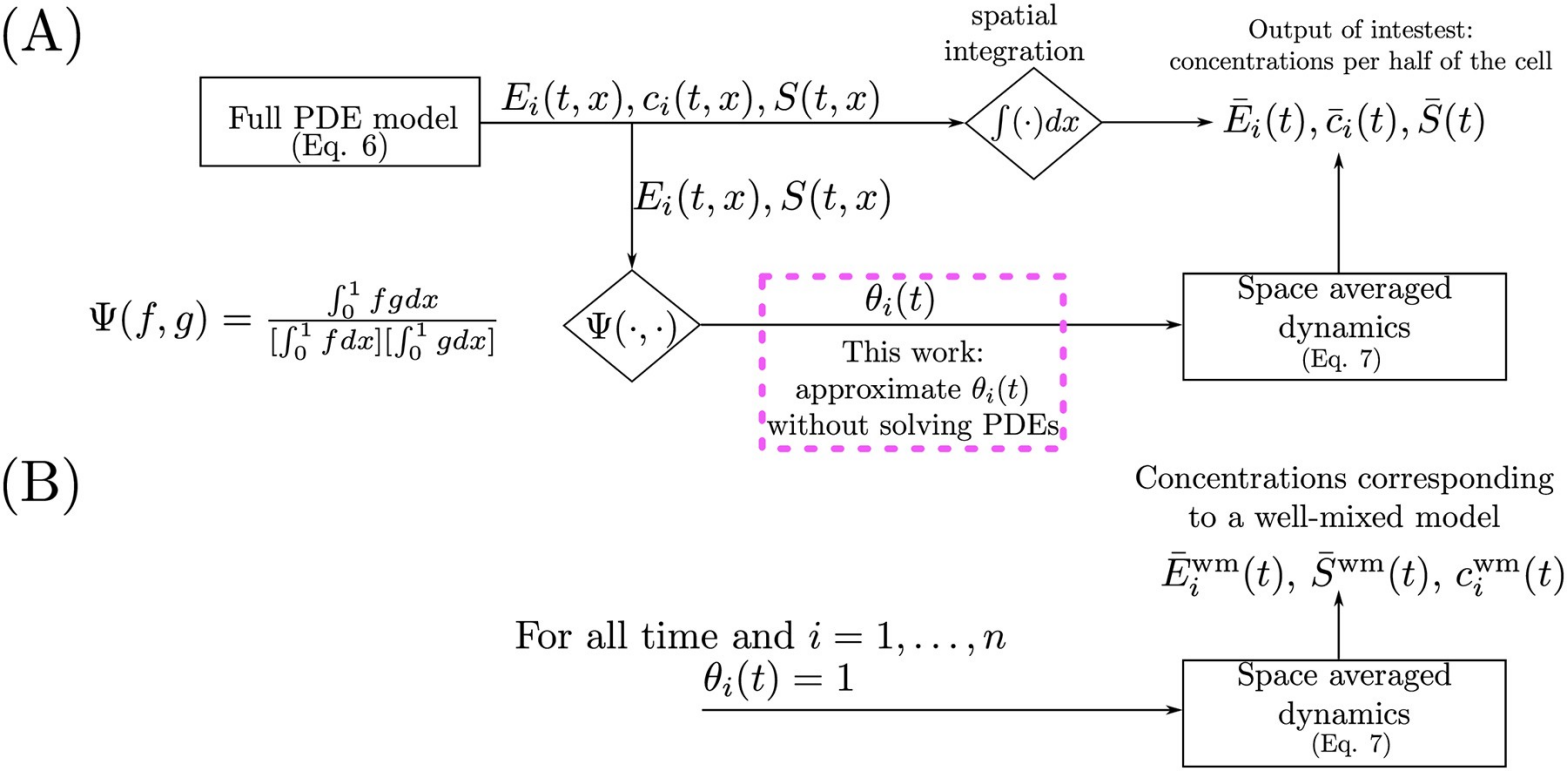
Methods to calculate space averaged concentrations. (A) The time and space dependent solutions of the full PDE (6) are integrated spatially to yield concentrations per half of the cell. Alternatively, space averaged concentrations can be calculated using the space average dynamics (7) with the BCF (*θ*_*i*_(*t*) (8)) as a time varying parameter. (B) The dynamics of the space averaged concentration is given by the well mixed model (9) when *θ*_*i*_(*t*) = 1 for all time and for all *i* = 1, …, *n*.

Therefore, to calculate the space averaged concentrations, one could integrate the outputs of the full PDE (6) directly or use (7) along with (8), as illustrated in Fig 2. Notice that calculating *θ*_*i*_ in (8) requires solving the full PDE system (6) because of its dependence on the product *E*_*i*_(*t*, *x*)*S*(*t*, *x*). Therefore, in general, there is no obvious benefit in working with (7). In this paper, we provide a method to compute a guaranteed approximation of *θ*_*i*_ without solving the PDEs (6).

**Well-mixed model**: Next, we define what we have been informally referring to as the “well-mixed” model [25]. A standard well-mixed model is derived starting from (1) assuming mass action kinetics, that molecular counts are sufficiently large, and that the intracellular environment is spatially homogeneous (well-mixed) [1]. We let ${\bar{E}}_{i}^{\text{wm}}\left(t\right)$, ${\bar{S}}^{\text{wm}}\left(t\right)$ and ${\bar{c}}_{i}^{\text{wm}}\left(t\right)$, denote the well-mixed concentrations of E_i_, S, and c_i_, respectively, and their dynamics are given by

$$\begin{array}{cc} & \frac{d{\bar{E}}_{i}^{\text{wm}}\left(t\right)}{dt}={\bar{\alpha }}_{i}\left(t\right)-\frac{1}{\eta_{i}}[{\bar{E}}_{i}^{\text{wm}}\left(t\right){\bar{S}}^{\text{wm}}\left(t\right)\frac{a_{i}}{{\tilde{d}}_{i}}-{\bar{c}}_{i}^{\text{wm}}\left(t\right)]-\left(\gamma_{i}+1\right)\left({\bar{E}}_{i}^{\text{wm}}\left(t\right)+{\bar{c}}_{i}^{\text{wm}}\left(t\right)\right),\\ & \frac{d{\bar{c}}_{i}^{\text{wm}}\left(t\right)}{dt}=\frac{1}{\eta_{i}}[{\bar{E}}_{i}^{\text{wm}}\left(t\right){\bar{S}}^{\text{wm}}\left(t\right)\frac{a_{i}}{{\tilde{d}}_{i}}-{\bar{c}}_{i}^{\text{wm}}\left(t\right)],\\ & \frac{d{\bar{S}}^{\text{wm}}\left(t\right)}{dt}={\bar{\alpha }}_{s}\left(t\right)-\sum_{j=1}^{n}\frac{1}{\eta_{j}}[{\bar{E}}_{j}^{\text{wm}}\left(t\right){\bar{S}}^{\text{wm}}\left(t\right)\frac{a_{j}}{{\tilde{d}}_{j}}-{\bar{c}}_{j}^{\text{wm}}\left(t\right)]-\left(\gamma_{s}+1\right)\left({\bar{S}}^{\text{wm}}\left(t\right)+\sum_{j=1}^{n}{\bar{c}}_{j}^{\text{wm}}\left(t\right)\right). \end{array}$$ (9)

Comparing (7) and (9), motivates us to define $a_{i}^{\prime }\left(t\right)=a_{i}\theta_{i}\left(t\right)$, which can be regarded as the effective association rate constant between E_i_ and S in (7). We refer to *θ*_*i*_(*t*) as the *binding correction factor* (BCF). The dynamics of the space averaged concentrations (7) coincide with those of the well-mixed model (9) when *θ*_*i*_(*t*) = 1 (thus $a_{i}^{\prime }\left(t\right)=a_{i}$) for all time and for all *i* = 1, …, *n*. From (8) notice that *E*_*i*_(*t*, *x*) and *S*(*t*, *x*) being spatially constant for all time is not necessary for *θ*_*i*_(*t*) = 1 for all time. For example, if *S*(*t*, *x*) is spatially constant while *E*_*i*_(*t*, *x*) has an arbitrary spatial profile (or *vice-versa*), then *θ*_*i*_(*t*) = 1. Thus, the space averaged concentrations can coincide with those of a well-mixed model despite severe spatial heterogeneity. In this work, we provide a constant approximation of *θ*_*i*_(*t*) denoted by $\theta_{i}^{*}$, which depends on spatial variables such as molecule size and gene location. Under the fast diffusion approximation, we show that $\theta_{i}^{*}$ is close to *θ*_*i*_(*t*). The space averaged dynamics (7) with *θ*_*i*_(*t*) replaced by $\theta_{i}^{*}$, thus provides a reduced ODE model that captures spatial information without having to solve (6). We will compare how solutions to (7) with (8) calculated from the full PDE (6) or with $\theta_{i}^{*}$ compare to each other and to the solutions of the well-mixed model (9).

#### Three diffusion cases to capture core biological processes

To use model (6) to describe key biological processes, we consider three cases. In Case I, E_i_ for all *i* = 1, …, *n* and S are all freely diffusing within the cell. In Case II, E_i_ is spatially fixed for all *i* = 1, …, *n* (*J*(*x*, *E*_*i*_) = 0 for all *x* ∈ [0, 1] and for all *i* = 1, …, *n*) and S is freely diffusing. In Case III, E_i_ is freely diffusing for all *i* = 1, …, *n* and S is spatially fixed (*J*(*x*, *S*) = 0 for all *x* ∈ [0, 1]). Case I may represent mRNA molecules (E_i_) competing for ribosomes (S), all freely diffusing in the cell. Case II captures genes (E_i_), which are spatially fixed and are transcribed by RNA polymerase (S), which freely diffuses. Case III models transcription factors (E_i_), which freely diffuse regulating the same spatially fixed gene (S).

The flux dynamics, the boundary conditions, and a core biological process example for each case are summarized in Table 1. When a species is spatially fixed, the flux is zero through the whole domain, that is, *J*(*x*, ⋅) = 0 for all *x* ∈ [0, 1]. The available volume profiles for the enzyme, complex, and substrate are denoted by $v_{E_{i}}\left(x\right)$, $v_{c_{i}}\left(x\right)$, and *v*_*S*_(*x*), respectively. The available volume profile for the complex $v_{c_{i}}\left(x\right)$, represents the probability that the complex has enough free volume to hop into the DNA mesh at position *x* and it equals the product of the probability of the two independent events of the enzyme and the substrate hopping into the DNA mesh [13], thus

$$\begin{array}{c} v_{c_{i}}\left(x\right)=v_{E_{i}}\left(x\right)v_{S}\left(x\right). \end{array}$$ (10)

Furthermore, we define the normalized available volume profiles as

$$\begin{array}{c} {\hat{v}}_{E_{i}}\left(x\right)=\frac{v_{E_{i}}\left(x\right)}{\int_{0}^{1}v_{E_{i}}\left(x\right)dx},\;{\hat{v}}_{c_{i}}\left(x\right)=\frac{v_{c_{i}}\left(x\right)}{\int_{0}^{1}v_{c_{i}}\left(x\right)dx},\;{\hat{v}}_{S}\left(x\right)=\frac{v_{S}\left(x\right)}{\int_{0}^{1}v_{S}\left(x\right)dx}.\; \end{array}$$ (11)

**Table 1:** The flux dynamics and the boundary conditions corresponding to (6) for each case of interest along with a core process example. Here *v*_*E*,*i*_(*x*), *v*_*S*_(*x*), and *v*_*c*,*i*_(*x*), are the available volume profiles of E_i_, S, and c_i_, respectively. The parameters $D_{E_{i}}$, $D_{c_{i}}$, and *D*_*s*_, are the enzyme, complex, and substrate diffusion coefficients, respectively, *ϵ* is a dimensionless parameter that captures the speed of diffusion (with respect to dilution). A species being spatially fixed translates to the flux being zero throughout the whole spatial domain. In Case II, for *i* = 1, …, *n*, $x_{i}^{*}\in \left(0,1\right)$ denotes the location of the fixed species E_i_. In Case III, $x_{s}^{*}\in \left(0,1\right)$ denotes the location of the fixed species S.

	Case I	Case II	Case III
	All species diffuse	Substrate diffuse and enzymes fixed	Enzymes diffuse and substrate fixed
**Dimensionless Flux**	$\begin{array}{cc} J(x,E_{i}) & =-\frac{1}{\epsilon }\chi_{E_{i}}v_{E_{i}}^{2}\frac{d}{dx}[\frac{E_{i}}{v_{E_{i}}}]\\ J(x,S) & =-\frac{1}{\epsilon }v_{S}^{2}\frac{d}{dx}[\frac{S}{v_{s}}]\\ J(x,c_{i}) & =-\frac{1}{\epsilon }\chi_{c_{i}}v_{c_{i}}^{2}\frac{d}{dx}[\frac{c_{i}}{v_{c_{i}}}] \end{array}$	$\begin{array}{cc} J(x,E_{i}) & =0\\ J(x,S) & =-\frac{1}{\epsilon }v_{s}^{2}\frac{d}{dx}[\frac{S}{v_{s}}]\\ J(x,c_{i}) & =0 \end{array}$	$\begin{array}{cc} J(x,E_{i}) & =-\frac{1}{\epsilon }v_{E_{i}}^{2}\frac{d}{dx}[\frac{E_{i}}{v_{E_{i}}}]\\ J(x,S) & =0\\ J(x,c_{i}) & =0 \end{array}$
**Boundary conditions**	$\begin{array}{c} J\left(0,E_{i}\right)=J\left(1,E_{i}\right)=0\\ J\left(0,c_{i}\right)=J\left(1,c_{i}\right)=0\\ J(0,S)=J(1,S)=0 \end{array}$	*J*(0, *S*) = *J*(1, *S*) = 0	*J*(0, *E*_*i*_) = *J*(1, *E*_*i*_) = 0
***ϵ***	(*μL*^2^)/*D*_*s*_	(*μL*^2^)/*D*_*s*_	$\begin{array}{c} \left(\mu L^{2}\right)/D_{E_{1}} \end{array}$
**Dimensionless diffusion**	$\begin{array}{c} \chi_{E_{i}}=D_{E_{i}}/D_{s},\chi_{c_{i}}=D_{c_{i}}/D_{s} \end{array}$	N/A	N/A
**Core process**	mRNAs binding ribosomes	RNAP binding several genes	Transcription factors binding promoter
**Location of fixed species**	N/A	$\begin{array}{c} x_{i}^{*} \end{array}$	$\begin{array}{c} x_{s}^{*} \end{array}$

## Results

### Time scale separation

In this section, we provide a time independent approximation of the BCF (8) in the limit of fast diffusion, which depends solely on the size of diffusing species, chromosome density profile ($\hat{\rho }\left(x\right)$ and r*), and the spatial localization of non-diffusing species. With this approximation, we can compute space averaged solutions in (7) without solving the PDEs in (6).

#### Reduced space averaged dynamics when diffusion is fast and fixed species are localized

**Fig 3:**
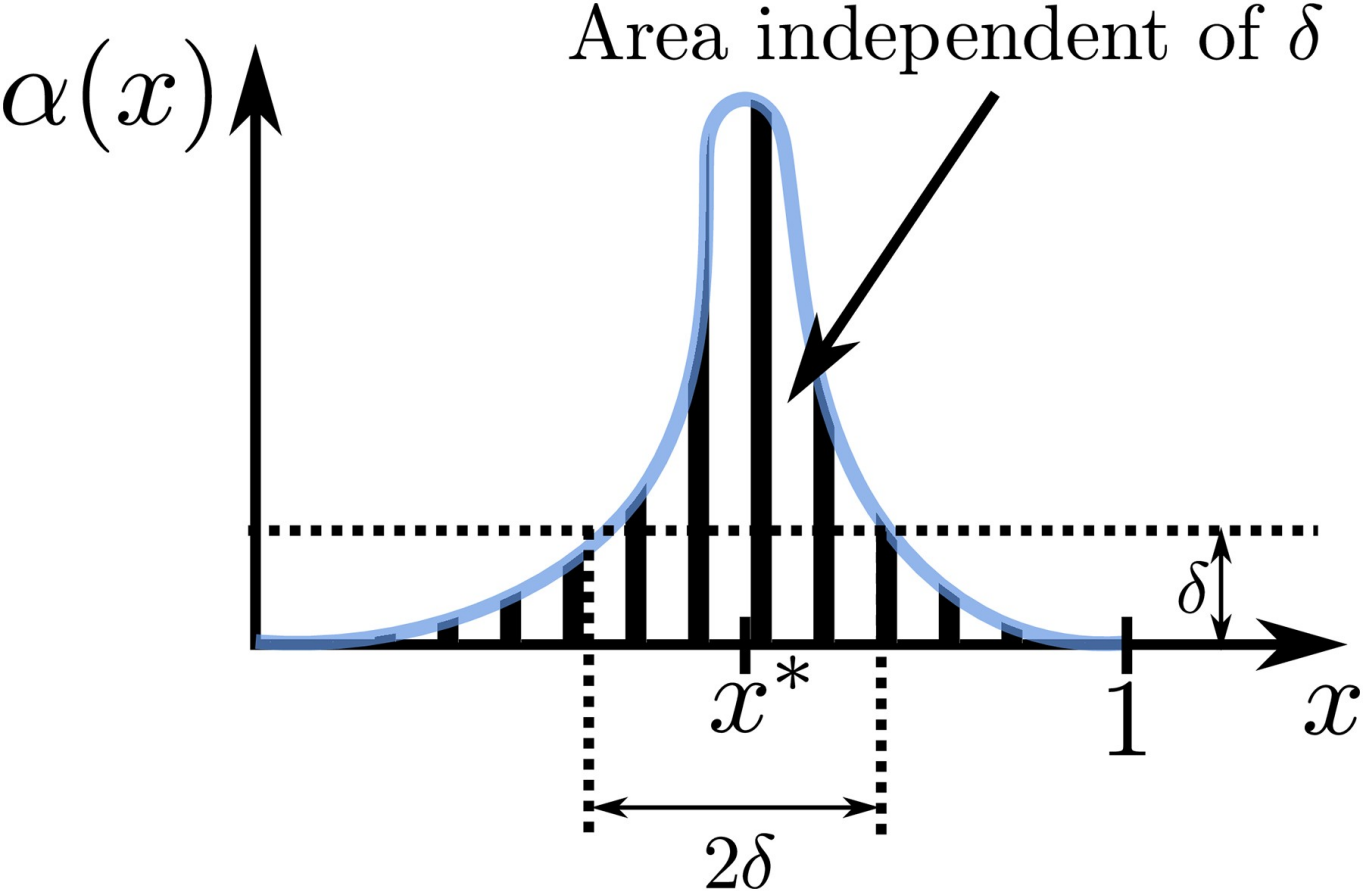
Graphical representation of localization of fixed species. The production rate *α*_*i*_(*t*, *x*) is assumed to be localized at $x_{i}^{*}$ if $\alpha_{i}\left(t,x\right)\leq \delta ,\forall x\notin \left[x_{i}^{*}-\delta ,x_{i}^{*}+\delta \right]$. We assume that the space averaged production ${\bar{\alpha }}_{i}\left(t\right)=\int_{0}^{1}\alpha_{i}\left(t,x\right)dx$ is independent of *δ*.

For Case II and Case III of Table 1, in which one of the reacting species is fixed, we assume that the *fixed species is spatially localized* to a small space, that is, we have the situation depicted in Fig 3 (see S1 Text: Assumption 3 for the mathematical definition). Practically, for Case II, spatial localization at $x_{i}^{*}$ requires that the production rate *α*_*i*_(*t*, *x*) of the fixed species is smaller than some small threshold *δ* when *x* is outside the interval $\left[x_{i}^{*}-\delta ,x_{i}^{*}+\delta \right]$ for all time and that the space averaged production rate is ${\bar{\alpha }}_{i}\left(t\right)$ independent of *δ* (similarly for Case III, $x_{s}^{*}$, and *α*_*s*_(*t*, *x*)). From a biological perspective, having the space averaged production rate independent of *δ* is consistent with the fact that the total amount of DNA in the cell is independent of where the DNA is concentrated. Note that *δ* is a parameter that controls the amount of localization, such that *δ* ≪ 1 implies the production of spatially fixed species being localized to a small region. Let *ϵ* be as in Table 1 that appears in (6), the following definition will provide the candidate reduced model that approximates (7) well when *ϵ* ≪ 1 and *δ* ≪ 1. Recall that *ϵ* is a dimensionless parameter that captures the speed of diffusion (with respect to dilution).

Let $x_{i}^{*}$ and $x_{s}^{*}$ be the location of the fixed species for Case II and Case III, respectively (see Table 1). For *i* = 1, …, *n*, we define the *reduced space-averaged dynamics* as

$$\begin{array}{cc} & \frac{d{\hat{\bar{E}}}_{i}\left(t\right)}{dt}={\bar{\alpha }}_{i}\left(t\right)-\frac{1}{\eta_{i}}[{\hat{\bar{E}}}_{i}\left(t\right)\hat{\bar{S}}\left(t\right)\frac{{\tilde{a}}_{i}\theta_{i}^{*}}{{\tilde{d}}_{i}}-{\hat{\bar{c}}}_{i}\left(t\right)]-\left(\gamma_{i}+1\right)\left({\hat{\bar{E}}}_{i}\left(t\right)+{\hat{\bar{c}}}_{i}\left(t\right)\right),\\ & \frac{d{\hat{\bar{c}}}_{i}\left(t\right)}{dt}=\frac{1}{\eta_{i}}[{\hat{\bar{E}}}_{i}\left(t\right)\hat{\bar{S}}\left(t\right)\frac{{\tilde{a}}_{i}\theta_{i}^{*}}{{\tilde{d}}_{i}}-{\hat{\bar{c}}}_{i}\left(t\right)],\\ & \frac{d\bar{S}\left(t\right)}{dt}={\bar{\alpha }}_{s}\left(t\right)-\sum_{j=1}^{n}\frac{1}{\eta_{j}}[{\hat{\bar{E}}}_{j}\left(t\right)\hat{\bar{S}}\left(t\right)\frac{{\tilde{a}}_{j}\theta_{j}^{*}}{{\tilde{d}}_{j}}-{\hat{\bar{c}}}_{i}\left(t\right)]-\left(\gamma_{s}+1\right)\left(\hat{\bar{S}}\left(t\right)+\sum_{j=1}^{n}{\hat{\bar{c}}}_{j}\left(t\right)\right), \end{array}$$ (12)

$$\begin{array}{c} \theta_{i}^{*}=\{\begin{array}{cc} \int_{0}^{1}{\hat{v}}_{E_{i}}\left(x\right){\hat{v}}_{S}\left(x\right)dx & \text{for}\;\text{Case}\;\text{I}\\ {\hat{v}}_{S}\left(x_{i}^{*}\right) & \text{for}\;\text{Case}\;\text{II}\\ {\hat{v}}_{E_{i}}\left(x_{s}^{*}\right) & \text{for}\;\text{Case}\;\text{III} \end{array}, \end{array}$$ (13)

where ${\hat{\bar{E}}}_{i}\left(0\right)={\bar{E}}_{i}\left(0\right)$, $\hat{\bar{S}}\left(0\right)=\bar{S}\left(0\right)$, ${\hat{\bar{c}}}_{i}\left(0\right)={\bar{c}}_{i}\left(0\right)$, as given by (7), and ${\hat{v}}_{E_{i}}\left(x\right)$, ${\hat{v}}_{c_{i}}\left(x\right)$, and ${\hat{v}}_{S}\left(x\right)$ are given by (11). Then, we have the following main result of this paper (see S1 Text: Theorem 3 for a formal statement with the proof).

**Result** 1. *Consider system* (6) *and let* ***z***(*t*, *x*) = [*E*_1_(*t*, *x*), …, *E*_*n*_(*t*, *x*), *c*_1_(*t*, *x*), …, *c*_*n*_(*t*, *x*), *S*(*t*, *x*)]^*T*^ *with* $\bar{z}\left(t\right)=\int_{0}^{1}z\left(t,x\right)dx.$ *Consider system* (12) *and let* $\hat{\bar{z}}\left(t\right)={\left[{\hat{\bar{E}}}_{1}\left(t\right),\ldots ,{\hat{\bar{E}}}_{n}\left(t\right),{\hat{\bar{c}}}_{1}\left(t\right),\ldots ,{\hat{\bar{c}}}_{n}\left(t\right),\hat{\bar{S}}\left(t\right)\right]}^{T}.$ *Then*, *for all t* ≥ 0 *and ϵ*, *δ sufficiently small*

$$\begin{array}{c} ∥\bar{z}\left(t\right)-\hat{\bar{z}}\left(t\right)∥=\{\begin{array}{cc} O(\epsilon ) & for\;Case\;I\\ O(\epsilon )+O(\delta ) & for\;Case\;II,\;III \end{array}. \end{array}$$ (14)

By virtue of this result, we can use the simple and convenient ODE model in Eq (12) to describe the space-averaged dynamics of the PDE system (6). In particular, from (12) it appears that spatial effects are lumped into the BCF approximation $\theta_{i}^{*}$. Therefore, in order to determine how spatial heterogeneity affects system dynamics, it is sufficient to analyze how dynamics is affected by parameter $\theta_{i}^{*}$ and how the expression of $\theta_{i}^{*}$ is, in turn, affected by spatial localization and molecule size (see (13) and (2)).

*Remark* 1. As discussed in S1 Text: Section 1, as *ϵ* → 0^+^, the spatial profile of diffusing molecules approaches that of their available volume profile after a fast transient, that is,

$$\begin{array}{c} \underset{\text{Case}\;\text{I}\;\text{and}\;\text{Case}\;\text{III}}{\underset{︸}{E_{i}\left(t,x\right)\approx {\bar{E}}_{i}\left(t\right){\hat{v}}_{E,i}\left(x\right)}},\;\underset{\text{Case}\;\text{I}}{\underset{︸}{c_{i}\left(t,x\right)\approx {\bar{c}}_{i}\left(t\right){\hat{v}}_{c,i}\left(x\right)}},\;\underset{\text{Case}\;\text{I}\;\text{and}\;\text{Case}\;\text{II}}{\underset{︸}{S\left(t,x\right)\approx \bar{S}\left(t\right){\hat{v}}_{S}\left(x\right)}},\; \end{array}$$

for the other spatially fixed species we have that their concentrations are localized in a manner as their production terms.

The consequence of Remark 1 is that knowledge of the space averaged dynamics from system (12) also leads to knowledge of the spacial profiles of the species within the cell. This information is used to propose a method to estimate the BCF from experimental data (See S1 Text: Section 7).

*Remark* 2. The approximation result holds for *ϵ* ≪ 1, that is, diffusion is much faster than any other time scales in (6). However, in S1 Text: Section 1.4, we motivate why the approximation should still hold (for which the relationship (10) is key) if $\eta_{i}/\epsilon =O\left(1\right)$ (binding and unbinding between E_i_ and S occurs at a similar timescale as diffusion), and confirmed via numerical simulations for the upcoming biological examples.

The BCF $\theta_{i}^{*}$ in (13) is temporally constant and thus the reduced model has the same dimensionality as the well-mixed model (9), yet captures the role of spatial heterogeneity in the interactions between cellular species. Therefore, $\theta_{i}^{*}$ is a practical and accurate approximation of the BCF when *ϵ* ≪ 1 (sufficient for Case I) and *δ* ≪ 1 (needed for Cases II-III).

#### Dependence of the BCF on species size and localization

When diffusion is fast and the expression of spatially fixed species is localized, the BCF is well approximated by $\theta_{i}^{*}$ given in (13). Substituting (2) into (13) and denoting the radius of gyration of E_i_ and S by *r*_*e*,*i*_ and *r*_*s*_, respectively, we can rewrite $\theta_{i}^{*}$ as

$$\begin{array}{c} \theta_{i}^{*}=\{\begin{array}{cc} \frac{\int_{0}^{1}e^{-\frac{r_{e,i}^{2}+r_{s}^{2}}{{\left(r^{*}\right)}^{2}}\hat{\rho }\left(x\right)}dx}{\left[\int_{0}^{1}e^{-{\left(r_{e,i}/r^{*}\right)}^{2}\hat{\rho }\left(x\right)}dx\right]\left[\int_{0}^{1}e^{-{\left(r_{s}/r^{*}\right)}^{2}\hat{\rho }\left(x\right)}dx\right]} & \text{for}\;\text{Case}\;\text{I}\\ \frac{e^{-{\left(r_{s}/r^{*}\right)}^{2}\hat{\rho }\left(x_{i}^{*}\right)}}{\int_{0}^{1}e^{-{\left(r_{s}/r^{*}\right)}^{2}\hat{\rho }\left(x\right)}dx} & \text{for}\;\text{Case}\;\text{II}\\ \frac{e^{-{\left(r_{e,i}/r^{*}\right)}^{2}\hat{\rho }\left(x_{s}^{*}\right)}}{\int_{0}^{1}e^{-{\left(r_{e,i}/r^{*}\right)}^{2}\hat{\rho }\left(x\right)}dx} & \text{for}\;\text{Case}\;\text{III} \end{array}. \end{array}$$ (15)

From (15), we observe that $\theta_{i}^{*}$, depends on the spatial localization of spatially fixed species (i.e,. $x_{i}^{*}$ and $x_{s}^{*}$), the radius of gyration of diffusing species, r* (2), and the nominalized local density of DNA length $\hat{\rho }\left(x\right)$.

**Fig 4:**
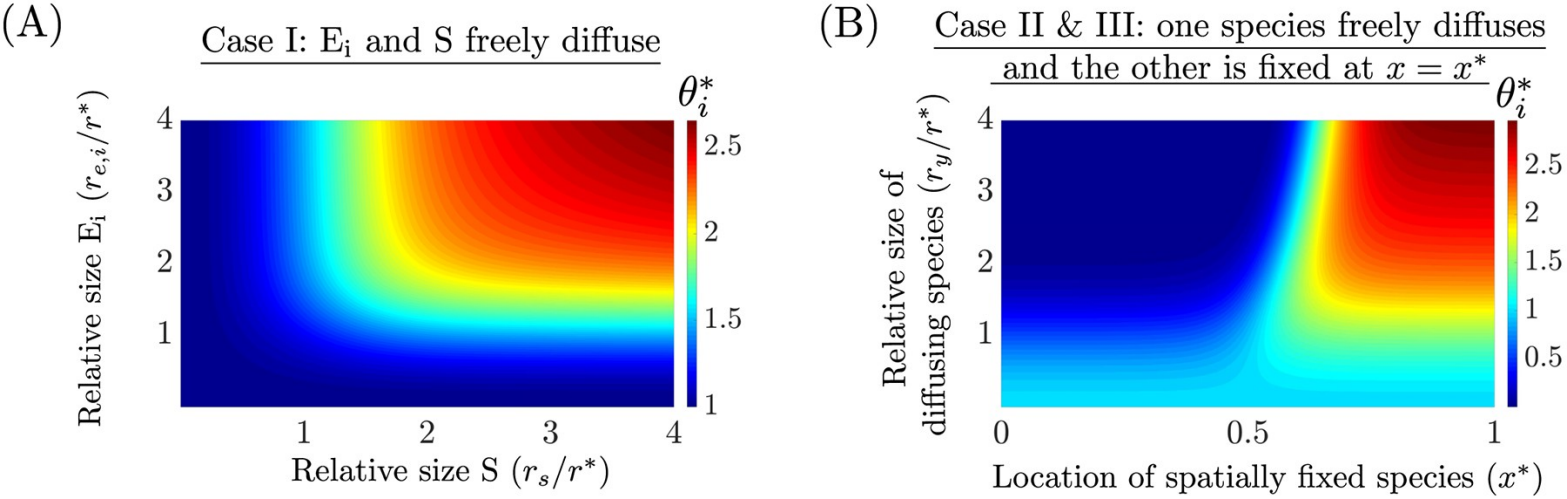
The BCF in the limit of fast diffusion and localization of spatially fixed species. Approximation of the BCF denoted by $\theta_{i}^{*}$ (15) is provided for Case I and for Case II/Case III. (A) For Case I, where E_i_ and S both freely diffuse, *θ*_*i*_ ≥ 1 and increases when both the size of E_i_ (*r*_*E*,*i*_) and S (*r*_*s*_) are sufficiently large (with respect to r*). (B) For Case II and III, where one of the species diffuses (size *r*_*y*_) and the other is fixed at *x* = x*, $\theta_{i}^{*}$ is different from unity when the radii of the diffusion species is sufficiently large. We observe that $\theta_{i}^{*}<1$ for x* ≤ 0.4 and appears to approach zero near x* = 0 for large *r*_*y*_/r*. Similarly, for x* ≥ 0.65, $\theta_{i}^{*}>1$. Between 0.4 ≤ x* ≤ 0.65 there exists a region that $\theta_{i}^{*}=1$ for all *r*_*y*_/r*.

Using (15), we graphically illustrate the dependence of $\theta_{i}^{*}$ on *r*_*E*,*i*_, *r*_*s*_, r*, $x_{i}^{*}$ and $x_{s}^{*}$ in Fig 4. By analyzing Fig 4, we observe the following:

**Case I**: the BCF is always greater than or equal to that of the well-mixed model (9) (where $\theta_{i}^{*}=1$ for all *i*) and this discrepancy increases with the size of E_i_ and S. Intuitively, as the size of E_i_ and S increases, they are pushed out of the chromosome and co-localize near the cell poles, thus they are confined to a smaller volume to interact and hence their effective binding strength increases. If only one of the species is large (with respect to r*), while the other one is small, then the large species will be ejected from the chromosome and thus will not be homogeneously distributed throughout the cell, however $\theta_{i}^{*}\approx 1$, and thus a well-mixed model is valid despite this spatial heterogeneity.
**Case II and III**: where one of the species diffuses (size *r*_*y*_) and the other is fixed at *x* = x*, the BFC is different from unity when *r*_*y*_ is sufficiently large. We observe that $\theta_{i}^{*}<1$ for x* ≤ 0.4 and appears to approach zero near x* = 0 for large *r*_*y*_/r*. Similarly, for x* ≥ 0.65, $\theta_{i}^{*}>1$. This occurs because as the size of the diffusing species increases, the species is ejected from the chromosome onto the cell poles and therefore it is more likely to interact with species fixed at the cell-poles than those near mid-cell. Between 0.4 ≤ x* ≤ 0.65 there exists a region where $\theta_{i}^{*}=1$ for all *r*_*y*_/r*. This provides additional evidence that a well-mixed model may be appropriate despite severe intracellular heterogeneity.

When $\hat{\rho }\left(x\right)$ is assumed to be a step function, the upper bound for $\theta_{i}^{*}$ is 1/Δ*x* for Case I-III, as derived in S1 Text: Section 2, where Δ*x* is the distance between the end of the chromosome and the cell poles as shown in Fig 1. Furthermore, the lower bound for Case I was unity and for Case II-III it was zero.

The value of the BCF provides a measure to determine the extent to which spatial effects modulate the biomolecular dynamics. Therefore, an experimental method to estimate the BCF is desirable. In S1 Text: Section 7, we propose such a method that only requires knowledge of Δ*x* and of the value of concentration of freely diffusing species inside and outside the nucleoid.

In S1 Text: Section 9, we consider how the BCF can vary temporally as the cell divides and the chromosome density shifts from being concentrated primarily near mid-cell to quarter-cell. We demonstrate that the BCF can vary by over 50% in time for the case where one species is stationary and localized near mid-cell. Furthermore, in S1 Text: Section 10, we show how the BCF is affected when we consider exclusion effects from the DNA of a pole localized high copy plasmid. We show that for the case where both reactant freely diffuse, the BCF decreases as the amount of plasmid DNA increases. For the case where one reactant is spatially fixed and the other freely diffuses, we show that the BCF decreases for a species localized at the cell poles and increases for a species localized near quarter-cell, as the amount of plasmid DNA increases.

### Application to core processes and genetic circuits

In this section we apply the results of the time scale separation analysis to determine the effects of intracellular heterogeneity on core processes, such as transcription and translation, and on genetic circuit behavior.

#### Application of the reduced ODE model to transcription and translation

In this section, we investigate how and the extent to which intracellular heterogeneity affects the core biological processes of transcription and translation, which are responsible for protein production. We model a gene (D) being transcribed by RNAP (S) to form a DNA-RNAP complex (c_s_) to produce mRNA (m). The mRNA is then translated by ribosomes (R) to form mRNA-ribosome complex (c_m_) which produces protein P. The chemical reactions are given by

$$\begin{array}{c} \underset{\text{transcription},\;\text{Case}\;\text{II}}{\underset{︸}{D+S\overset{a_{s}}{\underset{d_{s}}{⇌}}c_{s}\overset{\kappa_{s}}{⟶}m+S+D}},\;\underset{\text{translation},\;\text{Case}\;\text{I}}{\underset{︸}{m+R\overset{a_{m}}{\underset{d_{m}}{⇌}}c_{m}\overset{\kappa_{m}}{⟶}P+R+m}},\;P\overset{\mu }{⟶}\emptyset ,\;\\ c_{s}\overset{\mu }{⟶}\emptyset ,\;\emptyset \overset{\alpha_{s}}{⟶}S\overset{\mu }{⟶}\emptyset ,\;m\overset{\gamma +\mu }{⟶}\emptyset ,\;c_{m}\overset{\gamma }{⟶}R\;c_{m}\overset{\mu }{⟶}\emptyset \;\emptyset \overset{\alpha_{r}}{⟶}R\overset{\mu }{⟶}\emptyset , \end{array}$$ (16)

where *a*_*s*_ and *d*_*s*_ are the association and dissociation rate constants, respectively, between RNAP and the gene D, *κ*_*s*_ is the catalytic rate constant of formation of mRNA m, *a*_*m*_ and *d*_*m*_ are the association and dissociation rate constants, respectively, between ribosomes and mRNA, *κ*_*m*_ is the catalytic rate constant of formation of protein P, *α*_*s*_ is the production rate of RNAP, *α*_*r*_ is the ribosome production rate, *μ* is the cell growth rate constant (set to unity in our nondimensionalization), and *γ* is the mRNA degradation rate constant. The transcription reaction is in the form of Case II (Table 1) since the gene does not freely diffuse and the RNAP freely diffuses. The translation process falls under Case I, since both mRNA and ribosomes freely diffuse. We assume that the total concentration of D is conserved, so that *D*_*T*_(*x*) = *D*(*t*, *x*) + *c*_*s*_(*t*, *x*) and that *D*_*T*_(*x*) is localized at *x* = x*. From (12), the dimensionless reduced space averaged dynamics corresponding to (16) are given by

$$\begin{array}{cc} \frac{d{\bar{c}}_{s}\left(t\right)}{dt} & =a_{s}\theta_{s}^{*}\bar{D}\left(t\right)\bar{S}\left(t\right)-\left(d_{s}+\kappa_{s}+1\right){\bar{c}}_{s}\left(t\right),\\ \frac{d\bar{S}\left(t\right)}{dt} & ={\bar{\alpha }}_{s}\left(t\right)-a_{s}\theta_{s}^{*}\bar{D}\left(t\right)\bar{S}\left(t\right)+\left(\kappa_{s}+d_{s}\right)\bar{c}\left(t\right)-\bar{S}\left(t\right),\\ \frac{d\bar{m}\left(t\right)}{dt} & =\kappa_{s}{\bar{c}}_{s}\left(t\right)-a_{m}\theta_{R}^{*}\bar{R}\left(t\right)\bar{m}\left(t\right)+\left(d_{m}+\kappa_{m}\right)\bar{c}\left(t\right)-\left(\gamma +1\right)\bar{m}\left(t\right)\\ \frac{d{\bar{c}}_{m}\left(t\right)}{dt} & =a_{m}\theta_{R}^{*}\bar{R}\left(t\right)\bar{m}\left(t\right)-\left(d_{m}+\kappa_{m}+1+\gamma \right)\bar{c}\left(t\right),\\ \frac{d\bar{R}\left(t\right)}{dt} & ={\bar{\alpha }}_{r}\left(t\right)-a_{m}\theta_{R}^{*}\bar{R}\left(t\right)\bar{m}\left(t\right)+\left(\gamma +d_{m}+\kappa_{m}\right){\bar{c}}_{m}\left(t\right)-\bar{R}\left(t\right),\\ \frac{d\bar{P}\left(t\right)}{dt} & =\kappa_{m}{\bar{c}}_{m}\left(t\right)-\bar{P}\left(t\right). \end{array}$$ (17)

Concentration variables are nondimensionalized with respect to the total steady state space averaged RNAP (${\bar{S}}_{T}\left(\infty \right)={\bar{\alpha }}_{s}\left(\infty \right)/\mu$), since this quantity is a readily available in the literature. Letting *r*_*s*_, *r*_*m*_, and *r*_*R*_, be the radius of gyration of RNAP, mRNA, and ribosomes, respectively, we compute the BCF’s via (13) and (2),

$$\begin{array}{cc} \theta_{s}^{*} & ={\hat{v}}_{s}\left(x^{*}\right)=\frac{e^{-{\left(r_{s}/r^{*}\right)}^{2}\hat{\rho }\left(x^{*}\right)}}{\int_{0}^{1}e^{-{\left(r_{s}/r^{*}\right)}^{2}\hat{\rho }\left(x\right)}dx},\\ \theta_{R}^{*} & =\int_{0}^{1}{\hat{v}}_{m}\left(x\right){\hat{v}}_{R}\left(x\right)dx=\frac{\int_{0}^{1}e^{-\frac{r_{m}^{2}+r_{R}^{2}}{{\left(r^{*}\right)}^{2}}\hat{\rho }\left(x\right)}dx}{\left[\int_{0}^{1}e^{-{\left(r_{m}/r^{*}\right)}^{2}\hat{\rho }\left(x\right)}dx\right]\left[\int_{0}^{1}e^{-{\left(r_{R}/r^{*}\right)}^{2}\hat{\rho }\left(x\right)}dx\right]}, \end{array}$$ (18)

where ${\hat{v}}_{s}\left(x\right)=\frac{v_{s}\left(x\right)}{\int_{0}^{1}v_{s}\left(x\right)dx}$, ${\hat{v}}_{m}\left(x\right)=\frac{v_{m}\left(x\right)}{\int_{0}^{1}v_{m}\left(x\right)dx}$, ${\hat{v}}_{R}\left(x\right)=\frac{v_{R}\left(x\right)}{\int_{0}^{1}v_{R}\left(x\right)dx}$, and ${\hat{v}}_{c}\left(x\right)=\frac{v_{c}\left(x\right)}{\int_{0}^{1}v_{c}\left(x\right)dx}$ are the normalized available volume profiles of RNAP, mRNA, ribosomes, and of the mRNA-ribosome complex, respectively. Recall that the quantity (r*)^2^ is inversely proportional to the total DNA length per volume. We now consider the steady state behavior of system (17) by equating the time derivatives to zero. Specifically, we are interested in how the steady state levels of produced mRNA and protein are affected by $\theta_{s}^{*}$ and $\theta_{r}^{*}$ and, hence, how they depend on spatial quantities such as *r*_*s*_/r*r_*m*_/r*, *r*_*R*_/r*, and x*.

**Total mRNA steady state level**: We are interested in investigating the role of spatial effects on the binding between RNAP and the DNA and thus on mRNA production. Here we analyze the steady state total mRNA levels (${\bar{m}}_{T}=\bar{m}+{\bar{c}}_{m}$) of (17) rather than the free amount of mRNA (*m*), since ${\bar{m}}_{T}$ is independent of $\theta_{R}^{*}$ as shown by

$$\begin{array}{c} {\bar{m}}_{T}=\kappa_{s}{\bar{c}}_{s}/\left(\gamma +1\right)\text{with}\;\;{\bar{c}}_{s}={\bar{D}}_{T}\frac{\bar{S}\theta_{S}^{*}/K_{s}}{1+\bar{S}\theta_{S}^{*}/K_{s}}, \end{array}$$ (19)

where ${\bar{D}}_{T}=\int_{0}^{1}D_{T}\left(x\right)dx$, *K*_*s*_ = *d*_*s*_/*a*_*s*_, *K*_*R*_ = *d*_*m*_/*a*_*m*_. If $\theta_{S}^{*}=1$ in (19), then the predicted total mRNA steady state level will be identical to that of a well-mixed model (as in (9)). From (18) and Fig 4B, if the RNAP radius of gyration is sufficiently large with respect to r* then $\theta_{S}^{*}$ may be different from unity (depending on x*), in which case spatial effects arise. If the DNA is localized near mid-cell (x* ≈ 0), then it implies that $\theta_{S}^{*}<1$ from Fig 4B and, as a consequence, a decreased steady state total mRNA level will result. Furthermore, for very large values of *r*_*s*_/r* and x* ≈ 0 we have that $\theta_{S}^{*}\to 0$ and the total mRNA steady state levels will approach zero. Similarly, if the DNA is localized near the cell-poles (x* ≈ 1), then it implies that $\theta_{S}^{*}>1$ from Fig 4B and, as a consequence, an increased steady state total mRNA level. This phenomenon occurs because as the excluded volume effects of RNAP are amplified (large *r*_*s*_/r*), RNAP will localize primarily in the cell poles and hence transcribe pole-localized DNA more efficiently than DNA near mid-cell (or any region where the local chromosome density is high). When designing genetic circuits, a plasmid backbone is chosen to provide a certain DNA copy number, however the backbone also determines where in the cell the plasmid localizes [9–11]. Therefore, based on our results, localization also affects steady state total mRNA level. If instead of introducing the DNA via a plasmid, the DNA is integrated directly into the chromosome, then the location of integration site should be a parameter to consider.

**Fig 5:**
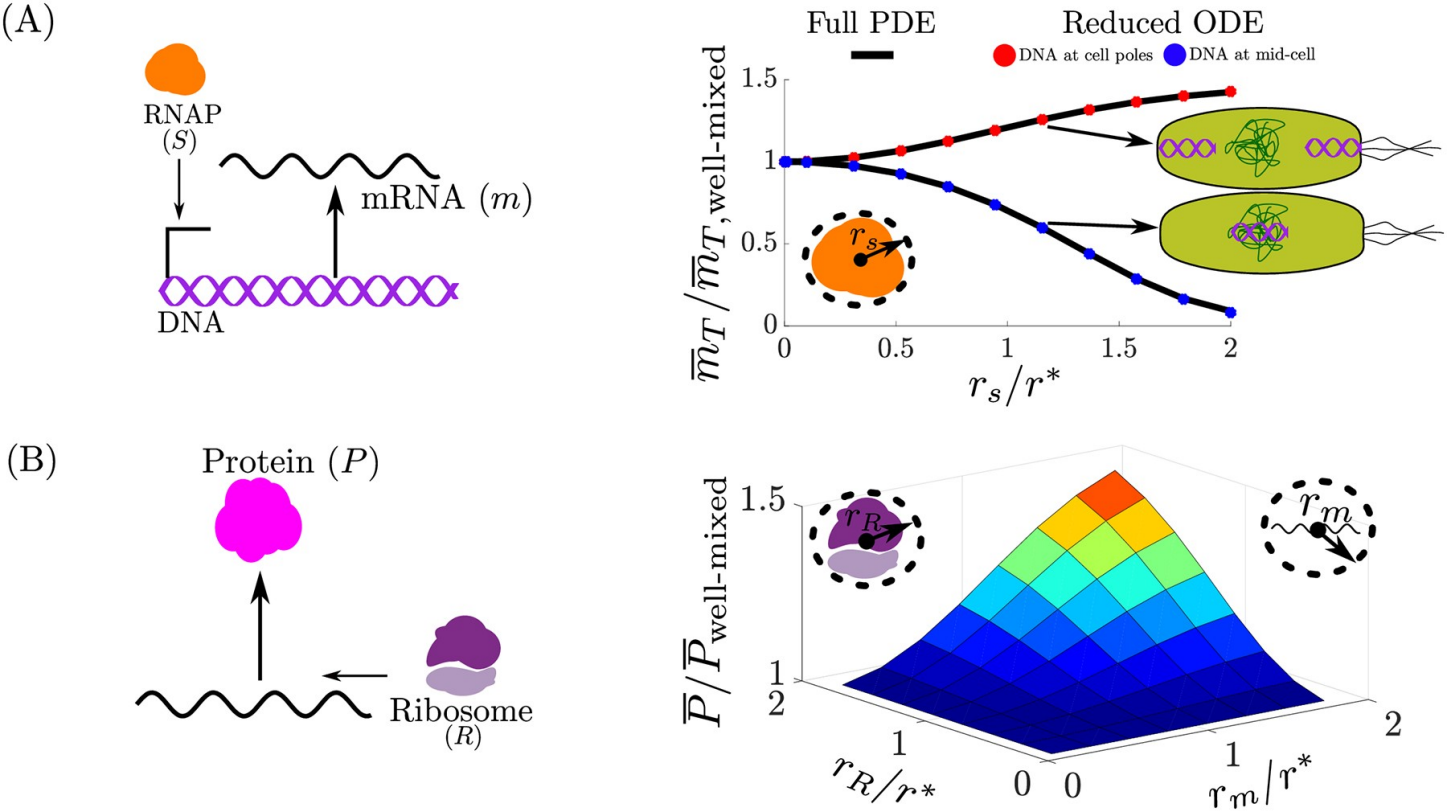
Spatial heterogeneity effects on steady state total mRNA and protein levels. (A) The space averaged total mRNA (${\bar{m}}_{T}$) concentration predicted by the full PDE model (S1 Text: Equation 34) and the reduced ODE model (17) normalized by that of the well-mixed model (${\bar{m}}_{T,\text{well}-\text{mixed}}$) as the size of the RNAP (*r*_*s*_) varies with respect to r*. With respect to the well-mixed model, the amount of mRNA decreases (increases) when the DNA is localized near mid-cell (cell poles). (B) The space averaged protein concentration ($\bar{P}$) predicted by the full PDE model (S1 Text: Equation 34) normalized by that of the well-mixed model (${\bar{P}}_{\text{well}-\text{mixed}}$) as the size of the of mRNA (*r*_*m*_) and ribosome (*r*_*R*_) varies with respect to r*. The amount of protein increases when both the mRNA and ribosome size increases. We set *r*_*s*_/r* = 1 × 10^−3^, such that *θ*_*S*_ ≈ 1 and thus the result is independent of the spatial location where the gene is expressed. We refer to the well-mixed model as (17) with (18) given by $\theta_{S}^{*}=1$ and $\theta_{R}^{*}=1$. The parameter values and full simulation details are provided in S1 Text: Section 3.

Fig 5A shows the behavior of the steady state total mRNA level as a function of *r*_*s*_/r* and of the location of the transcribed gene, when compared to the level predicted by the well mixed model. Simulations confirm that total mRNA levels are higher for pole localized genes than those near mid-cell and that the discrepancy increases with the size of RNAP relative to r*. The agreement between the full PDE model (S1 Text: Equation 34) and the reduced ODE model (17) provides numerical validation of the model reduction results (explicitly shown in S1 Text: Fig D). In S1 Text: Fig B, the transient response corresponding to Fig 5, for which the full-PDE and reduced models agree. Furthermore, in S1 Text: Fig B, we also verify that as the size of RNAP increases, it is indeed ejected from the chromosome and adopts its available volume profile (Remark 1). Furthermore, in S1 Text: Fig C, we demonstrate that these results hold independent of the binding and unbinding speed between RNAP and DNA (Remark 2). In S1 Text: Section 8, we propose an experimental method to test the hypothesis that mid-cell genes are transcribed less effectively than pole localized genes.

In [26], it was estimated that *r*_*s*_ = 6.5±0.1 nm, which implies that *r*_*s*_/r* ≈ 0.3. From Fig 4B, this implies that $\theta_{s}^{*}\approx 1.06$ when the DNA is at the cell poles and $\theta_{s}^{*}\approx 0.94$ when the DNA is near mid-cell, thus the we expect the binding strength between RNAP and the DNA to deviate by 6% from that of a well-mixed model. From (19), if $S\theta_{S}^{*}/K_{s}<<1$, then ${\bar{m}}_{T}=\frac{\theta_{S}^{*}\kappa_{s}{\bar{D}}_{T}\bar{S}}{K_{s}\left(\gamma +1\right)};$ thus in this regime the mRNA concentration is proportional to $\theta_{S}^{*}$. So we expect at most a 6% difference in steady state mRNA concentration with respect to what is predicted by a well-mixed model.

**Protein steady state level**: The steady state protein levels of (17) are given by

$$\begin{array}{c} \bar{P}=\kappa_{m}{\bar{c}}_{m},\;\text{with}\;\;{\bar{c}}_{m}\left(t\right)={\bar{m}}_{T}\frac{\bar{R}/K_{R}\theta_{R}^{*}}{1+\bar{R}/K_{R}\theta_{R}^{*}}. \end{array}$$ (20)

From (19) and (20), if $\theta_{S}^{*}=1$ and $\theta_{R}^{*}=1$, then protein steady state level will be identical to that of a well-mixed model. From (18) and Fig 4A, we conclude that $\theta_{R}^{*}\geq 1$ and increases with *r*_*s*_/r* and *r*_*m*_/r*. Increasing *r*_*s*_/r* and *r*_*m*_/r* implies that the ribosomes and mRNA are further excluded from the chromosome onto the cell poles. Once localized at the cell-poles, the mRNA and ribosomes are more likely to bind. Fig 5B shows the behavior of the steady state protein levels as a function of *r*_*m*_ and *r*_*R*_, when compared to the level predicted by the well mixed-model for the full PDE model (S1 Text: Equation 34). Simulations confirm that protein levels with respect to a well-mixed model increases when both the mRNA and ribosome size are sufficiently large. In S1 Text: Fig F, we show that the reduced ODE model (17) is within 2% of the full PDE model (S1 Text: Equation 34) for the result in Fig 5B. In S1 Text: Fig E, we show the transient response corresponding to Fig 5, for which the full-PDE and reduced model agree. Furthermore, in S1 Text: Fig E, we verify that as the size of ribosome and mRNA increase, they are ejected from the chromosome and and become distributed according to their available volume profile (Remark 1). Finally, in S1 Text: Figs G and F, we demonstrate that these results hold independent of the binding and unbinding speed between ribosomes and mRNA (Remark 2).

It is well known that most mRNA-ribosome complexes exists in configurations with multiple ribosomes bounded (polysomes) [27, 28]. To capture the prevalence of these polysomes, we model the translation process accounting for the fact that one mRNA can be bound to multiple ribosomes. We first model the mRNA binding simultaneously to *N*_*r*_ − 1 ribosomes to form the c_l_ complex, to which another ribosome binds to to form the fully loaded c_t_ complex. The leading ribosome with a complete peptide is released from c_t_ at a rate *κ*_*t*_ to yield protein P. This is described by the following set of biochemical reactions:

$$\begin{array}{c} \underset{\text{ribosome}\;\text{loading}}{\underset{︸}{m+\left(N_{r}-1\right)R\overset{a_{l}}{\underset{d_{l}}{⇌}}c_{l}}},\;c_{l}+R\overset{a_{t}}{\underset{d_{t}}{⇌}}\underset{\text{peptide}\;\text{release}}{\underset{︸}{c_{t}\overset{\kappa_{t}}{⟶}P+R+c_{l}}}. \end{array}$$ (21)

While the ribosome loading reaction in (21) is not in the form of the chemical reactions (1), which assume bimolecular reactions, we can nevertheless apply our results as follows (see S1 Text: Section 3.1 for details). Specifically, ribosome and mRNA profiles will still approach their available volume profiles (Remark 1), that is, $R\left(t,x\right)\approx \bar{R}\left(t\right){\hat{v}}_{r}\left(x\right)$, $m\left(t,x\right)\approx \bar{m}\left(t\right){\hat{v}}_{m}\left(x\right)$, and $c_{l}\left(t,x\right)\approx {\bar{c}}_{l}\left(t\right){\hat{v}}_{c_{l}}\left(x\right)$ where $v_{c_{l}}\left(x\right)=v_{r}^{N_{r}-1}\left(x\right)v_{m}\left(x\right)$ (recall (10)) and ${\hat{v}}_{c_{l}}\left(x\right)=v_{c_{l}}\left(x\right)/\left[\int_{0}^{1}v_{c_{l}}dx\right]$. This is verified through simulations in S1 Text: Fig I. By virtue of the reactants in (21) mirroring their available volume profiles and (8), we can approximate the BCF of the loading $\theta_{l}^{*}$ and translation $\theta_{t}^{*}$ reactions in (21), given as

$$\begin{array}{c} \theta_{l}^{*}=\frac{\int_{0}^{1}v_{m}\left(x\right)v_{R}^{N_{r}-1}\left(x\right)dx}{\left[\int_{0}^{1}v_{m}\left(x\right)dx\right]{\left[\int_{0}^{1}v_{R}\left(x\right)dx\right]}^{N_{r}-1}}=\frac{\int_{0}^{1}e^{-\frac{r_{m}^{2}+{\left(\sqrt{N_{r}-1}r_{R}\right)}^{2}}{{\left(r^{*}\right)}^{2}}\hat{\rho }\left(x\right)}dx}{\left[\int_{0}^{1}e^{-{\left(r_{m}/r^{*}\right)}^{2}\hat{\rho }\left(x\right)}dx\right]{\left[\int_{0}^{1}e^{-{\left(r_{R}/r^{*}\right)}^{2}\hat{\rho }\left(x\right)}dx\right]}^{N_{r}-1}}. \end{array}$$

$$\begin{array}{c} \theta_{t}^{*}=\frac{\int_{0}^{1}v_{c_{l}}\left(x\right)v_{R}\left(x\right)dx}{\left[\int_{0}^{1}v_{c_{l}}\left(x\right)dx\right]\left[\int_{0}^{1}v_{R}\left(x\right)dx\right]}=\frac{\int_{0}^{1}e^{-\frac{r_{m}^{2}+{\left(\sqrt{N_{r}}r_{R}\right)}^{2}}{{\left(r^{*}\right)}^{2}}\hat{\rho }\left(x\right)}dx}{\left[\int_{0}^{1}e^{-\frac{r_{m}^{2}+{\left(\sqrt{N_{r}-1}r_{R}\right)}^{2}}{{\left(r^{*}\right)}^{2}}\hat{\rho }\left(x\right)}dx\right]\left[\int_{0}^{1}e^{-{\left(r_{R}/r^{*}\right)}^{2}\hat{\rho }\left(x\right)}dx\right]}. \end{array}$$

In S1 Text: Fig I, we show computationally that $\theta_{l}^{*}$ and $\theta_{t}^{*}$ are good approximations to the BCF. At this point, we can write the ODE corresponding to this system of reactions and just modify the association rate constants by $\theta_{l}^{*}$ and $\theta_{t}^{*}$, as shown in S1 Text: Equation 38. Let *K*_*d*_ = (*d*_*l*_/*a*_*l*_)^1/(*N*_*r*_−1)^, *K*_*t*_ = (*d*_*t*_ + *κ*_*t*_)/*a*_*t*_, *β*_*l*_ = (*γ*+ 1)/*d*_*l*_, and *β*_*t*_ = (*γ* + 1)/(*κ*_*t*_ + *d*_*t*_), if $\beta_{l},\beta_{t},\bar{R}/K_{t}⪡1$ (dilution and mRNA degradation is much slower than the rate of ribosome unbinding and *K*_*t*_ is sufficiently large comparer to $\bar{R}$ [29]), then a simple expression for the steady state protein concentration is given by

$$\begin{array}{c} \bar{P}=\kappa_{t}{\bar{m}}_{T}\theta_{t}^{*}\bar{R}/K_{t}\underset{\text{ribosome}\;\text{loading}}{\underset{︸}{\frac{\theta_{l}^{*}{\left(\bar{R}/K_{d}\right)}^{N_{r}-1}}{1+\theta_{l}^{*}{\left(\bar{R}/K_{d}\right)}^{N_{r}-1}}}}, \end{array}$$ (22)

where ${\bar{m}}_{T}$ is given by (19).

In [13] it was estimated that *r*_*m*_ = 20 nm and *r*_*R*_ = 10 nm, which implies that *r*_*m*_/r* ≈ 0.88 and *r*_*R*_/r* ≈ 0.44. Assuming the average distance between ribosomes on an mRNA to be 70 nucleotides [30], for a 700 nucleotide mRNA (e.g., GFP or RFP), then we have *N*_*r*_ = 10. Thus, for these values, $\theta_{l}^{*}\approx 1.56$ and $\theta_{t}^{*}\approx 1.07$. This implies that the forward rate in the reaction of 9 ribosomes binding to an mRNA (*a*_*l*_) is amplified by 56% and the rate at which an additional ribosome binds to this complex (*a*_*t*_) increases by 7% with respect to a well-mixed model. From (22), this would imply up to 67% increase in protein production with respect to a well-mixed model.

Taken together, these results suggest that while a well-mixed ODE model may be sufficient to describe transcription, it is not sufficiently descriptive to capture spatial effects on translation, particularly ribosome loading. In this case, the BCF should be incorporated in the ODE. Additionally, these results are indicative that for other processes in the cell where complexes of similar size as polysomes are formed, then spatial effects will likely be substantial.

#### Gene expression regulation by transcription factors

Regulation of gene expression is often performed by transcription factors (TFs) [1]. A transcription factor can either enhance (for activators) or repress (for repressors) transcription. Spatial affects play an identical role in gene regulation via activators as they do in gene regulation via RNAP (Fig 5), thus we focus on transcriptional repressors. In this section, we model transcription regulation where a repressor P_r_ dimerizes to form dimer c_1_ (e.g., TetR dimerizes before binding to a gene [31]) and then blocks transcription of gene D that produces protein P. The biochemical reactions corresponding to this process are:

$$\begin{array}{c} \emptyset \overset{\alpha }{⟶}P_{r},\;\underset{\text{Case}\;\text{I}}{\underset{︸}{P_{r}+P_{r}\overset{a_{1}}{\underset{d_{1}}{⇌}}c_{1}}},\;\underset{\text{Case}\;\text{II}}{\underset{︸}{c_{1}+D\overset{a_{2}}{\underset{d_{2}}{⇌}}c_{2},}}\;D\overset{\kappa }{⟶}P,\;P_{r}\overset{\gamma_{r}}{⟶}\emptyset ,\;P\overset{\gamma_{p}}{⟶}\emptyset , \end{array}$$ (23)

where *α* is the production rate of P_r_, *a*_1_ (*d*_1_) is the association (dissociation) rate constant to form the c_1_ complex, *a*_2_ (*d*_2_) is the association (dissociation) rate constant to form the c_2_ complex, *κ* is the catalytic rate constant to produce protein P, and *γ*_*r*_ and *γ*_*p*_ are the degradation rate constant of *P*_*r*_ and *P*, respectively. Notice that we have lumped the transcription and translation process to produce P_r_ into one production reaction and similarly for P. From the results of the previous section, we know that $\bar{\alpha }\left(t\right)$ depends on the location where P_r_ is expressed (higher if its coding DNA is near the cell poles than mid-cell) and the size of it’s mRNA (higher for longer mRNAs). Similarly, *κ* depends on the location where P_r_ is expressed and it’s mRNA size. Using the results from the previous section, we can explicitly model these dependences, however, we opt not to do so to solely investigate the role of spatial effects on transcriptional repression. Since the repressor P_r_, freely diffuses, the dimerization reaction belongs to Case I. The gene D is spatially fixed and it is repressed by the freely diffusing c_1_, thus this interaction falls under Case II. We assume that the total concentration of D is conserved, so that *D*_*T*_(*x*) = *D*(*t*, *x*) + *c*_2_(*t*, *x*) and that *D*_*T*_(*x*) is localized at *x* = x*. The reduced ODE model corresponding to (23) obeys

$$\begin{array}{c} \frac{d{\bar{P}}_{r}\left(t\right)}{dt}=\bar{\alpha }\left(t\right)-\gamma_{r}{\bar{P}}_{r}\left(t\right),\;\frac{d{\bar{c}}_{1}\left(t\right)}{dt}=a_{1}\theta_{1}^{*}{\bar{P}}_{r}^{2}\left(t\right)-d_{1}c_{1}\left(t\right)-a_{2}\theta_{2}^{*}{\bar{c}}_{1}\left(t\right)\bar{D}\left(t\right)+d_{2}{\bar{c}}_{2}\left(t\right),\\ \frac{d{\bar{c}}_{2}\left(t\right)}{dt}=a_{2}\theta_{2}^{*}\bar{D}\left(t\right){\bar{c}}_{i}\left(t\right)-d_{2}{\bar{c}}_{2}\left(t\right),\;\bar{D}\left(t\right)=1-{\bar{c}}_{2}\left(t\right),\;\frac{d\bar{P}\left(t\right)}{dt}=\kappa \bar{D}\left(t\right)-\gamma_{p}\bar{P}\left(t\right). \end{array}$$ (24)

Concentration variables were nondimensionalized with respect to the space averaged total DNA ${\bar{D}}_{T}=\int_{0}^{1}D_{T}\left(x\right)dx$. From our main result, the BCF’s are given by

$$\begin{array}{c} \theta_{1}^{*}=\int_{0}^{1}{\hat{v}}_{P_{r}}^{2}\left(x\right)dx=\frac{\int_{0}^{1}v_{P_{r}}^{2}\left(x\right)dx}{{\left[\int_{0}^{1}v_{P_{r}}\left(x\right)dx\right]}^{2}},\;\theta_{2}^{*}={\hat{v}}_{c_{1}}\left(x^{*}\right)=\frac{v_{c_{1}}\left(x^{*}\right)}{\int_{0}^{1}v_{c_{1}}\left(x\right)dx}, \end{array}$$ (25)

where $v_{P_{r}}\left(x\right)=e^{-{\left(r/r^{*}\right)}^{2}\hat{\rho }\left(x\right)}$ and $v_{c_{1}}\left(x\right)=v_{P_{r}}^{2}\left(x\right)$ (recall (10)) are the available volume profiles of P_r_ and c_1_, respectively, and *r* is the radius of gyration of P_r_.

**Fig 6:**
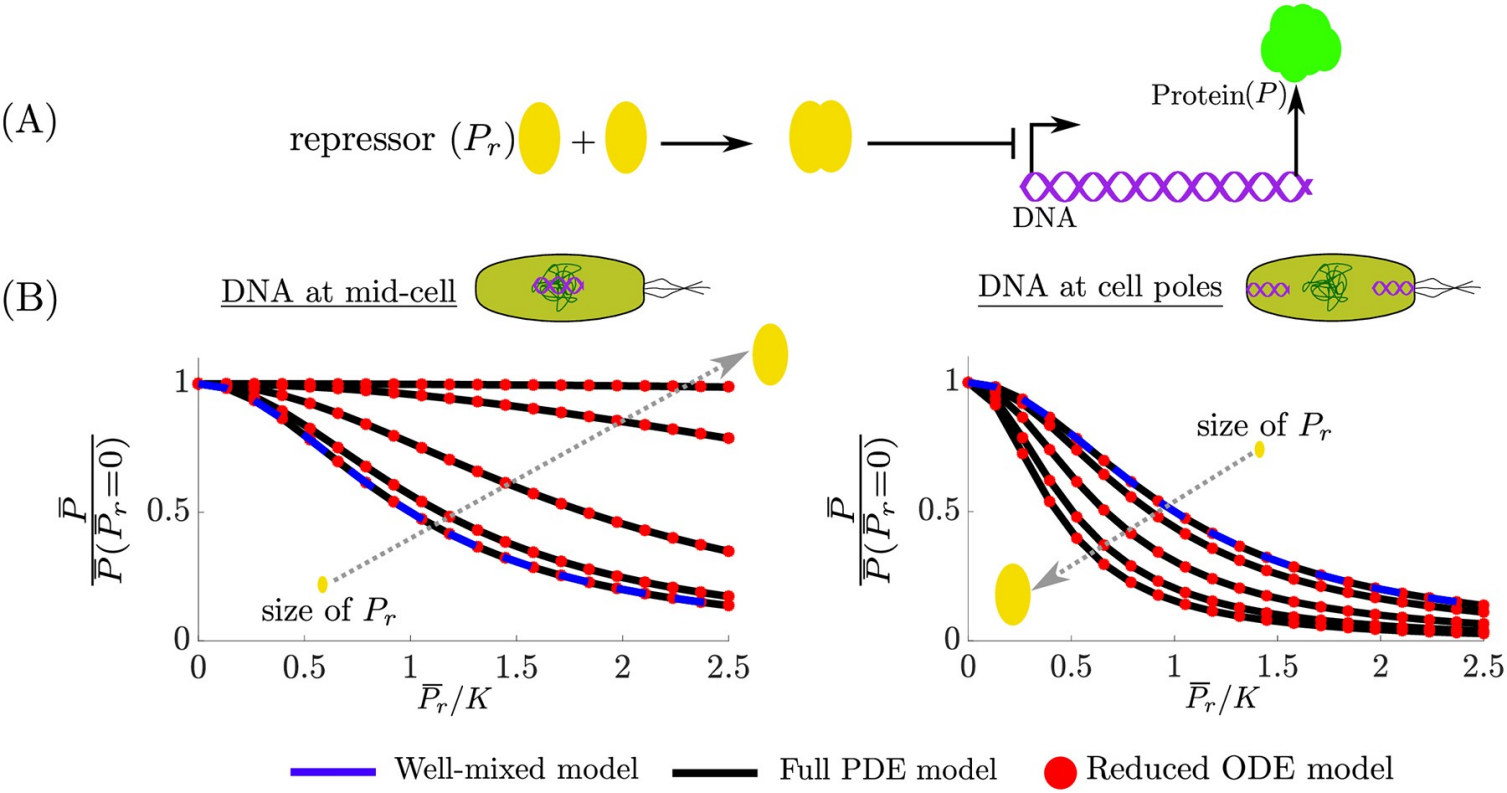
Spatial heterogeneity effects in transcriptional regulation. (A) The repressor P_r_ dimerizes and regulates the production of protein P. (B) The steady state space-averaged concentration per-cell of $\bar{P}$ normalized by its value when ${\bar{P}}_{r}=0$ (26) for the PDE model (S1 Text: Equation 42), the well-mixed model ((24) with $\theta_{1}^{*}=\theta_{2}^{*}=1$), and the reduced ODE model (24) when the DNA is located near mid-cell (x* ≈ 0 in (26)) and when the DNA is located at the cell-poles (x* ≈ 1) for several sizes of P_r_. The parameter values and full simulation details are provided in S1 Text: Section 4.

We now consider the steady state behavior of system (24) by equating the time derivatives to zero. Specifically, we are interested in how the steady state levels of P is affected by the spatial quantities *r*/r* and x*. From setting (24) to steady state, we obtain

$$\begin{array}{c} \bar{P}=\frac{\kappa }{\gamma_{p}}\bar{D},\;\text{where}\;\;\bar{D}=\frac{1}{1+{\left({\bar{P}}_{r}/K\right)}^{2}\theta^{*}},\;\theta^{*}=\theta_{1}^{*}\theta_{2}^{*}={\hat{v}}_{P_{r}}^{2}\left(x^{*}\right), \end{array}$$ (26)

where $K=\sqrt{K_{d,1}K_{d,2}}$ and *K*_*d*,*i*_ = *d*_*i*_/*a*_*i*_ for *i* = 1, 2. From (26) and (25), we observe that θ* contains all the spatial information, which includes the size of P_r_ and the location of the target gene D. If θ* = 1, then the protein concentration would be the same as the well-mixed model. The ratio $K/\sqrt{\theta^{*}}$ can be thought of as an effective disassociation rate constant of the repressor. If D is located near mid-cell (x* ≈ 0 in (25)), then for *r*/r* ≪ 1 we have θ* ≈ 1 (see Fig 4), but as *r*/r* increases, we have that θ* < 1 and asymptotically approaches zero as *r*/r* → ∞. Similarly, if D is located near the cell poles (x* ≈ 1 in (25)), then for *r*/r* ≪ 1 we have θ* ≈ 1 (see Fig 4), but as *r*/r* increases, we have that θ* > 1. Thus, the efficacy of a transcriptional repressor regulating genes in the chromosome (cell-poles) decreases (increases) with TF size. Intuitively, this occurs because as the TF size increases, excluded volume effects will push it out of the chromosome onto the cell-poles (see Remark 1), thus interacting with DNA near the cell-poles more frequently than with DNA near mid-cell. Numerical simulations validate our predictions as shown in Fig 6, where increasing the transcription factor size leads to higher (lower) repression when the target DNA is localized at the cell poles (mid-cell) with respect to a well-mixed model. The simulation results also show agreement between the predictions of the full PDE (S1 Text: Equation 42) and reduced ODE model (24) (as shown explicitly in S1 Text: Fig L). Furthermore, we demonstrate in S1 Text: Fig J, that this agreement extends to the temporal dynamics. Finally, all our results hold independent of the binding and unbinding speeds of the transcription factor dimerizing and of the dimer binding to the DNA (S1 Text: Fig K). In S1 Text: Section 8, we propose an experimental method to test the hypothesis that mid-cell genes are regulated less effectively than pole localized genes.

The reactions in (23) can be easily extended to CRISPRi/dCas9 repression systems [34], where instead of two identical species dimerizing, we have two distinct freely diffusing species bind (dCas9 and guide RNA) to form the complex gRNA-dCas9, which targets a desired DNA sequence. Exploiting the insight gained from analyzing (23), we expect that due to the large size of dCas9 [35] (which is further augmented as it forms a complex with the gRNA), it will regulate pole localized DNA (e.g,. ColE1 plasmid DNA [9]) more efficiently than genes in mid-cell (e.g,. chromosomally integrated) and thus spatial effects are expected to be more significant when using CRISPRi/dCas9 in genetic circuit design. Specifically, based on approximate values found in the literature, we estimated that θ* ≈ 1 for a transcription factor, while θ* can range between 0.9 and 1.1 for dCas9-enabled repression. This indicates that a well-mixed model is appropriate for modeling transcription factor-enabled repression of gene expression but may not be sufficient to capture effects of spatial heterogeneity arising with larger repressing complexes such as with dCas9/gRNA (see S1 Text: Section 4 for details).

#### Genetic oscillator

**Fig 7:**
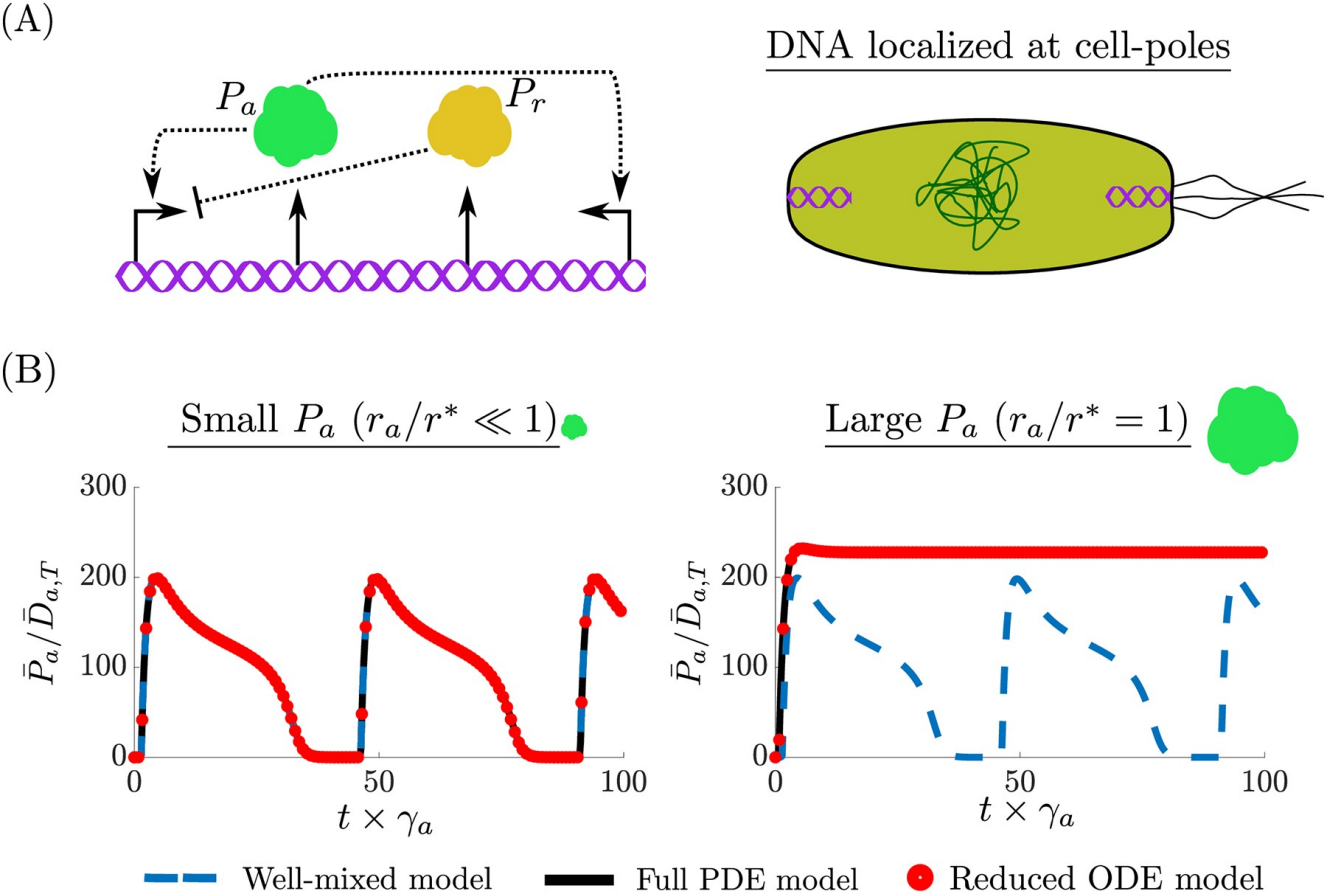
Spatial effects on the dynamics of genetic circuits. (A) The activator-repressor clock where P_r_ represses P_a_ and P_a_ activates itself and P_r_. Both proteins are expressed from the same cell-pole localized plasmid. (B) The temporal evolution of P_a_ is given for the full-PDE model (S1 Text: Equation 44), the reduced ODE model (28), and the well-mixed model (same as (28) with $\theta_{A,1}^{*}=\theta_{A,2}^{*}=\theta_{R}^{*}=1$). When P_a_ is small (*r*_*a*_/r* ≪ 1), all three models predict sustained oscillations. When P_a_ is large (*r*_*a*_/r* = 1), the full-PDE model and the reduced ODE model predict the oscillations will cease. For both simulations $r_{r}/r^{*}⪡1\Rightarrow \theta_{R}^{*}\approx 1$. The full simulation details and parameter values are given in S1 Text: Section 5.

As a final example, we consider the repressor-activator clock genetic circuit designed in [32] and shown in Fig 7A. This circuit produces sustained oscillations if tuned within an appropriate parameter range [1, 33]. The circuit consists of two proteins P_a_ and P_r_. Protein P_a_, is an activator which dimerizes to form P_a,2_ and then binds to its own gene D_a_ to form complex c_a,1_ to initiate transcription. The dimer P_a,2_ also binds to the gene D_r_, which transcribes P_r_ to form complex c_a,2_ and initiates transcription. Protein P_r_, dimerizes to form P_r,2_ and then represses P_a_ by binding to D_a_ to form complex c_r_. The biochemical reactions corresponding to this circuit are:

$$\begin{array}{cc} & \underset{\text{Case}\;\text{I},\;P_{a}\;\text{diffuses}}{\underset{︸}{P_{a}+P_{a}\overset{a_{1}}{\underset{d_{1}}{⇌}}P_{a,2},}}\;\underset{\text{Case}\;\text{II},\;P_{r}\;\text{diffuses}}{\underset{︸}{P_{r}+P_{r}\overset{a_{2}}{\underset{d_{2}}{⇌}}P_{r,2},}}\;\underset{\text{Case}\;\text{II},\;P_{a,2}\;\text{diffuses}\;\text{and}\;D_{r}\;\text{fixed}}{\underset{︸}{P_{a,2}+D_{r}\overset{a_{4}}{\underset{d_{4}}{⇌}}c_{a,2},}}\\ & \underset{\text{Case}\;\text{III},\;P_{a,2}\;\text{and}\;P_{r,2}\;\text{diffuses}\;\text{and}\;D_{a}\;\text{fixed}}{\underset{︸}{P_{a,2}+D_{a}\overset{a_{3}}{\underset{d_{3}}{⇌}}c_{a,1},\;P_{r,2}+D_{a}\overset{a_{5}}{\underset{d_{5}}{⇌}}c_{r},}}\;D_{a}\overset{\kappa_{1}}{⟶}P_{a},\\ & D_{r}\overset{\kappa_{2}}{⟶}P_{r},\;P_{a}\overset{\gamma_{a}}{⟶}\emptyset ,\;P_{r}\overset{\gamma_{r}}{⟶}\emptyset ,\;c_{a,1}\overset{\kappa_{3}}{⟶}P_{a},\;c_{a,2}\overset{\kappa_{4}}{⟶}P_{r}, \end{array}$$ (27)

where *a*_*i*_ (*d*_*i*_) for *i* = 1, …, 5 are association (dissociation) rate constants, *γ*_*a*_ (*γ*_*r*_) is the degradation rate constant of P_a_ (P_r_) *κ*_1_ (*κ*_2_) is the basal rate at which gene D_a_ (D_r_) is transcribed, and *κ*_3_ (*κ*_4_) is the rate at which the DNA-transcription-factor complexes are transcribed for D_a_ (D_r_). We assume that the total concentration of D_a_ is conserved, so that *D*_*a*,*T*_(*x*) = *D*_*a*_(*t*, *x*) + *c*_*a*,1_(*t*, *x*) + *c*_*r*_(*t*, *x*) and that D_a,T_ is localized at $x=x_{a}^{*}$. Similarly, we assume that the total concentration of D_r_ is conserved, so that *D*_*r*,*T*_(*x*) = *D*_*r*_(*t*, *x*)+ *c*_*a*,2_(*t*, *x*) and that D_r,T_ is localized at $x=x_{r}^{*}$. The reduced ODE model corresponding to (27) is given by:

$$\begin{array}{cc} \frac{d{\bar{P}}_{a}\left(t\right)}{dt} & =\kappa_{1}{\bar{D}}_{a}\left(t\right)+\kappa_{3}{\bar{c}}_{a,1}\left(t\right)-\gamma_{a}{\bar{P}}_{a}\left(t\right),\;\frac{d{\bar{P}}_{r}\left(t\right)}{dt}=\kappa_{2}{\bar{D}}_{r}\left(t\right)+\kappa_{4}{\bar{c}}_{a,2}\left(t\right)-\gamma_{r}{\bar{P}}_{r}\left(t\right),\\ \frac{d{\bar{P}}_{a,2}\left(t\right)}{dt} & =a_{1}\theta_{1}^{*}{\bar{P}}_{a}^{2}\left(t\right)-d_{1}{\bar{P}}_{a,2}\left(t\right)\\ & -a_{3}\theta_{3}^{*}{\bar{P}}_{a,2}\left(t\right){\bar{D}}_{a}\left(t\right)+d_{3}{\bar{c}}_{a,1}\left(t\right)-a_{4}\theta_{4}^{*}{\bar{P}}_{a,2}\left(t\right){\bar{D}}_{r}\left(t\right)+d_{4}{\bar{c}}_{a,2}\left(t\right),\\ \frac{d{\bar{P}}_{r,2}\left(t\right)}{dt} & =a_{2}\theta_{2}^{*}{\bar{P}}_{r}^{2}\left(t\right)-d_{2}{\bar{P}}_{r,2}\left(t\right)-a_{5}\theta_{5}^{*}{\bar{P}}_{r,2}\left(t\right){\bar{D}}_{a}\left(t\right)+d_{5}{\bar{c}}_{r}\left(t\right),\\ \frac{d{\bar{c}}_{a,1}\left(t\right)}{dt} & =a_{3}\theta_{3}^{*}{\bar{P}}_{a,2}\left(t\right){\bar{D}}_{a}\left(t\right)-d_{3}{\bar{c}}_{a,1}\left(t\right),\\ \frac{d{\bar{c}}_{a,2}\left(t\right)}{dt} & =a_{4}\theta_{4}^{*}{\bar{P}}_{a,2}\left(t\right){\bar{D}}_{r}\left(t\right)-d_{4}{\bar{c}}_{a,2}\left(t\right),\;\frac{d{\bar{c}}_{r}\left(t\right)}{dt}=a_{5}\theta_{5}^{*}{\bar{P}}_{r,2}\left(t\right){\bar{D}}_{a}\left(t\right)-d_{5}{\bar{c}}_{r}\left(t\right),\\ {\bar{D}}_{a}\left(t\right) & =1-{\bar{c}}_{a,1}\left(t\right)-{\bar{c}}_{r}\left(t\right),\;{\bar{D}}_{r}\left(t\right)={\bar{D}}_{r,T}-{\bar{c}}_{a,2}\left(t\right). \end{array}$$ (28)

Concentration variables were nondimensionalized with respect to the space averaged total DNA ${\bar{D}}_{a,T}=\int_{0}^{1}D_{a,T}\left(x\right)dx$. Applying our main result, the BCF’s are given by

$$\begin{array}{c} \theta_{1}^{*}=\int_{0}^{1}{\hat{v}}_{P_{a}}^{2}\left(x\right)dx,\;\theta_{2}^{*}=\int_{0}^{1}{\hat{v}}_{P_{r}}^{2}\left(x\right)dx,\; \end{array}$$

$$\begin{array}{c} \theta_{3}^{*}={\hat{v}}_{P_{a,2}}\left(x_{a}^{*}\right),\;\theta_{4}^{*}={\hat{v}}_{P_{a,2}}\left(x_{r}^{*}\right),\;\theta_{5}^{*}={\hat{v}}_{P_{r,2}}\left(x_{a}^{*}\right), \end{array}$$

where ${\hat{v}}_{P_{a}}\left(x\right)$, ${\hat{v}}_{P_{r}}\left(x\right)$, ${\hat{v}}_{P_{a,2}}\left(x\right)$, and ${\hat{v}}_{P_{r,2}}\left(x\right)$ are the normalized available volume profiles (i.e,. ${\hat{v}}_{P_{a}}\left(x\right)=v_{P_{a}}\left(x\right))/\int_{0}^{1}v_{P_{a}}\left(x\right)dx$) of P_a_, P_r_, P_a,2_, and P_r,2_, respectively. From (10), notice that $v_{P_{a,2}}\left(x\right)=v_{P_{a}}^{2}\left(x\right)$ and $v_{P_{r,2}}\left(x\right)=v_{P_{r}}^{2}\left(x\right)$. The available volume profiles are given by

$$\begin{array}{c} v_{P_{a}}\left(x\right)=e^{-{\left(r_{a}/r^{*}\right)}^{2}\hat{\rho }\left(x\right)},\;v_{P_{r}}\left(x\right)=e^{-{\left(r_{r}/r^{*}\right)}^{2}\hat{\rho }\left(x\right)}, \end{array}$$ (29)

where *r*_*a*_ and *r*_*r*_ are the radius of gyration of P_a_ and P_r_, respectively. Approximating ${\bar{P}}_{a,2}\left(t\right)$, ${\bar{P}}_{r,2}\left(t\right)$, ${\bar{c}}_{a,1}\left(t\right)$, ${\bar{c}}_{a,2}\left(t\right)$ and ${\bar{c}}_{r}\left(t\right)$ at their quasi-steady state (since *d*_*i*_ ≫ *γ*_*a*_, *γ*_*r*_ for *i* = 1, …, 5, [1]), we obtain

$$\begin{array}{c} {\bar{P}}_{a,2}\left(t\right)=\frac{a_{1}\theta_{1}}{d_{1}}{\bar{P}}_{a}^{2}\left(t\right),\;{\bar{P}}_{r,2}\left(t\right)=\frac{a_{2}\theta_{2}}{d_{2}}{\bar{P}}_{r}^{2}\left(t\right), \end{array}$$

$$\begin{array}{c} {\bar{c}}_{a,1}\left(t\right)=\frac{a_{3}\theta_{3}}{d_{3}}{\bar{P}}_{a,2}\left(t\right){\bar{D}}_{a}\left(t\right),\;{\bar{c}}_{a,2}\left(t\right)=\frac{a_{4}\theta_{4}}{d_{4}}{\bar{P}}_{a,2}\left(t\right){\bar{D}}_{r}\left(t\right),\;{\bar{c}}_{r}\left(t\right)=\frac{a_{5}\theta_{5}}{d_{5}}{\bar{P}}_{r,2}\left(t\right){\bar{D}}_{a}\left(t\right), \end{array}$$

and, therefore, we can further reduce (28) to

$$\begin{array}{c} \frac{d{\bar{P}}_{a}}{dt}=\frac{\alpha_{0,A}+\alpha_{A}{\left(\frac{{\bar{P}}_{a}}{K_{d,1}}\right)}^{2}}{1+{\left(\frac{{\bar{P}}_{a}}{K_{d,1}}\right)}^{2}+{\left(\frac{{\bar{P}}_{r}}{K_{d,2}}\right)}^{2}}-\gamma_{A}{\bar{P}}_{a},\;\frac{d{\bar{P}}_{r}}{dt}=\frac{\alpha_{0,R}+\alpha_{R}{\left(\frac{{\bar{P}}_{a}}{K_{d,3}}\right)}^{2}}{1+{\left(\frac{{\bar{P}}_{a}}{K_{d,3}}\right)}^{2}}-\gamma_{R}{\bar{P}}_{r} \end{array}$$ (30)

where $\alpha_{0,A}=\kappa_{1}{\bar{D}}_{a,T}$ ($\alpha_{0,R}=\kappa_{2}{\bar{D}}_{r,T}$) is the basal production rate of P_a_ (P_r_), *α*_*A*_ = *κ*_3_ ($\alpha_{R}=\kappa_{4}{\bar{D}}_{r,T}$) is the additional production rate of P_a_ (P_r_) due to activation from P_a_, and

$$\begin{array}{c} K_{d,1}=\frac{d_{1}d_{3}}{\theta_{A,1}^{*}a_{1}a_{3}},\;K_{d,2}=\frac{d_{2}d_{5}}{\theta_{R}^{*}a_{2}a_{5}},\;K_{d,3}=\frac{d_{1}d_{4}}{\theta_{A,2}^{*}a_{1}a_{4}}, \end{array}$$ (31)

$$\begin{array}{c} \theta_{A,1}^{*}=\theta_{1}^{*}\theta_{3}^{*}={\hat{v}}_{P_{a}}\left(x_{a}^{*}\right),\;\theta_{R}^{*}=\theta_{2}^{*}\theta_{5}^{*}={\hat{v}}_{P_{r}}\left(x_{a}^{*}\right),\;\theta_{A,2}^{*}=\theta_{1}^{*}\theta_{4}^{*}={\hat{v}}_{P_{a}}\left(x_{r}^{*}\right). \end{array}$$ (32)

The form of the dynamics given by (30) was theoretically analyzed in [1, 33], and it was shown that the values of *K*_*d*,*i*_ for *i* = 1, 2, 3, were critical in determining whether sustained oscillations occur. From (31), these parameters depend on (32) and thus on the size of P_a_ and P_r_ through the available volume profiles (29) and the location of D_a_ and D_r_ ($x_{a}^{*}$ and $x_{r}^{*}$). Numerical simulations demonstrate how these spatial parameters affect circuit behavior. In our simulation setup, the parameters are chosen such that the well-mixed model ((28) with $\theta_{R}^{*}=\theta_{A,1}^{*}=\theta_{A,2}^{*}=1$) oscillates, the DNA of P_a_ and P_r_ are localized at the cell poles and have the same copy number (i.e., $x_{a}^{*}=x_{r}^{*}$ and ${\bar{D}}_{r,T}={\bar{D}}_{a,T}=1$), the size of P_r_ is chosen to be small *r*_*r*_/r* ≪ 1 (thus $\theta_{R}^{*}\approx 1$), and the size of P_a_ is varied (thus varying $\theta_{A,1}^{*}$ and $\theta_{A,2}^{*}$). Since D_a_ is localized at the cell poles, it implies $x_{a}^{*}\approx 1$ and from (32), we observe that if $r_{a}/r^{*}⪡1\Rightarrow \theta_{A,1}^{*}\approx \theta_{A,2}^{*}\approx 1$ and $\theta_{A,1}^{*},\theta_{A,2}^{*}$ increase as *r*_*a*_/r* increases. The results of these simulations are shown in Fig 7. When *r*_*a*_/r* ≪ 1, the full PDE model (S1 Text: Equation 44), the reduced ODE model (28), and the well-mixed model are all in agreement and sustained oscillations are observed. By contrast, when *r*_*a*_/r* = 1, the PDE and reduced model (which are in agreement with each other as explicitly shown in S1 Text: Fig N) predict that sustained oscillations will no longer occur. Furthermore, in S1 Text: Figure M, we demonstrate that indeed as the size of P_a_ increases it is excluded from the chromosome onto the cell poles while the spatial profile of P_r_ is homogeneously distributed throughout the cell since *r*_*r*_/r* ≪ 1 (Remark 1).

## Discussion

We derived a reduced order ODE model of genetic circuits with the same dimension as traditional ODE well-mixed models; yet, it captures effects of spatial heterogeneity within bacterial cells (12). In particular, our reduced model is the same as a well-mixed model where all the association rate constants are multiplied by the binding correction factor (BCF). This factor depends on the size and location (if fixed in space) of the reacting species, according to an analytical formula that we derived from first principles (13) and its value can be estimated experimentally through simple procedures (S1 Text: Section 7). We have mathematically demonstrated that this reduced order model is a good approximation of the space-averaged dynamics resulting from a reaction-diffusion PDE model under the assumption of fast diffusion. It can therefore be used in place of PDE models, providing substantial advantages for both simulation and mathematical analysis.

We applied this model to analyze the effects of spatial heterogeneity on core biological processes and genetic circuits. Specifically, motivated by the fact that DNA, ribosomes, and mRNA have been shown to localize within the cell [4, 5, 14], we analyzed the transcription and translation processes. We determined that mRNA levels are lower (higher) when the gene is localized near the mid-cell (cell poles). We also showed that when the target gene of a transcriptional repressor is near mid-cell (cell poles) the effective repression is lower (higher) with respect to that of the well-mixed model. This discrepancy is amplified as the size of the transcription factor increases. The extent of these spatial effects depends on how different the value of the BCF is from unity. Based on parameters found in the literature, we determined that for the processes of transcription and its regulation the BCF should be close to unity and hence a well-mixed ODE model should be sufficient. However, in situations where the nucleoid is highly compacted (from overexpressing mRNA [13] or translational inhibition [36]), we expect that the available volume profile (2) approaches small values and, as a consequence, the value of the BCF can substantially deviate from unity (13).

Our results provide additional interpretations of well-known biological phenomena. For example, it has been shown that the expression rate of chromosomal genes depends on the locus where the gene is inserted [37]; that the nucleoid dynamically changes shape to control gene expression and transcription regulation [4, 38] (e.g., see S1 Text: Section 9, for how a time varying chromosome density modulates the BCF); and that coregulation and coexpression among genes depends on their spatial distance [39]. For a fixed amount of mRNA, we showed that spatial heterogeneity leads to higher translation rates since both mRNA and ribosome are pushed out of the chromosome into a smaller region near the cell poles, which results in larger effective binding affinity. How larger, it depends on the value of the BCF. For a polysome with 10 translating ribosomes, the value of the BCF can deviate from unity by 56% in the ribosome loading step and by 7% in the peptide release step. These estimates are believed to be conservative since we did not account for the exclusion effects from the peptide chains attached to the translating ribosome, which will result in even more pronounced spatial effects. Therefore, a well-mixed model may not be sufficient to capture the effects of spatial heterogeneity on translation.

Our modeling framework can be easily extended to other aspects of gene expression. For example, we may consider co-transcriptional translation [40]. In this case, as a result of translation being localized at the gene location, the effective ribosome binding site strength will also depend on gene location through the BCF. We may also consider the role of spatial heterogeneity on orthogonal translational machinery [41]. From our models, we predict that one can tune the rate at which orthogonal ribosomes are formed by creating larger synthetic 16S rRNA. Furthermore, once the production of orthogonal ribosomes is placed in a feedback form to decouple genetic circuits [41], our framework suggests that the feedback efficiency may depend on the spatial location of the synthetic 16S rRNA gene. The value of the parameter r*, whose squared value is inversely proportional to the average chromosome density (2), is critical in determining the extent of spatial effects. In this study we indirectly estimated a value of r* based on [13]. However, a more comprehensive study should be conducted to estimate r* for several contexts (S1 Text: Section 7), or equivalently to estimate extent of excluded volume effects, which may easily be performed via superresolution imaging [14].

In summary, this paper provides a general and convenient modeling framework to account for DNA localization and excluded volume effects on intracellular species dynamics. While other phenomena contributing to intracellular spatial heterogeneity, such as crowding [42], sliding, hopping, and dimensionality [17], exist, this is a first step towards creating a general framework to modify current models to capture spatial information. Our model can be used both as an analysis and a design tool for genetic circuits, in which variables such as gene location and regulator size may be considered as additional design parameters.

## Supporting information

S1 Text Supporting information file with mathematical proofs, detailed analysis of examples, generalization and extension of the results and additional simulations. (PDF)
