# Supplementary material for: Effects of spatial heterogeneity on bacterial genetic circuits
Source: PLoS Comput Biol. 2020 Sep 14;16(9):e1008159. doi: 10.1371/journal.pcbi.1008159 (PMC7515207; doi:10.1371/journal.pcbi.1008159)
Supplement: S1 Text — (PDF) [file pcbi.1008159.s001.pdf]

# Supplementary information to "Effects of spatial heterogeneity on bacterial genetic circuits"

Carlos Barajas[1] and Domitilla Del Vecchio[1*]

[1] Department of Mechanical Engineering, Massachusetts Institute of Technology, Cambridge, Massachusetts

[*] Correspondence: ddv@mit.edu

# 1 Time Scale Separation Proofs

## 1.1 Preliminaries:

*Notation*: Let $\boldsymbol{z} = [z_1, \dots, z_n]^T \in \mathbb{R}^n$ (where superscript $T$ denotes the transpose operation) and the $j$-th component of $\boldsymbol{z}$ is denoted by $\boldsymbol{z}^j$. A vector of zeros is denoted as $\mathbf{0}_n = [0, \dots, 0]^T \in \mathbb{R}^n$ and we use $\boldsymbol{A} = \text{diag}(\boldsymbol{v}) \in \mathbb{R}^{n \times n}$ to refer to a square matrix with all zeros in the off-diagonals and diagonal elements specified by the vector $\boldsymbol{v}$. The $(j,k)$-th element of matrix $\boldsymbol{A}$ is denoted by $\boldsymbol{A}^{j,k}$ and $\boldsymbol{I}_{n,n}$ is the identity matrix acting on $\mathbb{R}^n$. Let $\mathbb{R}^n_+ = \{[z_1, \dots, z_n]^T : z_i > 0, i = 1, \dots, n\}$ denote the positive orthant of $\mathbb{R}^n$. For a Hilbert space $H$ with inner product $\langle \cdot, \cdot \rangle_H$, we denote the norm as $|| \cdot ||_H = \sqrt{\langle \cdot, \cdot \rangle_H}$. Finally, let overbars denote spatial integration $\bar{u} := \int_0^1 u(x) dx$.

**Definition** 1. (Linear Differential Operator) Let $v(x) : [0,1] \to \mathbb{R}_+$ be a smooth function, and consider the following linear differential operator:

$$\mathcal{L}_v(y) := \frac{d}{dx}\left[v^2(x)\frac{d}{dx}\left[\frac{y(x)}{v(x)}\right]\right], \tag{1}$$

with domain $D(\mathcal{L}_v) = \{y \in L^2(0,1) : v^2 \frac{d}{dx}\left[\frac{y}{v}\right]\big|_{x=0,1} = 0\}$:

Next, we introduce the Hilbert space $L^2_v(0,1)$ that we use for our analysis. This space is isomorphic to $L^2(0,1)$ however, the operator (1) is self-adjoint with respect to the inner product in $L^2_v(0,1)$.

**Definition** 2. (Weighted $L^2(0,1)$ space) For smooth $v : [0,1] \to \mathbb{R}_+$ we denote $L^2_v(0,1)$ as a weighted space of the square integrable functions such that $f \in L^2_v(0,1)$ if and only if $: \frac{f}{\sqrt{v(x)}} \in L^2(0,1)$. The inner-product is defined as $\langle f, g \rangle_v := \int_0^1 \frac{f(x)g(x)}{\hat{v}} dx$, for $f, g \in L^2_v(0,1)$ and $\hat{v}(x) = \frac{v(x)}{\bar{v}}$. Furthermore, let $\boldsymbol{v} = [v_1(x), \dots, v_n(x)]^T$, where $v_i(x) : [0,1] \to \mathbb{R}_+$ is smooth, then $\boldsymbol{z} \in L^2_{\boldsymbol{v}}((0,1), \mathbb{R}^n)$ if $\boldsymbol{z}^i \in L^2_{v_i}(0,1)$ for $i = 1, \dots, n$, and the inner product in this space is defined as $\langle \boldsymbol{f}, \boldsymbol{g} \rangle_{\boldsymbol{v}} := \int_0^1 \boldsymbol{f}^T(x)[\text{diag}(\hat{\boldsymbol{v}})]^{-1}\boldsymbol{g}(x)dx$, for $\boldsymbol{f}, \boldsymbol{g} \in L^2_{\boldsymbol{v}}((0,1), \mathbb{R}^n)$, where $\hat{\boldsymbol{v}}^i(x) = \frac{\boldsymbol{v}^i(x)}{\bar{\boldsymbol{v}}^i}$.

*Remark* 1. (Norm equivalence between $L^2$ and $L^2_v$) For smooth $v : [0,1] \to \mathbb{R}_+$, let $\hat{v}(x) = v(x)/\bar{v}$, $\hat{v}_* = \min_{x \in [0,1]} \hat{v}(x)$ and $\hat{v}^* = \max_{x \in [0,1]} \hat{v}(x)$, the norms defined on $L^2_v(0,1)$ and $L^2(0,1)$ are related by

$$\frac{1}{\sqrt{\hat{v}^*}}||y||_{L^2} \leq ||y||_{L^2_v} \leq \frac{1}{\sqrt{\hat{v}_*}}||y||_{L^2},$$

for any $y \in L^2_v(0,1)$. Thus, when performing convergence analysis we may use the norms defined in $L^2_v(0,1)$ and $L^2(0,1)$ interchangeably.

**Lemma** 1. *(Negative semi-definite and self-adjoint Operator) For smooth $v : [0,1] \to \mathbb{R}_+$, let $\mathcal{L}_v$ be as in Definition 1. This operator has the following properties:*

*I Has countably many, real, and distinct eigenvalues such that $\lambda_1 > \dots > \lambda_n > \dots$ and $\lim_{n \to \infty} \lambda_n = -\infty$*

*II The set of corresponding eigenfunctions $\{\psi_i(x)\}$ form a complete orthonormal basis for $L^2_v(0,1)$ (Definition 2)*

*III* $\lambda_1 = 0$ *and* $\psi_1 = \hat{v}(x) = \frac{v(x)}{\bar{v}}$

*Proof:* The proof of (I) and (II) follow from Sturm-Liouville theory [1]. To prove (III), we take the weighted inner product of both side of $\mathcal{L}_v\psi_i = \lambda_i\psi_i$ with $\psi_i$, we use orthonormality, use integration by parts, and apply the boundary conditions:

$$\begin{aligned} \lambda_i &= \langle \psi_i, \mathcal{L}_v(\psi_i)\rangle_v \\ &= \int_0^1 \frac{\psi_i}{\hat{v}}\frac{d}{dx}\Big(v^2\frac{d}{dx}(\frac{\psi_i}{v})\Big)dx \\ &= \Big[\frac{\psi_i}{\hat{v}}v^2\frac{d}{dx}(\frac{\psi_i}{v})\Big]_{x=0,1}^{\nearrow 0} - \int_0^1 \bar{v}\Big(v\frac{d}{dx}(\frac{\psi_i}{v})\Big)^2 dx \\ &\leq 0. \end{aligned} \tag{2}$$

The maximum of (2) is achieved ($\lambda_1 = 0$) for $\psi_1 = \hat{v}(x)$, since substituting $\hat{v}(x)$ directly into $\mathcal{L}_v$, one observes that $\mathcal{L}_v(\hat{v}(x)) = 0$ and we have that $||\psi_1||_{L_v^2} = \sqrt{\langle\psi_1,\psi_1\rangle_v} = 1$, therefore $\lambda_i < 0, \forall i > 1$. ■

The following will introduce the notion of contracting dynamical systems. A dynamical system is said to be contracting within an open and connected subspace of the state space, if all trajectories starting within this region converge exponentially to each other. We provide sufficient conditions to guarantee that a dynamical system is contracting, and finally show that contracting systems have a particular robustness property. The robustness property will be exploited several times in our analysis to perform our model reduction.

**Theorem** 1. *Let $\dot{\boldsymbol{z}} = \boldsymbol{f}(t,\boldsymbol{z})$ be a dynamical system in the Hilbert space $H = \mathbb{R}^n$ where $\boldsymbol{f}$ is a smooth nonlinear function. A dynamical system is said to be contracting within an open and connected subspace of the state space $\chi \subseteq H$, if all trajectories starting within this region converge exponentially to each other. A sufficient condition for a system to be contracting in $H$ is the existence a uniformly positive definite matrix $\boldsymbol{P}(t,\boldsymbol{z}) \in \mathbb{R}^{n\times n}$ and constant $\lambda > 0$ such that*

$$\frac{1}{2}\big[\frac{\partial \boldsymbol{f}}{\partial \boldsymbol{z}}^T\boldsymbol{P} + \boldsymbol{P}\frac{\partial \boldsymbol{f}}{\partial \boldsymbol{z}} + \dot{\boldsymbol{P}}\big] \leq -\xi\boldsymbol{P}, \forall \boldsymbol{z} \in \chi, \forall t \geq 0 \tag{3}$$

*where $\xi$ is the contraction rate of the system and $\dot{\boldsymbol{P}}$ is the total time derivative of $\boldsymbol{P}$.*

*Proof:* See Theorem 2 in [2]. ■

**Lemma** 2. *(Hierarchies of contracting systems) Let*

$$\frac{d\boldsymbol{z}(t)}{dt} = \frac{d}{dt}\begin{bmatrix}\boldsymbol{z}_1(t)\\ \boldsymbol{z}_2(t)\end{bmatrix} = \begin{bmatrix}\boldsymbol{f}_1(t,\boldsymbol{z}_1)\\ \boldsymbol{f}_2(t,\boldsymbol{z}_1,\boldsymbol{z}_2)\end{bmatrix} \tag{4}$$

*be dynamical systems in the Hilbert space $H = \mathbb{R}^{n+m}$ where $\boldsymbol{f}_1 : [0,\infty)\times\mathbb{R}^n \to \mathbb{R}^n$ and $\boldsymbol{f}_2 : [0,\infty)\times\mathbb{R}^n\times\mathbb{R}^m \to \mathbb{R}^m$ are smooth nonlinear functions. Then, sufficient conditions for (4) to be contracting in $\chi = \chi_1 \oplus \chi_2$, where $\chi_1$ and $\chi_2$ are open connected subspaces of $\mathbb{R}^n$ and $\mathbb{R}^m$, respectively, are*

- *I* $\frac{\partial \boldsymbol{f}_1}{\partial \boldsymbol{z}_1}$ *satisfies (3) in $\chi_1$ for some $\boldsymbol{P}_1 \in \mathbb{R}^{n\times n}$ such that $\boldsymbol{P}_1 = \boldsymbol{P}_1^T > 0$*
- *II* $\frac{\partial \boldsymbol{f}_2}{\partial \boldsymbol{z}_2}$ *satisfies (3) in $\chi_2$ for some $\boldsymbol{P}_2 \in \mathbb{R}^{m\times m}$ such that $\boldsymbol{P}_2 = \boldsymbol{P}_2^T > 0$*
- *III* $\frac{\partial \boldsymbol{f}_2}{\partial \boldsymbol{z}_1}$ *is uniformly bounded for all $t \geq 0$, $\boldsymbol{z}_1 \in \chi_1$, and $\boldsymbol{z}_2 \in \chi_2$*

*Proof:* See hierarchal structures in [2] and applied in [3] ■

**Lemma** 3. *(Robustness property of contracting systems) Assume that $\dot{\boldsymbol{z}} = \boldsymbol{f}(t,\boldsymbol{z})$ satisfies the conditions of Theorem 1, for some $\boldsymbol{P}(t,\boldsymbol{z}) = \boldsymbol{P}(t,\boldsymbol{z})^T > 0$ in a region $\chi \subseteq H$, and thus it is contracting with some contraction rate $\xi$. Furthermore, assume that there exists a positive constant $\lambda^*$ ( $\lambda_*$) that upper (lower) bounds the maximum (minimum) eigenvalue of $\boldsymbol{P}$ for all $t \geq 0$. Consider the "perturbed" system $\dot{\boldsymbol{z}}_{\boldsymbol{p}} = \boldsymbol{f}(t,\boldsymbol{z}_p)+\boldsymbol{d}(t,\boldsymbol{z}_p)$, and suppose there exists $L_1, L_2, \zeta, \epsilon > 0$ such that $\zeta/\epsilon > \xi$ and $||\boldsymbol{d}(t,\boldsymbol{z}_p)||_H \leq L_1e^{-\zeta/\epsilon t} + L_2\epsilon$ for all $t \geq 0$ and $\boldsymbol{z}_p \in \chi$. Then, there exists $L^*, \epsilon^* > 0$ such that for all $\boldsymbol{z}(0), \boldsymbol{z}_p(0) \in \chi$ and $0 < \epsilon < \epsilon^*$, the solutions $\boldsymbol{z}_p(t)$ and $\boldsymbol{z}(t)$ satisfy*

$$||\boldsymbol{z}_p(t) - \boldsymbol{z}(t)||_H \leq \sqrt{\frac{\lambda^*}{\lambda_*}}\Big(||\boldsymbol{z}_p(0) - \boldsymbol{z}(0)||_H e^{-\xi t} + L^*\epsilon\Big), \quad \forall t \geq 0.$$

*Proof:* This follows from the result of Seciton 3.7 Result vii in [2]. Let $R_z(0)$ be the length of the straight path connecting $\boldsymbol{z}_p(0)$ and $\boldsymbol{z}(0)$, that is, $R_z(0) = \int_{\boldsymbol{z}(0)}^{\boldsymbol{z}_p(0)} ||\delta z(0)||_H = ||\boldsymbol{z}_p(0) - \boldsymbol{z}(0)||_H$, where $\delta z$ is the virtual displacement (see [2] for details. Each point in the straight path connecting $\boldsymbol{z}_p(0)$ and $\boldsymbol{z}(0)$ evolves in time and we denote the length of the path connecting these points be given by $R_z(t)$. Precisely, $R_z(t) = \int_{\boldsymbol{z}}^{\boldsymbol{z}_p} ||\delta z(t)||_H$. Notice that

$$||\boldsymbol{z}_p(t) - \boldsymbol{z}(t)||_H \leq R_z(t), \tag{5}$$

since the straight line path is the shortest path between the trajectories (they would be equal if the initial straight line segment remained a line for all time, but this is only the case for constant vector fields). Let $R(t) = \int_{\boldsymbol{z}}^{\boldsymbol{z}_p} ||P^{1/2}\delta z||_H$. Notice that

$$\sqrt{\lambda_*} R_z(t) \leq R(t) \leq \sqrt{\lambda^*} R_z(t). \tag{6}$$

From Equation 15 in [2], we have that $\dot{R} + \xi R \leq ||\boldsymbol{P}^{1/2} d||_H$ and thus

$$R(t) \leq R(0)e^{-\xi t} + \sqrt{\lambda^*} \int_0^t ||\boldsymbol{d}(\tau)||_H e^{-\xi(t-\tau)d\tau}.$$

From (6), we have that

$$\sqrt{\lambda_*} R_z(t) \leq \sqrt{\lambda^*} R_z(0)e^{-\xi t} + \sqrt{\lambda^*} \int_0^t ||\boldsymbol{d}(\tau)||_H e^{-\xi(t-\tau)d\tau}.$$

Finally, by (5), this implies that

$$\begin{aligned} ||\boldsymbol{z}_p(t) - \boldsymbol{z}(t)||_H &\leq \sqrt{\frac{\lambda^*}{\lambda_*}} \left( ||\boldsymbol{z}_p(0) - \boldsymbol{z}(0)||_H e^{-\xi t} + \int_0^t L_1 e^{-(\zeta/\epsilon)\tau} e^{-\xi(t-\tau)} d\tau + \frac{L_2 \epsilon}{\xi} \right) \\ &\leq \sqrt{\frac{\lambda^*}{\lambda_*}} \left( ||\boldsymbol{z}_p(0) - \boldsymbol{z}(0)||_H e^{-\xi t} + \frac{L_1}{\zeta} \frac{\epsilon}{1 - \epsilon\xi/\zeta} + \frac{L_2 \epsilon}{\xi} \right). \end{aligned}$$

The desired result holds for $\epsilon^* = \zeta/(2\xi)$ and $L^* = 2L_1/\zeta + L_2/\xi$. ■

## 1.2 Solutions of diffusing states converge to the null space of the differential operator $\boldsymbol{\mathcal{L}}_{\boldsymbol{v}}$

Let $\boldsymbol{v} : [0,1] \to \mathbb{R}_+^{n_d}$ be a smooth vector-valued function and $\boldsymbol{V}(x) = \text{diag}(\boldsymbol{v}(x))$. For the state vectors $\boldsymbol{z}_s(t,x) \in H_s := L^2((0,1), \mathbb{R}^{n_s}), \forall t \geq 0$ and $\boldsymbol{z}_d(t,x) \in H_d := L^2_{\boldsymbol{v}}((0,1), \mathbb{R}^{n_d}), \forall t \geq 0$, consider the following reaction-diffusion system:

$$\begin{aligned} &\frac{\partial \boldsymbol{z}_s(t,x)}{\partial t} = \boldsymbol{f}_s(t,x,\boldsymbol{z}_s,\boldsymbol{z}_d), \quad t \geq 0, x \in [0,1], \\ &\frac{\partial \boldsymbol{z}_d(t,x)}{\partial t} = \frac{1}{\epsilon} \boldsymbol{D} \boldsymbol{\mathcal{L}}_{\boldsymbol{v}}(\boldsymbol{z}_d) + \boldsymbol{f}_d(t,x,\boldsymbol{z}_s,\boldsymbol{z}_d), \quad t \geq 0, x \in (0,1), \\ &\boldsymbol{V}^2(x) \frac{d}{dx} \left[ \boldsymbol{V}^{-1}(x) \boldsymbol{z}_d(t,x) \right] \Big|_{x=0,1} = \boldsymbol{0}_n \quad t > 0, \\ &\boldsymbol{z}_s(0,x) = \boldsymbol{z}_{s,0}(x), \quad x \in [0,1], \\ &\boldsymbol{z}_d(0,x) = \boldsymbol{z}_{d,0}(x), \quad x \in [0,1], \end{aligned} \tag{7a}$$

where $\epsilon \in \mathbb{R}_+$, $\boldsymbol{f}_s : [0,\infty) \times [0,1] \times \mathbb{R}^{n_s} \times \mathbb{R}^{n_d} \to \mathbb{R}^{n_s}$ and $\boldsymbol{f}_d : [0,\infty) \times [0,1] \times \mathbb{R}^{n_s} \times \mathbb{R}^{n_d} \to \mathbb{R}^{n_d}$ are smooth functions, $\boldsymbol{D} = \text{diag}([D_1, \ldots, D_{n_d}]^T)$ such that $D_i > 0$ for all $i = 1, \ldots, n_d$, $\boldsymbol{z}_{s,0}^i : [0,1] \to \mathbb{R}_+$, $\boldsymbol{z}_{d,0}^i : [0,1] \to \mathbb{R}_+$, and

$$\boldsymbol{\mathcal{L}}_{\boldsymbol{v}}(\boldsymbol{z}_d) = \frac{d}{dx} \left[ \boldsymbol{V}^2(x) \frac{d}{dx} \left[ \boldsymbol{V}^{-1}(x) \boldsymbol{z}_d \right] \right] \tag{7b}$$

is such that $\boldsymbol{\mathcal{L}}_{\boldsymbol{v}} = [\mathcal{L}_{\boldsymbol{v}^1}(\boldsymbol{z}_d^1), \ldots, \mathcal{L}_{\boldsymbol{v}^{n_d}}(\boldsymbol{z}_d^{n_d})]^T$ where $\mathcal{L}_{\boldsymbol{v}^i}(\boldsymbol{z}_d^i)$ is an in Definition 1. The elements of $\boldsymbol{z}_d$ may be thought of as "freely diffusing" and those of $\boldsymbol{z}_s$ as "spatially fixed". For the general system (7), we show in this section that the spatial profiles of the diffusing species $\boldsymbol{z}_d$, approach those of $\boldsymbol{v}(x)$, that is, the null space of $\boldsymbol{\mathcal{L}}_{\boldsymbol{v}}$ when $\epsilon \ll 1$.

Assumption 1. Consider the system given by (7)

I There exists a positively invariant set $\chi \subset \mathbb{R}^{n_s+n_d}$ such that if $[\boldsymbol{z}_s^T(0,x), \boldsymbol{z}_d^T(0,x)]^T \in \chi, \forall x \in [0,1]$, then $[\boldsymbol{z}_s^T(t,x), \boldsymbol{z}_d^T(t,x)]^T \in \chi, \forall x \in [0,1], \forall t \geq 0$.

II There exists a positive constant $M > 0$ such that

$$||\boldsymbol{f}_d(t,x,\boldsymbol{z}_s,\boldsymbol{z}_d)||_{H_d} \leq M, \quad \forall t \geq 0, \forall [\boldsymbol{z}_s^T, \boldsymbol{z}_d^T]^T \in \chi.$$

Let $\boldsymbol{z}_d^{\perp}(t,x) = \boldsymbol{z}_d(t,x) - \hat{\boldsymbol{V}}(x)\bar{\boldsymbol{z}}_d(t)$ where $\bar{\boldsymbol{z}}_d(t) = \int_0^1 \boldsymbol{z}_d(t,x)dx$ and $\hat{\boldsymbol{V}}(x) = \left[\int_0^1 \boldsymbol{V}(x)dx\right]^{-1} \boldsymbol{V}(x)$. The dynamics of $\boldsymbol{z}_d^{\perp}(t,x)$ are given by

$$\begin{aligned}
\frac{\partial \boldsymbol{z}_d^{\perp}(t,x)}{\partial t} &= \frac{1}{\epsilon}\boldsymbol{D}\boldsymbol{\mathcal{L}}_{\boldsymbol{v}}(\boldsymbol{z}_d^{\perp}) + \boldsymbol{f}_d^{\perp}(t,x,\boldsymbol{z}_s,\boldsymbol{z}_d), \quad t > 0, x \in (0,1), \\
\boldsymbol{V}(x)\frac{d}{dx}\left[\boldsymbol{V}^{-1}(x)\boldsymbol{z}_d^{\perp}(t,x)\right]\Big|_{x=0,1} &= \boldsymbol{0}_n \quad t > 0, \\
\boldsymbol{z}_d^{\perp}(0,x) &= \boldsymbol{z}_{d,0}(x) - \hat{\boldsymbol{V}}(x)\bar{\boldsymbol{z}}_d(0), \quad x \in (0,1),
\end{aligned} \tag{7c}$$

where

$$\boldsymbol{f}_d^{\perp}(t,x,\boldsymbol{z}_s,\boldsymbol{z}_d) = \boldsymbol{f}_d(t,x,\boldsymbol{z}_s,\boldsymbol{z}_d) - \hat{\boldsymbol{V}}(x)\int_0^1 \boldsymbol{f}_d(t,x,\boldsymbol{z}_s,\boldsymbol{z}_d)dx.$$

The following theorem is a direct consequence of the operator $\boldsymbol{\mathcal{L}}_{\boldsymbol{v}}$ from Definition 1 being self-adjoint and negative semi-definite. This result will show that the infinite dimensional left over dynamics $\boldsymbol{z}_d^{\perp}(t,x)$ become order $\epsilon$ after a fast transient.

**Theorem 2.** *Consider the system defined by (7). Suppose that Assumption 1 holds and $[\boldsymbol{z}_{s,0}^T(x), \boldsymbol{z}_{d,0}^T(x)]^T \in \chi, \forall x \in [0,1]$, where $\chi$ is defined in Assumption 1-I. Then, there exists $\zeta, L_{\perp} > 0$ such that for all $\epsilon > 0$, the solution $\boldsymbol{z}_d^{\perp}(t,x)$ of (7c), satisfies*

$$||\boldsymbol{z}_d^{\perp}(t,x)||_{H_d} \leq ||\boldsymbol{z}_d^{\perp}(0,x)||_{H_d} e^{-\zeta t/\epsilon} + L_{\perp}\epsilon, \quad \forall t \geq 0.$$

*Proof:*

Let $\psi(t) = ||\boldsymbol{z}_d^{\perp}(t,x)||_{H_d}^2/2$ and $\tau = t/\epsilon$ thus

$$\frac{d\psi(\tau)}{d\tau} = \langle \boldsymbol{z}_d^{\perp}, \frac{\partial \boldsymbol{z}_d^{\perp}}{\partial \tau}\rangle_{\boldsymbol{v}} = \langle \boldsymbol{z}_d^{\perp}, \boldsymbol{D}\boldsymbol{\mathcal{L}}_{\boldsymbol{v}}(\boldsymbol{z}_d^{\perp})\rangle_{\boldsymbol{v}} + \epsilon\langle \boldsymbol{z}_d^{\perp}, \boldsymbol{f}_d^{\perp}(t,x,\boldsymbol{z}_s,\bar{\boldsymbol{z}}_d,\boldsymbol{z}_d^{\perp})\rangle_{\boldsymbol{v}}.$$

The following proof uses a similar logic as the the min-max theorem for matrices [4] to derive and upper bound for $\langle \boldsymbol{z}_d^{\perp}, \boldsymbol{D}\boldsymbol{\mathcal{L}}_{\boldsymbol{v}}(\boldsymbol{z}_d^{\perp})\rangle_{\boldsymbol{v}}$. Let $\lambda_{i,j}$ and $\psi_{i,j}$ denote the $j$-th eigenvalue and eigenfunction, respectively, of $\mathcal{L}_{\boldsymbol{v}^i}(\cdot)$ (i.e,. $\mathcal{L}_{\boldsymbol{v}^i}(\psi_{i,j}) = \lambda_{i,j}\psi_{i,j}$). Recall that $\{\psi_{i,j}\}$ forms a complete orthonormal basis for $L^2_{\boldsymbol{v}^i}(0,1)$ (Lemma 1-II). From the orthonormality of the eigenfunctions, linearity of $\boldsymbol{\mathcal{L}}_{\boldsymbol{v}}^i = \mathcal{L}_{\boldsymbol{v}^i}$, and the ordering of eigenvalues (Lemma 1-I), notice that:

$$
\begin{aligned}
\langle \boldsymbol{z}_d^{\perp}, \boldsymbol{D}\boldsymbol{\mathcal{L}}_{\boldsymbol{v}}(\boldsymbol{z}_d^{\perp})\rangle_{\boldsymbol{v}} &= \sum_{i=1}^{n_d}\langle \boldsymbol{z}_d^{\perp,i}, D_i\boldsymbol{\mathcal{L}}_{\boldsymbol{v}}^i(\boldsymbol{z}_d^{\perp,i})\rangle_{\boldsymbol{v}^i} \\
&= \sum_{i=1}^{n_d}\Big\langle \sum_{j=2}^{\infty}\langle \boldsymbol{z}_d^{\perp,i}, \psi_{i,j}\rangle_{\boldsymbol{v}^i}\psi_{i,j}, D_i\boldsymbol{\mathcal{L}}_{\boldsymbol{v}}^i\big(\sum_{j=2}^{\infty}\langle \boldsymbol{z}_d^{\perp,i}, \psi_{i,j}\rangle_{\boldsymbol{v}^i}\psi_{i,j}\big)\Big\rangle_{\boldsymbol{v}^i} \\
&= \sum_{i=1}^{n_d}\Big\langle \sum_{j=2}^{\infty}\langle \boldsymbol{z}_d^{\perp,i}, \psi_{i,j}\rangle_{\boldsymbol{v}^i}\psi_{i,j}, \sum_{j=2}^{\infty}\langle \boldsymbol{z}_d^{\perp,i}, \psi_{i,j}\rangle_{\boldsymbol{v}^i}D_i\boldsymbol{\mathcal{L}}_{\boldsymbol{v}}^i\big(\psi_{i,j}\big)\Big\rangle_{\boldsymbol{v}^i} \\
&= \sum_{i=1}^{n_d}\Big\langle \sum_{j=2}^{\infty}\langle \boldsymbol{z}_d^{\perp,i}, \psi_{i,j}\rangle_{\boldsymbol{v}^i}\psi_{i,j}, \sum_{j=2}^{\infty}D_i\lambda_{i,j}\langle \boldsymbol{z}_d^{\perp,i}, \psi_{i,j}\rangle_{\boldsymbol{v}^i}\psi_{i,j}\Big\rangle_{\boldsymbol{v}^i} \\
&= \sum_{i=1}^{n_d}\sum_{j=2}^{\infty}D_i\lambda_{i,j}\langle \boldsymbol{z}_d^{\perp,i}, \psi_{i,j}\rangle_{\boldsymbol{v}^i}^2 \\
&\le \sum_{i=1}^{n_d}D_i\lambda_{i,2}\sum_{j=2}^{\infty}\langle \boldsymbol{z}_d^{\perp,i}, \psi_{i,j}\rangle_{\boldsymbol{v}^i}^2 \\
&= \sum_{i=1}^{n_d}D_i\lambda_{i,2}\langle \boldsymbol{z}_d^{\perp,i}, \boldsymbol{z}_d^{\perp,i}\rangle_{\boldsymbol{v}^i} \\
&\le -|\bar{\lambda}_2|\langle \boldsymbol{z}_d^{\perp}, \boldsymbol{z}_d^{\perp}\rangle_{\boldsymbol{v}} \\
&= -2|\bar{\lambda}_2|\psi(\tau)
\end{aligned}
$$

where $\bar{\lambda}_2 = \max_{i=1,\dots n_d}(\{D_i\lambda_{2,i}\})$. The fact that $[\boldsymbol{z}_{s,0}^T(x), \boldsymbol{z}_{d,0}^T(x)]^T \in \chi, \forall x \in [0,1]$ implies that $[\boldsymbol{z}_s^T(t,x), \boldsymbol{z}_d^T(t,x)]^T \in \chi, \forall x \in [0,1], \forall t \geq 0$ by Assumption 1-I. By the cauchy-Schwarz inequality, Assumption 1-II, and the fact that $\boldsymbol{f}_d^{\perp}$ is a projection of $\boldsymbol{f}_d$ (thus $||\boldsymbol{f}_d^{\perp}||_{H_d}|| \leq ||\boldsymbol{f}_d||_{H_d}$) we have that $|\langle \boldsymbol{z}_d^{\perp}, \boldsymbol{f}_d^{\perp}\rangle_{\boldsymbol{v}}| \leq ||\boldsymbol{f}_d^{\perp}||_{H_d}||\boldsymbol{z}_d^{\perp}||_{H_d} \leq M||\boldsymbol{z}_d^{\perp}||_{H_d}$. Let $\nu \in (0,1)$ thus

$$
\begin{aligned}
\frac{d\psi(\tau)}{d\tau} \leq& -2|\bar{\lambda}_2|\psi(\tau) + \epsilon M\psi^{1/2}(\tau) \\
\leq& -2(1-\nu)|\bar{\lambda}_2|\psi(\tau) - 2\nu|\bar{\lambda}_2|\psi(\tau) + \epsilon M\psi^{1/2}(\tau) \\
\leq& -2(1-\nu)|\bar{\lambda}_2|\psi(\tau), \quad \forall\psi > \big[\frac{\epsilon M}{2\nu|\bar{\lambda}_2|}\big]^2.
\end{aligned}
$$

Therefore,

$$
\psi(\tau) \leq \psi(0)e^{-2(1-\nu)|\bar{\lambda}_2|\tau} + \big[\frac{\epsilon M}{2\nu|\bar{\lambda}_2|}\big]^2, \forall\tau \geq 0.
$$

Finally,

$$
||\boldsymbol{z}_d^{\perp}(t,x)||_{H_d} \leq ||\boldsymbol{z}_d^{\perp}(0,x)||_{H_d}e^{-\zeta t/\epsilon} + \frac{M}{\sqrt{2}\nu|\bar{\lambda}_2|}\epsilon,
$$

where $\zeta = (1-\nu)|\bar{\lambda}_2|$ and $L_{\perp} = \frac{M}{\sqrt{2}\nu|\bar{\lambda}_2|}$. ■

### 1.3 Model Reduction of Enzymatic-like Reaction In the Limit of Fast diffusion

The spatial-temporal dynamics of $\mathrm{E_i}$, S, and $\mathrm{c_i}$ as described by the biochemical reactions in the Main Text, Equation 1, are given in dimensionless form by

$$
\begin{aligned}
\frac{\partial E_i(t,x)}{\partial t} &= -\frac{d}{dx}\big[J(x,E_i)\big] + \alpha_i(t,x) + \frac{1}{\eta_i}\Big[-E_i(t,x)S(t,x)\frac{\tilde{a}_i}{\tilde{d}_i} + c_i(t,x)\Big] \\
&\qquad - (\gamma_i+1)(E_i(t,x)+c_i(t,x)), \\
\frac{\partial c_i(t,x)}{\partial t} &= -\frac{d}{dx}\big[J(x,c_i)\big] + \frac{1}{\eta_i}\Big[E_i(t,x)S(t,x)\frac{\tilde{a}_i}{\tilde{d}_i} - c_i(t,x)\Big], \qquad (8a)\\
\frac{\partial S(t,x)}{\partial t} &= -\frac{d}{dx}\big[J(x,S)\big] + \alpha_s(t,x) + \sum_{j=1}^{n}\frac{1}{\eta_j}\Big[-E_j(t,x)S(t,x)\frac{\tilde{a}_j}{\tilde{d}_j} + c_j(t,x)\Big] \\
&\qquad - (\gamma_s+1)(S(t,x)+\sum_{j=1}^{n}c_j(t,x)),
\end{aligned}
$$

where the flux term and the boundary conditions are given in Table 1 for three cases of interest, $\eta_i = 1/\tilde{d}_i$, and $\tilde{d}_i = \gamma_i + \gamma_s + d_i + \kappa_i + 1$. If applicable, $v_{E_i}(x)$, $v_s(x)$, $v_{c_i}(x)$ are the available volume profiles of $\mathrm{E_i}$, S, and $\mathrm{c_i}$, respectively, and let

$$
\hat{v}_{E_i}(x) = \frac{v_{E_i}(x)}{\int_0^1 v_{E_i}(x)dx}, \quad \hat{v}_s(x) = \frac{v_s(x)}{\int_0^1 v_s(x)dx}, \quad \hat{v}_{c_i}(x) = \frac{v_{c_i}(x)}{\int_0^1 v_{c_i}(x)dx}. \qquad (8b)
$$

| Case | I: All species diffuse | II: Substrate diffuses and enzymes fixed | III: Enzymes diffuses and substrate fixed |
|---|---|---|---|
| **Dimensionless Flux** | $-\frac{d}{dx}[J(x,E_i)] = \frac{1}{\epsilon}\chi_{E_i}\mathcal{L}_{v_{E_i}}(E_i)$ <br> $-\frac{d}{dx}[J(x,S)] = \frac{1}{\epsilon}\mathcal{L}_{v_S}(S)$ <br> $-\frac{d}{dx}[J(x,c_i)] = \frac{1}{\epsilon}\chi_{c_i}\mathcal{L}_{v_{c_i}}(c_i)$ | $-\frac{d}{dx}[J(x,E_i)] = 0$ <br> $-\frac{d}{dx}[J(x,S)] = \frac{1}{\epsilon}\mathcal{L}_{v_S}(S)$ <br> $-\frac{d}{dx}[J(x,c_i)] = 0$ | $-\frac{d}{dx}[J(x,E_i)] = \frac{1}{\epsilon}\mathcal{L}_{v_{E_i}}(E_i)$ <br> $-\frac{d}{dx}[J(x,S)] = 0$ <br> $-\frac{d}{dx}[J(x,c_i)] = 0$ |
| **Boundary conditions** | $J(0,E_i) = J(1,E_i) = 0$ <br> $J(0,c_i) = J(1,c_i) = 0$ <br> $J(0,S) = J(1,S) = 0$ | $J(0,S) = J(1,S) = 0$ | $J(0,E_i) = J(1,E_i) = 0$ |
| $\epsilon$ | $(\mu L^2)/D_s$ | $(\mu L^2)/D_s$ | $(\mu L^2)/D_{E_1}$ |
| **Dimensionless diffusion** | $\chi_{E_i} = D_{E_i}/D_s, \chi_{c_i} = D_{c_i}/D_s$ | | |
| **Location of fixed species** | N/A | $x_i^*$ | $x_s^*$ |

Table 1: The flux dynamics and the boundary conditions corresponding to (8) for Cases I-III. Here $v_{E,i}(x)$, $v_S(x)$, and $v_{c,i}(x)$, are the available volume profiles of $\mathrm{E_i}$, S, and $\mathrm{c_i}$, respectively, and $\mathcal{L}_v$ is as in Definitions 1. The parameters $D_{E_i}$, $D_{c_i}$, and $D_s$, are the enzyme, complex, and substrate diffusion coefficients, respectively, $\epsilon$ is a dimensionless parameter that captures the speed of diffusion (with respect to dilution). A species being spatially fixed translates to the flux being zero throughout the whole spatial domain. In Case II, we denote the location of the fixed species $\mathrm{E_i}$, as $x_i^* \in (0,1)$ for $i = 1,\ldots,n$. In Case III, we denote the location of the fixed species S, as $x_s^* \in (0,1)$.

We denote $\bar{E}_i(t)$, $\bar{S}(t)$, and $\bar{c}_i(t)$ to be the space averaged enzyme, substrate, and complex concentrations, respectively (e.g., $\bar{E}_i(t) = \int_0^1 E_i(t,x)dx$). The dynamics governing these space averaged variables are derived by integrating (8) in space and applying the boundary conditions and are given by:

$$
\frac{d\bar{E}_i(t)}{dt} = \bar{\alpha}_i(t) - \frac{1}{\eta_i}\big[\frac{\tilde{a}_i}{\tilde{d}_i}[\int_0^1 E_i(t,x)S(t,x)dx] - \bar{c}_i(t)\big] - (\gamma_i+1)(\bar{E}_i(t)+\bar{c}_i(t)), \qquad (9a)
$$

$$
\frac{d\bar{c}_i(t)}{dt} = \frac{1}{\eta_i}\big[\frac{\tilde{a}_i}{\tilde{d}_i}[\int_0^1 E_i(t,x)S(t,x)dx] - \bar{c}_i(t)\big], \qquad (9b)
$$

$$
\frac{d\bar{S}(t)}{dt} = \bar{\alpha}_s(t) - \sum_{j=1}^{n}\frac{1}{\eta_j}\Big[\frac{\tilde{a}_j}{\tilde{d}_j}[\int_0^1 E_j(t,x)S(t,x)dx] - \bar{c}_j(t)\Big] - (\gamma_s+1)(\bar{S}(t)+\sum_{j=1}^{n}\bar{c}_j(t)), \qquad (9c)
$$

where overbars denote spatially averaged variables. We make the follow assumptions necessary to state our main result

Assumption 2. Consider (8), for $i = 1, \dots, n$, assume that

I the functions $\alpha_i(t,x)$ and $\alpha_s(t,x)$ are smooth in each argument

II there exists constant $\tilde{\alpha}_i > 0$ such that $0 < \alpha_i(t,x) \leq \tilde{\alpha}_i, \forall t \geq 0, \forall x \in [0,1]$

III there exists constant $\tilde{\alpha}_s > 0$ such that $0 \leq \alpha_s(t,x) \leq \tilde{\alpha}_s, \forall t \geq 0, \forall x \in [0,1]$

IV the functions $v_{E,i}(x)$, $v_S(x)$, and $v_{c_i}(x) = v_{E,i}(x)v_S(x)$ (as in Table 1) are smooth, strictly greater than zero, and bounded above by unity.

The following assumption makes precise what it means for the spatially fixed species $\mathrm{E_i}$ (in Case II) and S (in Case III) to be spatially localized at $x_i^*$ and $x_s^*$, respectively.

Assumption 3. (Localization of spatially fixed species) Consider the system (8). Let $x_i^* \in (0,1)$ for Case II and $x_s^* \in (0,1)$ for Case III, be given by Table 1. Let $\delta^* = \min(x_1, \dots, x_n, 1-x_1, \dots, 1-x_n)$ for Case II and $\delta^* = \min(x_s^*, 1-x_s^*)$ for Case III. We assume that for a given $\delta > 0$ such that $\delta < \delta^*$, the functions $\alpha_i(t,x)$ and $\alpha_s(t,x)$ satisfy

- for Case II: $\alpha_i(t,x) \leq \delta$, for all $x \notin [x_i^* - \delta, x_i^* + \delta], \quad \forall t \geq 0$
- for Case III: $\alpha_s(t,x) \leq \delta$, for all $x \notin [x_s^* - \delta, x_s^* + \delta], \quad \forall t \geq 0$,

Furthermore, we assume for Cases I-III, that there exists $\bar{\alpha}_s^* > 0$, $\bar{\alpha}_{i,*} > 0$, and $\bar{\alpha}_i^* > 0$ for $i = 1, \dots, n$ independent of $\delta$, such that $\bar{\alpha}_{i,*} < \int_0^1 \alpha_i(t,x)dx \leq \bar{\alpha}_i^*, \forall t \geq 0$ and $\int_0^1 \alpha_s(t,x)dx \leq \bar{\alpha}_s^*, \forall t \geq 0$.

The following definition will provide the candidate reduced model to approximates (9).

**Definition 3.** (Reduced space-averaged dynamics) Let $x_i^* \in (0,1)$ for Case II and $x_s^* \in (0,1)$ for Case III, be given by Table 1. For $i = 1, \dots, n$, consider the system

$$\begin{aligned}
\frac{d\hat{\bar{E}}_i(t)}{dt} &= \bar{\alpha}_i(t) - \frac{1}{\eta_i}\big[\hat{\bar{E}}_i(t)\hat{\bar{S}}(t)\frac{\tilde{a}_i\theta_i^*}{\tilde{d}_i} - \hat{\bar{c}}_i(t)\big] - (\gamma_i+1)(\hat{\bar{E}}_i(t) + \hat{\bar{c}}_i(t), \\
\frac{d\hat{\bar{c}}_i(t)}{dt} &= \frac{1}{\eta_i}\big[\hat{\bar{E}}_i(t)\hat{\bar{S}}(t)\frac{\tilde{a}_i\theta_i^*}{\tilde{d}_i} - \hat{\bar{c}}_i(t)\big], \\
\frac{d\bar{S}(t)}{dt} &= \bar{\alpha}_s(t) - \sum_{j=1}^{n}\frac{1}{\eta_j}\left[\hat{\bar{E}}_j(t)\hat{\bar{S}}(t)\frac{\tilde{a}_j\theta_j^*}{\tilde{d}_j} - \hat{\bar{c}}_i(t)\right] - (\gamma_s+1)(\hat{\bar{S}}(t) + \sum_{j=1}^{n}\hat{\bar{c}}_j(t)),
\end{aligned} \tag{10a}$$

$$\theta_i^* = \begin{cases} \int_0^1 \hat{v}_{E_i}(x)\hat{v}_S(x)dx & \text{for Case I} \\ \hat{v}_S(x_i^*) & \text{for Case II} \\ \hat{v}_{E_i}(x_s^*) & \text{for Case III} \end{cases}, \tag{10b}$$

where $\hat{\bar{E}}_i(0) = \bar{E}_i(0)$, $\hat{\bar{S}}(0) = \bar{S}(0)$, $\hat{\bar{c}}_i(0) = \bar{c}_i(0)$, as given by (9),and $\hat{v}_{E_i}(x)$, $\hat{v}_{c_i}(x)$, and $\hat{v}_S(x)$ are given by (8b).

**Theorem 3.** *Consider the system (8) and let*

$$\boldsymbol{z}(t,x) = [E_1(t,x), \dots, E_n(t,x), c_1(t,x), \dots, c_n(t,x), S(t,x)]^T, \quad \bar{\boldsymbol{z}}(t) = \int_0^1 \boldsymbol{z}(t,x)dx \tag{11}$$

*Let $\epsilon > 0$, $x_i^* \in (0,1)$ and $x_s^* \in (0,1)$ be given for Case I-III by Table 1. Let $\hat{\bar{E}}_i(t)$, $\hat{\bar{S}}(t)$, and $\hat{\bar{c}}_i(t)$ be as in Definition 3 and let*

$$\hat{\bar{\boldsymbol{z}}}(t) = [\hat{\bar{E}}_1(t), \dots, \hat{\bar{E}}_n(t), \hat{\bar{c}}_1(t), \dots, \hat{\bar{c}}_n(t), \hat{\bar{S}}(t)]^T.$$

*Suppose that Assumptions 2 holds for Cases I-III. Then, there exists $L_1, \epsilon^* > 0$, $\Omega_z \subset L^2((0,1), \mathbb{R}^{2n+1})$ and $\Omega_{\bar{z}} \subset \mathbb{R}^{2n+1}$ such that for all $\boldsymbol{z}(0,x) \in \Omega_z$, $\bar{\boldsymbol{z}}(0) \in \Omega_{\bar{z}}$, $0 < \epsilon < \epsilon^*$, the solutions $\bar{\boldsymbol{z}}(t)$ and $\hat{\bar{\boldsymbol{z}}}(t)$ satisfy*

$$||\bar{\boldsymbol{z}}(t) - \hat{\bar{\boldsymbol{z}}}(t)||_{\mathbb{R}^{2n+1}} \leq |\Delta_z|, \quad \forall t \geq 0, \tag{12}$$

*where $|\Delta_z| = L_1\epsilon$ for Case I. For Cases II-III, if in addition, Assumption 3 holds for all $0 < \delta < \delta^*$, there exists $L_3 > 0$ such that for all $0 < \delta < \delta^*$, there exists $L_2(\delta)$ and $\Omega_{z,\delta} \subset L^2((0,1), \mathbb{R}^{2n+1})$ such that (12) holds for all $\boldsymbol{z}(0,x) \in \Omega_{z,\delta}$, $\bar{\boldsymbol{z}}(0) \in \Omega_{\bar{z}}$, $0 < \epsilon < \epsilon^*$ with $|\Delta_z| = L_2(\delta)\epsilon + L_3\delta$.*

*Remark* 2. The constant $L_2(\delta)$, guaranteed to exist in Theorem 3 for Cases II-III, depends on $\delta$. Therefore, for a given $\eta > 0$, if one wishes to to have $||\bar{\boldsymbol{z}}(t) - \hat{\bar{\boldsymbol{z}}}(t)||_{\mathbb{R}^{2n+1}} \leq \eta, \quad \forall t \geq 0$, then one would chose $\delta$ such that $L_3\delta < \eta$ and then choose $\epsilon$ sufficiently small (depending on $\delta$) such that $L_2(\delta)\epsilon + L_3\delta \leq \eta$.

*Remark* 3. The set $\Omega_z$, guaranteed to exist in Theorem 3, depends on $\delta$ in Cases II-III and this dependence is made precise in the proof.

**Road map of proof**: The rest of this section is dedicated towards proving Theorem 3. We first apply Theorem 2 to show that the spatial profile of a freely diffusing species converges to its available volume profile (e.g,. $v_{E,i}(x)$, $v_S(x)$, and $v_{c_i}(x)$) exponentially fast in the time scale associated with diffusion. If the localization assumption holds, then (9) has the form described by Definition 3, but with additional "disturbance" terms of order $\epsilon$ and $\delta$. We proceed to demonstrate the system described in Definition 3 is contracting and apply the robustness property of contracting systems (Lemma 3) to show closeness between its solutions and those of (9).

The following result will define a positively invariant and bounded subset of $\mathbb{R}^{2n+1}$ such that solutions to (8) starting within this set at $t = 0$, remain within this set for all times and spatial values. To apply Theorem 2 to (8), the existence of such a positively invariant set is required by Assumption 1.

**Claim** 1. Consider the system given by (8), with Cases I-III specified by Table 1. Suppose Assumptions 2 holds. Let $\chi = \{\boldsymbol{z} \in \mathbb{R}^{2n+1} : \boldsymbol{z}^i \geq 0, \forall i \text{ and } \boldsymbol{z}^T\vec{n} \leq \Gamma\}$, where

$$\vec{n} = [\boldsymbol{b}^T, 2\boldsymbol{b}^T, 1], \quad \Gamma = \big(\sum_{j=1}^{n} \tilde{\alpha}_j + \tilde{\alpha}_S\big)/v_*, \quad v_* = \begin{cases} \min_{i=1}^{n} \min_{x\in[0,1]} \boldsymbol{v}_c & \text{for Case I} \\ \min_{x\in[0,1]} v_S(x) & \text{for Case II} \\ \min_{i=1}^{n} \min_{x\in[0,1]} \boldsymbol{v}_E & \text{for Case III} \end{cases},$$

$$\boldsymbol{b} = [1, \ldots, 1]^T \in \mathbb{R}^n, \quad \boldsymbol{v}_E = [v_{E_1}(x), \ldots, v_{E_n}(x)]^T, \quad \boldsymbol{v}_c = [v_{c_1}(x), \ldots, v_{c_n}(x)]^T.$$

Let $\boldsymbol{z}(t, x)$ be given by (11), if $\boldsymbol{z}(0, x) \in \chi, \forall x \in [0, 1]$, then $\boldsymbol{z}(t, x) \in \chi, \forall t \geq 0, \forall x \in [0, 1]$. Thus $\chi$ defines a positively invariant set of (8).

*Proof:* We apply Theorem 1 in [5], which states that for a parabolic PDE system with sufficiently smooth coefficients, a closed convex subset of euclidean space is positively invariant if the vector field corresponding to the "reaction dynamics" never points outwards at the boundaries of the set. To apply this theorem we first make a coordinate transformation. The spatial differential operator (as in Definition 1) for a general diffusing species $y(t, x)$ with available volume profile $v(x)$, given by

$$\mathcal{L}_v(y) = \frac{d}{dx}\big[v^2(x)\frac{d}{dx}(\frac{y(t,x)}{v(x)})\big] = v(x)\frac{\partial^2 y(t,x)}{\partial x^2} - y(t,x)\frac{\partial^2 v(x)}{\partial x^2},$$

is not in the standard form stipulated by Equation 1.2 in [5]. Therefore, for Cases I-III, the following coordinate transformation is made

$$\boldsymbol{u}(t, x) = \Lambda^{-1}\boldsymbol{z}(t, x), \quad \Lambda = \begin{cases} \text{diag}([\boldsymbol{v}_E^T, \boldsymbol{v}_c^T, v_S]^T) & \text{for Case I} \\ \text{diag}([\boldsymbol{b}^T, \boldsymbol{b}^T, v_S]^T) & \text{for Case II} \\ \text{diag}([\boldsymbol{v}_E^T, \boldsymbol{b}^T, 1]^T) & \text{for Case III} \end{cases}. \tag{13}$$

With the transformation $u(t, x) = y(t, x)/v(x)$, the differential operator $\hat{\mathcal{L}}_v$ given by

$$\hat{\mathcal{L}}_v(u) := \frac{1}{v}\mathcal{L}_v(uv) = v(x)\frac{\partial^2 u(t,x)}{\partial x^2} + 2\frac{\partial v(x)}{\partial x}\frac{\partial u(t,x)}{\partial x},$$

is in the postulated form. Let

$$\begin{aligned} f_{E_i}(t, x, \boldsymbol{z}) =& \alpha_i(t, x) - \frac{1}{\eta_i}\big[E_i(t, x)S(t, x)\frac{\tilde{a}_i}{\tilde{d}_i} - c_i(t, x)\big] - (\gamma_i + 1)(E_i(t, x) + c_i(t, x)), \\ f_{c_i}(t, x, \boldsymbol{z}) =& \frac{1}{\eta_i}\big[E_i(t, x)S(t, x)\frac{\tilde{a}_i}{\tilde{d}_i} - c_i(t, x)\big], \\ f_s(t, x, \boldsymbol{z}) =& \alpha_s(t, x) - \sum_{j=1}^{n}\frac{1}{\eta_j}\big[E_j(t, x)S(t, x)\frac{\tilde{a}_j}{\tilde{d}_j} - c_j(t, x)\big] - (\gamma_s + 1)(S(t, x) + \sum_{j=1}^{n} c_j(t, x)), \end{aligned} \tag{14}$$

and $\boldsymbol{f}(t,x,\boldsymbol{z}) = [f_{E_1},\dots,f_{E_n},f_{c_1},\dots,f_{c_n},f_s]^T$ is the vector field of the reaction dynamics of (8). The vector field corresponding to the reaction dynamics of the transformed system is given by $\hat{\boldsymbol{f}}(t,x,\boldsymbol{u}) := \Lambda^{-1}\boldsymbol{f}(t,x,\Lambda\boldsymbol{u})$. Let

$$\vec{n}_u(x) = \begin{cases} [\boldsymbol{v}_E^T, 2\boldsymbol{v}_c^T, v_S(x)]^T & \text{for Case I} \\ [\boldsymbol{b}^T, 2\boldsymbol{b}^T, v_S(x)]^T & \text{for Case II} \\ [\boldsymbol{v}_E^T, 2\boldsymbol{b}^T, 1]^T & \text{for Case III} \end{cases},$$

and $\chi_u(x) = \{\boldsymbol{u} \in \mathbb{R}^{2n+1} : \boldsymbol{u}^i \geq 0, \forall i \text{ and } \boldsymbol{u}^T\vec{n}_u(x) \leq \Gamma\}$. We now check that the vector field $\hat{\boldsymbol{f}}(t,x,\boldsymbol{u})$ does not point outward at all the boundary points of $\chi_u(x), \forall x \in [0,1]$. Checking that $\hat{\boldsymbol{f}}^i(t,x,\boldsymbol{u})\big|_{\boldsymbol{u}^i=0} \geq 0, \forall \boldsymbol{u} \in \chi_u(x), \forall x \in [0,1], \forall t \geq 0$ is equivalent to checking $\boldsymbol{f}^i(t,x,\boldsymbol{z})\big|_{\boldsymbol{z}^i=0} \geq 0$, thus notice that for $i = 1,\dots,n$

$$f_{E_i}(t,x,\boldsymbol{z})\big|_{E_i=0} = \alpha_i(t,x) + (d_i + \kappa_i + \gamma_s)c_i(t,x) \geq 0, \quad \forall \boldsymbol{z} \geq \boldsymbol{0}_{2n+1}, \forall x \in [0,1], \forall t \geq 0$$

$$f_{c_i}(t,x,\boldsymbol{z})\big|_{c_i=0} = \frac{1}{\eta_i}\big[E_i(t,x)S(t,x)\frac{\tilde{a}_i}{\tilde{d}_i}\big] \geq 0, \quad \forall \boldsymbol{z} \geq \boldsymbol{0}_{2n+1}, \forall x \in [0,1], \forall t \geq 0$$

and

$$f_s(t,x,\boldsymbol{z})\big|_{S=0} = \alpha_s(t,x) + \sum_{j=1}^{n}(d_j + \kappa_j + \gamma_j)c_j(t,x) \geq 0, \quad \forall \boldsymbol{z} \geq \boldsymbol{0}_{2n+1}, \forall x \in [0,1], \forall t \geq 0.$$

The set $\partial\chi_u(x) = \{\boldsymbol{u} \in \mathbb{R}^{2n+1} : \boldsymbol{u}^T(x)\vec{n}_u(x) = \Gamma\}$, corresponds to the boundary points defined by planar surface $\boldsymbol{u}^T(x)\vec{n}_u(x) = \Gamma$ with normal vector $\vec{n}_u(x)$, we need to check that for all boundary point $\boldsymbol{u}^* \in \partial\chi_u(x)$ we have that $\hat{\boldsymbol{f}}^T(t,x,\boldsymbol{u}^*) \leq 0, \forall x \in [0,1], \forall t \geq 0$:

$$\begin{aligned}
\hat{\boldsymbol{f}}^T(t,x,\boldsymbol{u}^*)\vec{n}_u(x) &= \big[\sum_{j=1}^{n}\alpha_j(t,x) - (\gamma_j+1)(\boldsymbol{u}^{j,*}\boldsymbol{\Lambda}^{j,j} + \boldsymbol{u}^{j+n,*}\boldsymbol{\Lambda}^{j+n,j+n}\big] \\
&\quad + \alpha_s(t,x) - (\gamma_s+1)(\boldsymbol{u}^{2n+1,*}\boldsymbol{\Lambda}^{2n+1,2n+1} + \sum_{j=1}^{n}\boldsymbol{u}^{j+n,*}\boldsymbol{\Lambda}^{j+n,j+n}) \\
&\leq \sum_{j=1}^{n}\tilde{\alpha}_j + \tilde{\alpha}_s - (\sum_{j=1}^{n}(\boldsymbol{u}^{j,*}\boldsymbol{\Lambda}^{j,j} + 2\boldsymbol{u}^{j+n,*}\boldsymbol{\Lambda}^{j+n,j+n}) + \boldsymbol{u}^{2n+1,*}\boldsymbol{\Lambda}^{2n+1,2n+1}) \\
&\leq \sum_{j=1}^{n}\tilde{\alpha}_j + \tilde{\alpha}_s - (\sum_{j=1}^{n}(\boldsymbol{u}^{j,*} + 2\boldsymbol{u}^{j+n,*}) + \boldsymbol{u}^{2n+1,*})v_*, \qquad \text{Assumption 2} \\
&\leq \big[\sum_{j=1}^{n}\tilde{\alpha}_j(x)\big] + \tilde{\alpha}_s(x) - \Gamma v_*, \quad \boldsymbol{u}^* \in \partial\chi_u(x), \forall x \in [0,1] \implies \boldsymbol{u}^{*,T}\vec{n} \leq \Gamma \\
&= \big[\sum_{j=1}^{n}\tilde{\alpha}_j\big] + \tilde{\alpha}_s - \big[\sum_{j=1}^{n}\tilde{\alpha}_j\big] - \tilde{\alpha}_s \\
&= 0, \quad \forall x \in [0,1], \forall t \geq 0.
\end{aligned}$$

Thus, $\chi_u(x)$ is a positively invariant set in the $\boldsymbol{u}(t,x)$ coordinates. The corresponding invariant set in the $\boldsymbol{z}(t,x)$ coordinates is given by $\chi$. ■

**Corollary** 1. *The positivity of $E_i(t,x)$, $S(t,x)$, and $c_i(t,x)$ imply the positivity of $\bar{E}_i(t) = \int_0^1 E_i(t,x)dx$, $\bar{S}(t) = \int_0^1 S(t,x)dx$, and $\bar{c}_i(t) = \int_0^1 c_i(t,x)dx$.*

**Definition** 4. Consider the systems given by (8) and (9) and let Cases I-III correspond to those in Table 1. We define

$$\begin{aligned}
&\text{For Case I and Case III:} && E_i^{\perp}(t,x) = E_i(t,x) - \bar{E}_i(t)\hat{v}_{E_i}(x), \\
&\text{For Case I:} && c_i^{\perp}(t,x) = c_i(t,x) - \bar{c}_i(t)\hat{v}_{c_i}(x), \qquad (15a) \\
&\text{For Case I and Case II:} && S^{\perp}(t,x) = S(t,x) - \bar{S}(t)\hat{v}_S(x),
\end{aligned}$$

and

$$\begin{aligned}
&\text{For Case I:} \quad \boldsymbol{w}^{\perp}(t,x) = [E_1^{\perp}, \dots, E_n^{\perp}, c_1^{\perp}, \dots, c_n^{\perp}, S^{\perp}]^T, \\
&\text{For Case II:} \quad \boldsymbol{w}^{\perp}(t,x) = S^{\perp}, \\
&\text{For Case III:} \quad \boldsymbol{w}^{\perp}(t,x) = [E_1^{\perp}, \dots, E_n^{\perp}]^T.
\end{aligned} \tag{15b}$$

**Lemma** 4. *Consider the systems given by (8) and let $\epsilon > 0$ be defined for Cases I-III by Table 1. Let $\boldsymbol{w}^{\perp}(t,x)$ be as in Definition 4 and suppose Assumption 2 holds. Let $\chi \subset \mathbb{R}^{2n+1}$ be as described in Claim 1 and let $\boldsymbol{z}(t,x)$ be given by (11). Then there exists $\zeta, L_{\perp} > 0$ such that for all $\boldsymbol{z}(0,x) \in \chi, \forall x \in [0,1]$ and $\epsilon > 0$, $\boldsymbol{w}^{\perp}(t,x)$ satisfies*

$$||\boldsymbol{w}^{\perp}(t,x)||_{L^2((0,1),\mathbb{R}^m)} \leq ||\boldsymbol{w}^{\perp}(0,x)||_{L^2((0,1),\mathbb{R}^m)} e^{\zeta t/\epsilon} + L_{\perp}\epsilon, \quad \forall t \geq 0,$$

*where $m = 2n+1$ in Case I, $m = 1$ in Case II, and $m = n$ in Case III.*

*Proof:* This results follows directly from Theorem 2 where Assumption 1-I is satisfied by $\chi$ and Assumption 1-II is satisfied by the smoothness of the reaction dynamics in (8), the compactness of the sets $\chi$ and $[0,1]$, and the temporal boundedness (Assumption 2). ■

*Remark* 4. From the proof of Theorem 2, one can observe that $L_{\perp}$ depends on the size of $\chi$. Once Assumption 3 is made, the size of $\chi$ will depend on $\delta$ for Case II-III. Thus, $L_{\perp}$ depends on $\delta$ for Cases II-III.

Next we define the space averaged total enzyme and substrate quantities and show that these are the same for (9) and (10). Furthermore, we will show the dynamics for these quantities are governed by uncoupled, linear, and contracting ODEs.

**Definition** 5. (Total space average enzyme and substrate concentrations) For (9), we define the total space averaged enzyme and substrate for $i = 1 \dots, n$ as $\bar{E}_{i,T}(t) = \bar{E}_i(t) + \bar{c}_i(t)$, $\bar{S}_T(t) = \bar{S}(t) + \sum_{j=1}^{n} \bar{c}_j(t)$, respectively, and similarly for (10), we define $\hat{\bar{E}}_{i,T}(t) = \hat{\bar{E}}_i(t) + \hat{\bar{c}}_i(t)$ and $\hat{\bar{S}}_T(t) = \hat{\bar{S}}(t) + \sum_{j=1}^{n} \hat{\bar{c}}_j(t)$, the dynamics of these quantities are given by

$$\frac{d\bar{E}_{i,T}(t)}{dt} = \bar{\alpha}_i(t) - (\gamma_i + 1)\bar{E}_{i,T}(t), \quad \frac{d\bar{S}_T(t)}{dt} = \bar{\alpha}_s(t) - (\gamma_s + 1)\bar{S}_T(t), \tag{16a}$$

$$\frac{d\hat{\bar{E}}_{i,T}(t)}{dt} = \bar{\alpha}_i(t) - (\gamma_i + 1)\hat{\bar{E}}_{i,T}(t), \quad \frac{d\hat{\bar{S}}_T(t)}{dt} = \bar{\alpha}_s(t) - (\gamma_s + 1)\hat{\bar{S}}_T(t). \tag{16b}$$

*Remark* 5. For $i = 1, \dots, n$, $\bar{E}_{i,T}(t) = \hat{\bar{E}}_{i,T}(t), \forall t \geq 0$ and $\bar{S}_T(t) = \hat{\bar{S}}_T(t), \forall t \geq 0$ since from Definition 3, we have that $\bar{E}_{i,T}(0) = \hat{\bar{E}}_{i,T}(0)$ and $\bar{S}_T(0) = \hat{\bar{S}}_T(0)$.

*Remark* 6. From the linear and uncoupled structure of (16), it is clear that the dynamics for $\bar{E}_{i,T}(t)$ are contracting with contraction rate $\lambda_i = \gamma_i + 1$ for all $i = 1, \dots, n$. Similarly, the $\bar{S}_T(t)$ dynamics are contracting with contraction rate $\lambda_s = \gamma_s + 1$.

**Claim** 2. Consider the systems (9), (10), and (16). Let $\bar{\alpha}_i^*$, $\bar{\alpha}_{i,*}$, and $\bar{\alpha}_s^*$ be as in Assumption 3. Assume that $\bar{E}_{i,T}(0) \leq \bar{E}_{i,T}^*$, and that $\bar{S}_T(0) \leq \bar{S}_T^*$. Then

$$(I) \quad \bar{S}(t) \leq \bar{S}_T^*, \forall t \geq 0, \quad (II) \quad \hat{\bar{S}}(t) \leq \bar{S}_T^*, \forall t \geq 0, \quad (III) \quad \bar{E}_i(t) \leq \bar{E}_{i,T}^*, \forall t \geq 0,$$

where $\bar{E}_{i,T}^* = \frac{\bar{\alpha}_i^*}{\gamma_i + 1}$ and $\bar{S}_T^* = \frac{\bar{\alpha}_s^*}{\gamma_s + 1}$. Furthermore, if we assume that $\bar{E}_{i,*} \leq \bar{E}_i(0)$, where $\bar{E}_{i,*} = \frac{\bar{\alpha}_{i,*}}{\gamma_i + 1 + \tilde{a}_i \bar{S}_T^*}$, then we have that

$$(IV) \quad \bar{E}_{i,*} \leq \hat{\bar{E}}_i(t) \leq \bar{E}_{i,T}^*, \forall t \geq 0.$$

*Proof:* Let $\hat{\bar{S}}_T(t)$ be given by (16), and let $\hat{\bar{S}}_T^*(t)$ be given by

$$\frac{d\hat{\bar{S}}_T^*(t)}{dt} = \bar{\alpha}_s^* - (\gamma_s + 1)\hat{\bar{S}}_T^*(t) \implies \hat{\bar{S}}_T^*(t) = \hat{\bar{S}}_T^*(0)e^{-(\gamma_s+1)t} + (1 - e^{-(\gamma_s+1)t})\bar{S}_T^* \tag{17}$$

where $\hat{\bar{S}}_T^*(0) = \hat{\bar{S}}_T(0) = \bar{S}_T(0) \leq \bar{S}_T^*$, which implies that $\hat{\bar{S}}_T^*(t) \leq \bar{S}_T^*, \forall t \geq 0$. Let $e_{s,T}(t) = \hat{\bar{S}}_T^*(t) - \hat{\bar{S}}_T(t)$ such that

$$\dot{e}_{s,T}(t) = \underbrace{\bar{\alpha}_s^* - \bar{\alpha}_s(t)}_{\geq 0, \forall t \geq 0, \text{ Assumption } 3} - (\gamma_s + 1)e_s(t),$$

which implies that $\mathbb{R}_+$ is a positively invariant set for the $e_{s,T}(t)$ dynamics. Since $e_{s,T}(0) = 0$, this implies that $e_{s,T}(t) \geq 0, \forall t \geq 0$ and thus $\bar{S}_T(t) = \hat{\bar{S}}_T(t) \leq \hat{\bar{S}}_T^*(t) \leq \bar{S}_T^*, \forall t \geq 0$ (first equality from Remark 5). From the positivity of $\bar{S}(t)$ and $\bar{c}_i(t), \forall i =, \dots, n$ (Corollary 1), we have that $\bar{S}(t) \leq \bar{S}_T(t) \leq \bar{S}_T^*, \forall t \geq 0$, thus proving (I). Similarly, $\hat{\bar{S}}(t) \leq \hat{\bar{S}}_T(t) \leq \bar{S}_T^*, \forall t \geq 0$, thus proving (II).

Let $\hat{\bar{E}}_{i,T}(t)$ be given by (16) and $\hat{\bar{E}}_i^*(t)$ be given by

$$\frac{d\hat{\bar{E}}_i^*(t)}{dt} = \bar{\alpha}_i^* - (\gamma_i + 1)\hat{\bar{E}}_i^*(t) \implies \hat{\bar{E}}_i^*(t) = \hat{\bar{E}}_i^*(0)e^{-(\gamma_i+1)t} + (1 - e^{-(\gamma_i+1)t})\bar{E}_{i,T}^*, \tag{18}$$

where $\hat{\bar{E}}_i^*(0) = \hat{\bar{E}}_{i,T}(0) = \bar{E}_{i,T}(0) \leq \bar{E}_{i,T}^*$, which implies that $\hat{\bar{E}}_i^*(t) \leq \bar{E}_{i,T}^*, \forall t \geq 0$. Let $e_{i,T}(t) = \hat{\bar{E}}_i^*(t) - \hat{\bar{E}}_{i,T}(t)$ and thus

$$\dot{e}_{i,T}(t) = \underbrace{\bar{\alpha}_i^* - \bar{\alpha}_i(t)}_{\geq 0, \forall t \geq 0, \text{ Assumption } 3} - (\gamma_i + 1)e_i(t),$$

which implies that $\mathbb{R}_+$ is a positively invariant set for the $e_{i,T}(t)$ dynamics. Since $e_{i,T}(0) = 0$, this implies that $e_{i,T}(t) \geq 0, \forall t \geq 0$ and thus $\bar{E}_{i,T}(t) = \hat{\bar{E}}_{i,T}(t) \leq \hat{\bar{E}}_i^*(t) \leq \bar{E}_{i,T}^*, \forall t \geq 0$ (first equality from Remark 5). From the positivity of $\bar{E}_i(t)$ and $\bar{c}_i(t)$ (Corollary 1), we have that $\bar{E}_i(t) \leq \bar{E}_{i,T}(t) \leq \bar{E}_{i,T}^*, \forall t \geq 0$, thus proving (III). By similar logic, we also have that $\hat{\bar{E}}_i(t) \leq \hat{\bar{E}}_{i,T}(t) \leq \bar{E}_{i,T}^*, \forall t \geq 0$, thus proving part of (IV).

Let $\hat{\bar{E}}_{i,*}(t)$ such that $\hat{\bar{E}}_{i,*}(0) = \hat{\bar{E}}_i(0)$ and

$$\frac{d\hat{\bar{E}}_{i,*}(t)}{dt} = \bar{\alpha}_{i,*} - (\gamma_i + 1)\hat{\bar{E}}_{i,*}(t) - \tilde{a}_i\theta_i^*\hat{\bar{E}}_{i,*}(t)\bar{S}_T^* \implies \hat{\bar{E}}_{i,*}(t) = \hat{\bar{E}}_{i,*}(0)e^{-(\gamma_i+1+\tilde{a}_i\bar{S}_T^*)t} + (1 - e^{-(\gamma_i+1+\tilde{a}_i\bar{S}_T^*)t})\bar{E}_{i,*}, \tag{19}$$

where $\hat{\bar{E}}_{i,*}(0) = \hat{\bar{E}}_i(0) = \bar{E}_i(0) \geq \bar{E}_{i,*}$, which implies that $\hat{\bar{E}}_{i,*}(t) \geq \bar{E}_{i,*}, \forall t \geq 0$. Let $e_i(t) = \hat{\bar{E}}_i(t) - \hat{\bar{E}}_{i,*}(t)$ such that

$$\dot{e}_i(t) = \underbrace{\bar{\alpha}_i(t) - \bar{\alpha}_{i,*}}_{\geq 0, \forall t \geq 0, \text{ Assumption } 3} - (\gamma_i + 1 + \tilde{a}_i\theta_i^*\bar{S}_T^*)e_i(t) + \tilde{a}_i\theta_i^*\hat{\bar{E}}_{i,*}(t)\underbrace{(\bar{S}_T^* - \hat{\bar{S}}(t))}_{\geq 0, \forall t \geq 0, \text{ by (II)}},$$

which implies that $\mathbb{R}_+$ is a positively invariant set for the $e_i(t)$ dynamics. Since $e_i(0) = 0$, this implies that $e_i(t) \geq 0, \forall t \geq 0$ and thus $\hat{\bar{E}}_{i,*}(t) \geq \bar{E}_{i,*}, \forall t \geq 0$. Thus proving (IV).

■

*Remark* 7. By Assumption 3, we have that $\bar{E}_{i,T}^*$ and $\bar{S}_T^*$ are independent of $\delta$ and thus these upper bounds for $\bar{E}_i(t)$ and $\bar{S}(t)$, respectively, are also independent $\delta$.

The following results demonstrates that Assumption 3 implies that the concentration of fixed species is localized at the region specified in Table 1. The parameter $\delta > 0$, controls the amount of localization.

**Proposition** 1. Consider the systems given by (8). Let $x_i^* \in (0, 1)$ for Case II and $x_s^* \in (0, 1)$ for Case III, be given by Table 1. Suppose that Assumption 3 holds for $x_i^*$, $x_s^*$, and a given $\delta > 0$. Let $E_{i,T}(t, x) = E_i(t, x) + c_i(t, x)$ and $S_T(t, x) = S(t, x) + \sum_{j=1}^n c_j(t, x)$. Then, for all $E_{i,T}(0, x) \leq \delta, \forall x \notin [x_i^* - \delta, x_i^* + \delta]$ for Case II, and $S_T(0, x) \leq \delta, \forall x \notin [x_i^* - \delta, x_i^* + \delta]$ for Case III, we have that

1. Case II: $E_i(t, x) \leq \delta, \forall x \notin [x_i^* - \delta, x_i^* + \delta], \quad \forall t \geq 0, \forall i = 1, \dots, n,$
2. Case III: $S(t, x) \leq \delta, \forall x \notin [x_s^* - \delta, x_s^* + \delta], \quad \forall t \geq 0,$

*Proof:* For Case II, $E_{i,T}(t, x)$ satisfies

$$\frac{\partial E_{i,T}(t, x)}{\partial t} = \alpha_i(t, x) - (\gamma_i + 1)E_{i,T}(t, x). \tag{20a}$$

For $c \notin [x_i^* - \delta, x_i^* + \delta]$ we have that $E_i(t,c) \leq E_{i,T}(t,c) \leq \frac{\alpha_i(t,c)}{\gamma_i+1} \leq \frac{1}{\gamma_i+1}\delta \leq \delta, \forall t \geq 0$. Similarly, for Case III, $S_T(t,x)$ satisfies

$$\frac{\partial S_T(t,x)}{\partial t} = \alpha_s(t,x) - (\gamma_s + 1)S_T(t,x). \tag{20b}$$

For $c \notin [x_s^* - \delta, x_s^* + \delta]$ we have that $S(t,c) \leq S_T(t,c) \leq \frac{\alpha_s(t,c)}{\gamma_s+1} \leq \frac{1}{\gamma_s+1}\delta \leq \delta, \forall t \geq 0$. ■

The following claim will aid us in rewriting (9) in the form of the reduced dynamics given by (10) with additional "disturbance" terms of order $\epsilon$ and $\delta$. The claim is written to handle Case I-III.

**Claim** 3. For a given $y_1(t,x) \in H$ and $y_2(t,x) \in H$, where $H = L_2(0,1)$, suppose that $y_1(t,x), y_2(t,x) \geq 0, \forall t \geq 0, \forall x \in [0,1]$ and that there exists $y_1^*, y_2^* \in \mathbb{R}_+$ such that $||y_1(t,x)||_H \leq y_1^*, \forall t \geq 0$ and $||y_2(t,x)||_H \leq y_2^*, \forall t \geq 0$, let $\bar{y}_1(t) = \int_0^1 y_1(t,x)dx \leq \bar{y}_1^*$ and $\bar{y}_2(t) = \int_0^1 y_2(t,x)dx \leq \bar{y}_2^*$. Let $\hat{v}_1(x)$ and $\hat{v}_2(x)$ be smooth positive functions and denote $y_1^\perp(t,x) = y_1(t,x) - \bar{y}_1(t)\hat{v}_1(x)$ and $y_2^\perp(t,x) = y_2(t,x) - \bar{y}_2(t)\hat{v}_2(x)$.

1. There exists constant $k_1, k_2 > 0$ such that

$$\int_0^1 y_1(t,x)y_2(t,x)dx = \bar{y}_1(t)\bar{y}_2(t)\theta + \Delta(t), \quad \forall t \geq 0$$

   where $\theta = \int_0^1 \hat{v}_1(x)\hat{v}_2(x)dx$ and $|\Delta(t)| \leq k_1||y_1^\perp(t,x)||_H + k_2||y_2^\perp(t,x)||_H$.

2. Suppose that for a given $x^* \in (0,1)$ and $\delta > 0$ such that $[x^* - \delta, x^* + \delta] \subset [0,1]$ we have that $y_2(t,x) \leq \delta, \forall x \notin [x^* - \delta, x^* + \delta]$. Furthermore assume that $\bar{y}_1^*$ and $\bar{y}_2^*$ are independent of $\delta$. Then there exists constant $k_3(\delta), k_4 > 0$ such that

$$\int_0^1 y_1(t,x)y_2(t,x)dx = \bar{y}_1(t)\bar{y}_2(t)\theta + \Delta(t), \quad \forall t \geq 0$$

   where $\theta = \hat{v}_1(x^*)$ and $|\Delta(t)| \leq k_3(\delta)||y_1^\perp(t,x)||_H + k_4\delta$.

*Proof:* To proof the first claim, notice that

$$\begin{aligned}
\int_0^1 y_1(t,x)y_2(t,x)dx &= \int_0^1 \big(\bar{y}_1(t)\hat{v}_1(x) + y_1^\perp(t,x)\big)\big(\bar{y}_2(t)\hat{v}_2(x) + y_2^\perp(t,x)\big)dx \\
&= \bar{y}_1(t)\bar{y}_2(t)\int_0^1 \hat{v}_1(x)\hat{v}_2(x)dx + \underbrace{\int_0^1 y_1^\perp(t,x)\big(\bar{y}_2(t)\hat{v}_2(x) + y_2^\perp(t,x)\big) + \bar{y}_1(t)\hat{v}_1(x)y_2^\perp(t,x)dx}_{\Delta(t)} \\
&= \bar{y}_1(t)\bar{y}_2(t)\theta + \Delta(t)
\end{aligned}$$

where $\theta = \int_0^1 \hat{v}_1(x)\hat{v}_2(x)dx$. Let $\hat{v}_1^* = \max_{x\in[0,1]}(\hat{v}_1(x))$ and leveraging the Cauchy-Schwarz inequality in $H$,

$$\begin{aligned}
\big|\Delta(t)\big| &= \left|\int_0^1 y_1^\perp(t,x)\big(\bar{y}_2(t)\hat{v}_2(x) + y_2^\perp(t,x)\big) + \bar{y}_1(t)\hat{v}_1(x)y_2^\perp(t,x)dx\right| \\
&= \left|\langle y_1^\perp(t,x), y_2(t,x)\rangle_H + \langle \bar{y}_1(t)\hat{v}_1(x), y_2^\perp(t,x)\rangle_H\right| \\
&\leq \left|\langle y_1^\perp(t,x), y_2(t,x)\rangle_H\right| + \left|\langle \bar{y}_1(t)\hat{v}_1(x), y_2^\perp(t,x)\rangle_H\right| \\
&\leq ||y_1^\perp(t,x)||_H||y_2(t,x)||_H + ||\bar{y}_1(t)\hat{v}_1(x)||_H||y_2^\perp(t,x)||_H \\
&\leq ||y_1^\perp(t,x)||_H y_2^* + \bar{y}_1^*\hat{v}_1^*||y_2^\perp(t,x)||_H,
\end{aligned}$$

thus $k_1 = y_2^*$ and $k_2 = \bar{y}_1^*\hat{v}_1^*$. For the second part of the claim, notice that

$$\bar{y}_2(t) = \int_0^{x^*-\delta} y_2(t,x)dx + \int_{x^*-\delta}^{x^*+\delta} y_2(t,x)dx + \int_{x^*+\delta}^1 y_2(t,x)dx = \int_{x^*-\delta}^{x^*+\delta} y_2(t,x)dx + \bar{y}_{2,\delta}(t)$$

where

$$\bar{y}_{2,\delta}(t) = \int_0^{x^*-\delta} y_2(t,x)dx + \int_{x^*+\delta}^1 y_2(t,x)dx \quad \text{and} \quad |\bar{y}_{2,\delta}(t)| \leq (x^* - \delta)\delta + (1 - x^* - \delta)\delta \leq 2\delta.$$

Next,

$$\begin{aligned}
\int_0^1 y_1(t,x)y_2(t,x)dx &= \int_0^1 \big(\bar{y}_1(t)\hat{v}_1(x) + y_1^{\perp}(t,x)\big)y_2(t,x)dx \\
&= \int_0^1 \bar{y}_1(t)\hat{v}_1(x)y_2(t,x)dx + \int_0^1 y_1^{\perp}(t,x)y_2(t,x)dx \\
&= \int_0^1 \bar{y}_1(t)\hat{v}_1(x)y_2(t,x)dx + \Delta_1(t) \\
&= \bar{y}_1(t)\big[\int_0^{x^*-\delta} \hat{v}_1(x)y_2(t,x)dx + \int_{x^*-\delta}^{x^*+\delta} \hat{v}_1(x)y_2(t,x)dx + \int_{x^*+\delta}^1 \hat{v}_1(x)y_2(t,x)dx\big] + \Delta_1(t) \\
&= \bar{y}_1(t)\int_{x^*-\delta}^{x^*+\delta} \hat{v}_1(x)y_2(t,x)dx + \Delta_2(t) + \Delta_1(t) \\
&= \bar{y}_1(t)\hat{v}_1(c)\int_{x^*-\delta}^{x^*+\delta} y_2(t,x)dx + \Delta_2(t) + \Delta_1(t), \text{ for some } c \in [x^*-\delta, x^*+\delta] \\
&= \bar{y}_1(t)\bar{y}_2(t)\hat{v}_1(c) - \bar{y}_1(t)\hat{v}_1(c)\bar{y}_{2,\delta}(t) + \Delta_2(t) + \Delta_1(t) \\
&= \bar{y}_1(t)\bar{y}_2(t)\hat{v}_1(c) + \Delta_3(t) + \Delta_2(t) + \Delta_1(t) \\
&= \bar{y}_1(t)\bar{y}_2(t)\hat{v}_1(x^*) + \Delta_4(t) + \Delta_3(t) + \Delta_2(t) + \Delta_1(t) \\
&= \bar{y}_1(t)\bar{y}_2(t)\theta + \Delta(t),
\end{aligned}$$

where $\theta = \hat{v}_1(x^*)$ and $\Delta(t) = \sum_{i=1}^4 \Delta_i(t)$, the existence of $c$ is guaranteed by the mean-value theorem for integrals [6]. Notice that

$$|\Delta_1(t)| = \Big|\int_0^1 y_1^{\perp}(t,x)y_2(t,x)dx\Big| = \big|\langle y_1^{\perp}(t,x), y_2(t,x)\rangle_H\big| \leq ||y_2(t,x)||_H||y_1^{\perp}(t,x)||_H \leq y_2^*||y_1^{\perp}(t,x)||_H,$$

$$\begin{aligned}
|\Delta_2(t)| &= \Big|\bar{y}_1(t)\big[\int_0^{x^*-\delta} \hat{v}_1(x)y_2(t,x)dx + \int_{x^*+\delta}^1 \hat{v}_1(x)y_2(t,x)dx\big]\Big| \\
&\leq y_1^*\hat{v}_1^*\Big|\int_0^{x^*-\delta} y_2(t,x)dx + \int_{x^*+\delta}^1 y_2(t,x)dx\Big| \\
&= \bar{y}_1^*\hat{v}_1^*\Big|\bar{y}_{2,\delta}(t)\Big| \\
&\leq 2\bar{y}_1^*\hat{v}_1^*\delta
\end{aligned}$$

$$|\Delta_3(t)| = |\bar{y}_1(t)\hat{v}_1(c)\bar{y}_{2,\delta}(t)| \leq 2\bar{y}_1^*\hat{v}_1^*\delta,$$

the following uses the smoothness $\hat{v}_1(x)$ to guarantee uniform continuity i.e., the existence of $L_v > 0$ such that for all $x_1, x_2 \in (0,1)$, we have that $|\hat{v}_1(x_2) - \hat{v}_1(x_1)| \leq L_v|x_2 - x_1|$, and hence

$$|\Delta_4(t)| = \big|\bar{y}_1(t)\bar{y}_2(t)(\hat{v}_1(c) - \hat{v}_1(x^*))\big| \leq \bar{y}_1^*\bar{y}_2^*\big|\hat{v}_1(c) - \hat{v}_1(x^*)\big| \leq 2\bar{y}_1^*\bar{y}_2^*L_v\delta.$$

Finally,

$$\big|\Delta(t)\big| = \Big|\sum_{i=1}^4 \Delta_i(t)\Big| \leq \sum_{i=1}^4 \Big|\Delta_i(t)\Big| \leq k_3||y_1^{\perp}(t,x)||_H + k_4\delta,$$

where $k_3 = y_2^*$ and $k_4 = 2\bar{y}_1^*(2\hat{v}_1^* + \bar{y}_2^*L_v)$. ■

*Remark* 8. In the proof of Claim 3-2, $y_2^*$ may depend on $\delta$ since we assume that $\bar{y}_2^*$ is independent of $\delta$ (one expects that $y_2^*$ increases with decreasing $\delta$), thus $k_3$ may depend on $\delta$.

**Corollary** 2. *Consider the systems given by* (8)*. The assumptions necessary to apply Claim* 3 *to*

$$\int_0^1 E_i(t,x)S(t,x)dx,$$

*are satisfied by Claim 1, Proposition 1, and Claim 2 (along with Remark 7). Furthermore, considering the results from Lemma 4, we are guaranteed he existence of* $L_i > 0$ *for* $i = 1, \ldots, 5$ *such that for all* $\epsilon > 0$

$$\int_0^1 E_i(t,x)S(t,x)dx = \bar{E}_i(t)\bar{S}(t)\theta_i^* + \Delta_i(t), \quad \forall t \geq 0,$$

*where* $\theta_i^*$ *is given by* (10b) *for Cases I-III (as in Table 1), and*

$$|\Delta_i(t)| \leq \begin{cases} L_1\epsilon + L_2 e^{-\zeta t/\epsilon} & \text{for Case I} \\ L_3(\delta)\epsilon + L_4(\delta)e^{-\zeta t/\epsilon} + L_5\delta & \text{for Case II and Case III} \end{cases}, \tag{21}$$

*where* $\zeta$ *is as in Lemma 4. The coefficients* $L_3$ *and* $L_4$ *may depend on* $\delta$ *by the discussion in Remark 4 and Remark 8.*

Let $\bar{\boldsymbol{c}}(t) = [\bar{c}_1(t), \ldots, \bar{c}_n(t)]^T$ where $\hat{\bar{c}}_i(t)$ is given by (9) , $\bar{\boldsymbol{E}}_T(t) = [\bar{E}_{1,T}(t), \ldots, \bar{E}_{1,T}(t)]^T$ where $\bar{E}_{i,T}(t)$ is given by (16), $\hat{\bar{\boldsymbol{c}}}(t) = [\hat{\bar{c}}_1(t), \ldots, \hat{\bar{c}}_n(t)]^T$, where $\hat{\bar{c}}_i(t)$ is given by (10), the $\bar{\boldsymbol{c}}(t)$ and $\hat{\bar{\boldsymbol{c}}}(t)$ dynamics may be written as

$$\frac{d\bar{\boldsymbol{c}}(t)}{dt} = \boldsymbol{f}_c(\bar{\boldsymbol{E}}_T, \bar{S}_T, \bar{\boldsymbol{c}}) + \boldsymbol{\Delta}(t), \tag{22a}$$

$$\frac{d\hat{\bar{\boldsymbol{c}}}(t)}{dt} = \boldsymbol{f}_c(\bar{\boldsymbol{E}}_T, \bar{S}_T, \hat{\bar{\boldsymbol{c}}}), \tag{22b}$$

where $\boldsymbol{\Delta}^i = \Delta_i(t)$ as in Corollary 2 and $\boldsymbol{f}_c : \mathbb{R}^n \times \mathbb{R} \times \mathbb{R}^n \to \mathbb{R}$ is given by

$$\boldsymbol{f}_c^i(\bar{\boldsymbol{E}}_T, \bar{S}_T, \hat{\bar{\boldsymbol{c}}}) = \frac{1}{\eta_i}\left[\left(\bar{\boldsymbol{E}}_T^i(t) - \hat{\bar{\boldsymbol{c}}}^i(t)\right)\left(\bar{S}_T(t) - \sum_{j=1}^n \hat{\bar{\boldsymbol{c}}}^j(t)\right)\frac{\tilde{a}_i\theta_i^*}{\tilde{d}_i} - \hat{\bar{\boldsymbol{c}}}^i(t)\right] = \frac{1}{\eta_i}\left[\hat{\bar{E}}_i(t)\hat{\bar{S}}(t)\frac{\tilde{a}_i\theta_i^*}{\tilde{d}_i} - \hat{\bar{\boldsymbol{c}}}^i(t)\right],$$

we used the fact that $\bar{E}_{i,T}(t) = \hat{\bar{E}}_{i,T}(t)$ and $\bar{S}_T(t) = \hat{\bar{S}}_T(t)$ (Remark 5). By the form of (22), it is clear that the $\bar{\boldsymbol{c}}(t)$ dynamics (22a) are in the form the $\hat{\bar{\boldsymbol{c}}}(t)$ (22b) with additional "perturbation" terms of order $\epsilon$ and $\delta$. The variables $\bar{\boldsymbol{E}}_T(t)$, $\bar{S}_T(t)$, and $\bar{\boldsymbol{c}}(t)$ are enough to fully describe (9) and $\bar{\boldsymbol{E}}_T(t)$, $\bar{S}_T(t)$, and $\hat{\bar{\boldsymbol{c}}}(t)$ are enough to fully describe (10). Notice that

$$\frac{\partial \boldsymbol{f}_c^i(\bar{\boldsymbol{E}}_T, \bar{S}_T, \hat{\bar{\boldsymbol{c}}})}{\partial \bar{\boldsymbol{E}}_T^i} = \frac{1}{\eta_i}\hat{\bar{S}}(t)\frac{\tilde{a}_i\theta_i^*}{\tilde{d}_i}, \quad \frac{\partial \boldsymbol{f}_c^i(\bar{\boldsymbol{E}}_T, \bar{S}_T, \hat{\bar{\boldsymbol{c}}})}{\partial \bar{\boldsymbol{E}}_T^j} = 0, \quad \frac{\partial \boldsymbol{f}_c^i(\bar{\boldsymbol{E}}_T, \bar{S}_T, \hat{\bar{\boldsymbol{c}}})}{\partial \bar{\boldsymbol{S}}_T} = \frac{1}{\eta_i}\hat{\bar{E}}_i(t)\frac{\tilde{a}_i\theta_i^*}{\tilde{d}_i}, \tag{23}$$

and by Claim 2 these terms are uniformly bounded in time and for $i = 1, \ldots, n$. Considering Lemma 2 with $\boldsymbol{z}_1(t) = [\bar{\boldsymbol{E}}_T^T(t), \bar{S}_T(t)]^T$ and $\boldsymbol{z}_2(t) = \hat{\bar{\boldsymbol{c}}}(t)$, then condition (I) is satisfied by the discussion in Remark 6 and condition (III) is satisfied by (23), thus we can treat $\bar{\boldsymbol{E}}_T$ and $\bar{S}_T(t)$ as time varying inputs to (22b) when showing that (II) is satisfied.

We now show that the dynamics (22b) are contracting and thus we can apply the robustness property of contracting systems (Lemma 3) to show that the solutions of (22b) and (22a) are close.

**Lemma 5.** *Consider the system (8) and let* $\epsilon > 0$ *be defined for Case I-III by Table 1. Let* $\hat{\bar{\boldsymbol{c}}}(t)$ *be given by (22b) and* $\bar{\boldsymbol{c}}(t)$ *be given by (22a). Suppose the conditions of Claim 1, Lemma 4, and Claim 2 hold. Then, there exists* $L_{c,1}, \epsilon^* > 0$*, such that for all* $\epsilon \leq \epsilon^*$*, the solutions* $\hat{\bar{\boldsymbol{c}}}(t)$ *and* $\bar{\boldsymbol{c}}(t)$ *satisfy*

$$|\hat{\bar{\boldsymbol{c}}}(t) - \bar{\boldsymbol{c}}(t)| \leq |\Delta_c|, \quad \forall t \geq 0, \tag{24}$$

*where* $|\Delta_c| = L_{c,1}\epsilon$ *for Case I. For Cases II-III, if in addition, the conditions of Proposition 1 hold for all* $0 \leq \delta \leq \delta^*$*, there exists* $L_{c,3} > 0$ *such that for all* $0 < \delta < \delta^*$*, there exists* $L_{c,2}(\delta)$ *such that (24) is satisfied with* $|\Delta_c| = L_{c,2}(\delta)\epsilon + L_{c,3}\delta$*.*

*Proof:* Consider the metric

$$\boldsymbol{P}(t) = \mathrm{diag}([\frac{1}{\tilde{a}_1\theta_1^*\hat{\bar{E}}_1(t)}, \ldots, \frac{1}{\tilde{a}_n\theta_n^*\hat{\bar{E}}_n(t)}]^T) \tag{25}$$

where $\hat{\bar{E}}_i(t)$ is given by (10). The total time derivative of $\boldsymbol{P}$ is given by

$$\dot{\boldsymbol{P}}(t) = -\boldsymbol{P}(t)\mathrm{diag}(\boldsymbol{v}_p), \quad \text{where} \quad \boldsymbol{v}_p^i = \frac{d\hat{\bar{E}}_i(t)}{dt}/\hat{\bar{E}}_i(t) = \bar{\alpha}_i(t)/\hat{\bar{E}}_i(t) - (\gamma_i+1) - \tilde{a}_i\theta_i^*\hat{\bar{S}}(t) + (\gamma_s + k_i + d_i)\frac{\hat{\bar{c}}_i(t)}{\hat{\bar{E}}_i(t)}.$$

The Jacobian of (22b) is given by

$$\frac{\partial \boldsymbol{f}_c}{\partial \hat{\bar{\boldsymbol{c}}}} = -\text{diag}(\boldsymbol{v}_c) + \boldsymbol{\sigma}, \qquad \text{where} \qquad \boldsymbol{v}_c^i = \tilde{a}_i \theta_i^* \hat{\bar{S}}(t) + \tilde{d}_i,$$

and $\boldsymbol{\sigma}$ is a rank one matrix given by

$$\boldsymbol{\sigma} = [\tilde{a}_1 \theta_1^* \hat{\bar{E}}_1(t), \ldots, \tilde{a}_n \theta_n^* \hat{\bar{E}}_n(t)]^T [-1, \ldots, -1].$$

With the chosen metric $\boldsymbol{P}(t)$, we have that

$$\boldsymbol{P}\boldsymbol{\sigma} = -\underbrace{[1, \ldots, 1]^T [1, \ldots, 1]}_{\boldsymbol{\sigma}_1} \implies -n\boldsymbol{I}_{n,n} \leq \boldsymbol{\sigma}_1 \leq 0\boldsymbol{I}_{n,n}$$

since the symmetric rank one matrix $\boldsymbol{\sigma}_1$, has $n-1$ zero eigenvalues and the nontrivial eigenvalue is $\lambda_\sigma = -n$ for eigenvector $v_n = [1, \ldots, 1]^T$. Recalling the positivity of $\hat{\bar{E}}_i(t)$, $\hat{\bar{c}}_i(t)$, $\hat{\bar{S}}(t)$ (Claim 1), and $\bar{\alpha}_i(t)$, we have that

$$\begin{aligned} 1/2\left(\boldsymbol{P}(t)\frac{\partial \boldsymbol{f}_c}{\partial \boldsymbol{c}} + \frac{\partial \boldsymbol{f}_c^T}{\partial \boldsymbol{c}}\boldsymbol{P}(t) + \dot{\boldsymbol{P}}(t)\right) &= -\boldsymbol{P}\text{diag}(1/2\boldsymbol{v}_p + \boldsymbol{v}_c) + \boldsymbol{P}\boldsymbol{\sigma} \\ &\leq -\boldsymbol{P}\text{diag}(1/2\boldsymbol{v}_p + \boldsymbol{v}_c) \\ &\leq -\min_{i=1,\ldots,n}\left(1/2(\gamma_i + 1) + \gamma_s + \kappa_i + d_i\right)\boldsymbol{P} \\ &= -\xi\boldsymbol{P}. \end{aligned}$$

By Theorem 1, the system (22b) is contracting with contraction rate $\xi = 1/2 + \gamma_s + \min_{i=1,\ldots,n}\Big(1/2\gamma_i + \kappa_i + d_i\Big)$. Assumptions 2, 3 imply that (21) holds for $\Delta_i(t)$ in (22a). Therefore, for a given $\delta > 0$, we apply the result from Lemma 3 to the nominal system (22b) and the perturbed system (22a). Let $\epsilon^* = \zeta/(2\xi)$ and recalling that $\bar{c}_i(0) = \hat{\bar{c}}_i(0)$, then by Lemma 3, there exists $l_1, l_2(\delta), l_3 > 0$ such that for all $\epsilon < \epsilon^*$

$$|\bar{c}_i(t) - \hat{\bar{c}}_i(t)| \leq M l_1 \epsilon, \quad \forall t \geq 0, \text{ for Case I} \quad |\bar{c}_i(t) - \hat{\bar{c}}_i(t)| \leq M(l_2(\delta)\epsilon + l_3\delta), \quad \forall t \geq 0, \text{ for Case II-III},$$

where $M$ is a constant upper bound on the square root of the ratio of the biggest and smallest eigenvalues of $\boldsymbol{P}(t)$. Thus $L_{c,1} = Ml_1$, $L_{c,2}(\delta) = Ml_2(\delta)$, and $L_{c,3} = Ml_3$. We now show that $M$ exists. Let $r(t)$ be the ratio of the biggest and smallest eigenvalues of $\boldsymbol{P}(t)$. By Claim 2, there exists $\bar{E}_{i,*}, \bar{E}_{i,T}^* \in \mathbb{R}_+$ independent of $\epsilon$ and $\delta$ such that $\bar{E}_{i,*} \leq \hat{\bar{E}}_i(t) \leq \bar{E}_{i,T}^*, \forall t \geq 0$, and thus

$$r(t) = \frac{\max_{i=1,\ldots,n} \tilde{a}_i \theta_i^* \hat{\bar{E}}_i(t)}{\min_{i=1,\ldots,n} \tilde{a}_i \theta_i^* \hat{\bar{E}}_i(t)} \leq \frac{\max_{i=1,\ldots,n} \tilde{a}_i \theta_i^* \bar{E}_{i,T}^*}{\min_{i=1,\ldots,n} \tilde{a}_i \theta_i^* \bar{E}_{i,*}} \implies M^2 = \frac{\max_{i=1,\ldots,n} \tilde{a}_i \theta_i^* \bar{E}_{i,T}^*}{\min_{i=1,\ldots,n} \tilde{a}_i \theta_i^* \bar{E}_{i,*}}.$$

■

**Corollary** 3. *Recall Definition 5 and Remark 5, we have that*

$$|\bar{E}_i(t) - \hat{\bar{E}}_i(t)| = |\bar{E}_{i,T}(t) - \bar{c}_i(t) - (\hat{\bar{E}}_{i,T}(t) - \hat{\bar{c}}_i(t))| = |\hat{\bar{c}}_i(t) - \bar{c}_i(t)| \leq |\Delta_c|$$

*and*

$$|\bar{S}(t) - \hat{\bar{S}}(t)| = |\bar{S}_T(t) - \sum_{j=1}^n \bar{c}_j(t) - (\hat{\bar{S}}_T(t) - \sum_{j=1}^n \hat{\bar{c}}_j(t))| = |\sum_{j=1}^n \hat{\bar{c}}_j(t) - \bar{c}_j(t)| \leq \sum_{j=1}^n |\hat{\bar{c}}_j(t) - \bar{c}_j(t)| \leq n|\Delta_c|$$

*where $|\Delta_c|$ is as in Lemma 5. Thus, the quantity $|\Delta_z|$ as claimed to exist in Theorem 3, may be given by $|\Delta_z| = |\Delta_c|\sqrt{2 + n^2}$.*

*Remark* 9. We now comment on the sets $\Omega_z \in \mathbb{R}^{n+1}$ and $\Omega_{\bar{z}} \in \mathbb{R}^{n+1}$ as claimed to exist in Theorem 3. Let $\chi$ be as in Claim 1.

- For Cases I-III we require that $\boldsymbol{z}(0,x) \in \chi, \forall x \in [0,1]$ for Lemma 4 to hold. In Case I, $\Omega_z \in \mathbb{R}^{n+1} = \chi$. In Case II, for Proposition 1 to hold we also require that for $i = 1\ldots,n$, that $E_i(0,x)+c_i(0,x) \leq \delta, \forall x \notin [x_i^* - \delta, x_i^* + \delta]$, thus $\Omega_z$ is the intersection between the set that satisfies this condition and $\chi$. In Case III, for Proposition 1 to hold we also require that $S(0,x) + \sum_{j=1}^n c_j(0,x) \leq \delta, \forall x \notin [x_i^* - \delta, x_i^* + \delta]$, thus $\Omega_z$ is the intersection between the set that satisfies this condition and $\chi$. As discussed in Remark 4, $\chi$ may depend on $\delta$.
- For Claim 2 to hold, we assumed that $\bar{E}_{i,*} \leq \bar{E}_i(0)$, $\bar{E}_i(0)+\bar{c}_i(0) \leq \bar{E}^*_{i,T}$, and that $\bar{S}(0)+\sum_{j=1}^n \bar{c}_j(0) \leq \bar{S}^*_T$, thus $\Omega_{\bar{z}}$ is the set that satisfies these conditions.

### 1.4 Fast Diffusion and Bingding Dynamics

The approximation result of Theorem 3, holds well if $\boldsymbol{w}^\perp$ is small, where $\boldsymbol{w}^\perp$ is given by Definition 4. This was guaranteed by Lemma 4 which is based on Theorem 2. The proof of Theorem 2 was based on the principle that the $\frac{1}{\epsilon}\boldsymbol{D}\boldsymbol{\mathcal{L}}_{\boldsymbol{v}}(\boldsymbol{z}_d^\perp)$ term dominates the $\boldsymbol{f}_d^\perp(t,x,\boldsymbol{z}_s,\boldsymbol{z}_d)$ term in (7c) and thus all solutions converged to $z_d^\perp \to 0$, the quasi-steady state of $\frac{1}{\epsilon}\boldsymbol{D}\boldsymbol{\mathcal{L}}_{\boldsymbol{v}}(\boldsymbol{z}_d^\perp)$. However, for (8), if $\epsilon$ and $\eta_i$ are of similar order of magnitude, the corresponding term in $\boldsymbol{f}_d^\perp(t,x,\boldsymbol{z}_s,\boldsymbol{z}_d)$ may be comparable to $\frac{1}{\epsilon}\boldsymbol{D}\boldsymbol{\mathcal{L}}_{\boldsymbol{v}}(\boldsymbol{z}_d^\perp)$ and cannot be neglected. The terms $\epsilon$ and $\eta_i$ being comparable corresponds to diffusion and the binding between $\mathrm{E_i}$ and S occurring at similar timescales, which often time occurs within the cell [7]. Here we show that when both diffusion and the binding dynamics dominate in (8), $z_d^\perp = 0$ is still the quasi-steady state, that is, all freely diffusing species mirror their available volume profile.

When both diffusion and the binding dynamics dominate in (8) we have that

$$\begin{aligned}
\frac{\partial E_i(t,x)}{\partial t} &= \underbrace{\frac{1}{\eta_i}\left[-\eta_i\frac{d}{dx}\big[J(x,E_i)\big] - E_i(t,x)S(t,x)\frac{\tilde{a}_i}{\tilde{d}_i} + c_i(t,x)\right]}_{\text{fast}} + \alpha_i(t,x) \\
&\qquad\qquad - (\gamma_i+1)(E_i(t,x)+c_i(t,x)), \\
\frac{\partial c_i(t,x)}{\partial t} &= \underbrace{\frac{1}{\eta_i}\left[-\eta_i\frac{d}{dx}\big[J(x,c_i)\big] + E_i(t,x)S(t,x)\frac{\tilde{a}_i}{\tilde{d}_i} - c_i(t,x)\right]}_{\text{fast}}, \\
\frac{\partial S(t,x)}{\partial t} &= \underbrace{\frac{1}{\eta_1}\left[-\eta_1\frac{d}{dx}\big[J(x,S)\big] + \sum_{j=1}^n \frac{\eta_1}{\eta_j}(-E_j(t,x)S(t,x)\frac{\tilde{a}_j}{\tilde{d}_j} + c_j(t,x))\right]}_{\text{fast}} + \alpha_s(t,x) \\
&\qquad\qquad - (\gamma_s+1)(S(t,x)+\sum_{j=1}^n c_j(t,x)).
\end{aligned} \tag{26}$$

We compute the quasi-steady state of the "fast dynamics in (26) for Cases I-III, that is $E_i(t,x)$, $c_i(t,x)$, and $S(t,x)$ such that

$$\begin{aligned}
0 &= \frac{1}{\eta_i}\left[-\eta_i\frac{d}{dx}\big[J(x,E_i)\big] - E_i(t,x)S(t,x)\frac{\tilde{a}_i}{\tilde{d}_i} + c_i(t,x)\right], \\
0 &= \frac{1}{\eta_i}\left[-\eta_i\frac{d}{dx}\big[J(x,c_i)\big] + E_i(t,x)S(t,x)\frac{\tilde{a}_i}{\tilde{d}_i} - c_i(t,x)\right], \\
0 &= \frac{1}{\eta_1}\left[-\eta_1\frac{d}{dx}\big[J(x,S)\big] + \sum_{j=1}^n \frac{\eta_1}{\eta_j}(-E_j(t,x)S(t,x)\frac{\tilde{a}_j}{\tilde{d}_j} + c_j(t,x))\right].
\end{aligned} \tag{27}$$

**Case I**: In this case, (27) is satisfied for

$$E_i(t,x) = \bar{E}_i(t)\hat{v}_{E_i}(x), \quad S(t,x) = \bar{S}(t)\hat{v}_S(x), \quad c_i(t,x) = \bar{c}_i(t)\hat{v}_{c_i}(x),$$

with the additional constraint that

$$E_i(t,x)S(t,x)\frac{\tilde{a}_i}{\tilde{d}_i} + c_i(t,x) = 0 \implies \bar{E}_i(t)\hat{v}_{E_i}(x)\bar{S}(t)\hat{v}_S(x)\frac{\tilde{a}_i}{\tilde{d}_i} + \bar{c}_i(t)\hat{v}_{c_i}(x) = 0, \forall x \in [0,1], \tag{28}$$

which in general (28) is a stringent condition since it is required to hold for all $x \in [0,1]$. However, by the key fact that $v_{c_i}(x) = v_{E_i}(x)v_S(x)$ (Main Text Equation 10), (28) is satisfied for all $x \in [0,1]$ by

$$\bar{c}_i(t) = \bar{E}_i(t)\bar{S}(t)\frac{\tilde{a}_i}{\tilde{d}_i}\theta_i^*, \quad \text{where} \quad \theta_i^* = \frac{\int_0^1 v_{E_i}(x)v_S(x)dx}{\left[\int_0^1 v_{E_i}(x)dx\right]\left[\int_0^1 v_S(x)dx\right]}.$$

**Case II and III**: For this cases, (27) is satisfied for

$$\text{Case II:} \quad S(t,x) = \bar{S}(t)\hat{v}_S(x), \quad \text{Case III:} \quad E_i(t,x) = \bar{E}_i(t)\hat{v}_{E_i}(x),$$

with the additional constraint that $E_i(t,x)S(t,x)\frac{\tilde{a}_i}{\tilde{d}_i} + c_i(t,x) = 0$ which implies that

$$\text{Case II:} \quad c_i(t,x) = E_i(t,x)\bar{S}(t)\hat{v}_S(x)\frac{\tilde{a}_i}{\tilde{d}_i}, \quad \text{Case III:} \quad E_i(t,x) = \bar{E}_i(t)\hat{v}_{E_i}(x)S(t,x)\frac{\tilde{a}_i}{\tilde{d}_i}. \tag{29}$$

Thus, when both diffusion and the binding dynamics dominate, the quasi-steady states are still those that correspond to freely diffusing species converging to their available volume profile. We observed that for Case I, this was possible by the fact that $v_{c_i}(x) = v_{E_i}(x)v_S(x)$. In a future study it should be shown that this quasi-steady state is a stable solution of the fast dynamics in (26).

# 2 Available Volume Profiles and Bounds on $\theta_i^*$

Following [8], we introduce a model of the available volume profiles of a freely diffusing species within the DNA mesh of the cell. Let $\rho(x)$ be the local density of DNA length such that $\int_0^1 \rho(x)dx = \bar{\rho} = \frac{L_p}{V_p}$, where $L_p$ is the total length of chromosome DNA, $V_p$ the volume where the DNA polymer is confined, and let $\hat{\rho}(x) = \frac{\rho(x)}{2\bar{\rho}}$. For a species diffusing inside the cell with radius of gyration $r$, we model the available volume profile $v(x)$ as:

$$v(x) = e^{-\kappa\pi r^2 2\bar{\rho}\hat{\rho}(x)} = e^{-(r/r^*)^2\hat{\rho}(x)}, \tag{30}$$

where $\kappa$ is an empirical coefficient (as discussed in [8]) and $r^{*2} = 1/(2\kappa\pi\bar{\rho})$. From the parameter values in [8], $r^* \approx 23$ nm. As commented in SI Section 7, $r^*$ can be estimated for a given context (e.g., growth conditions and strain ) by analyzing the concentration profile inside the cell of a freely diffusing species with a known radius of gyration, which is possible via superresolution imaging [9].

As shown in Figure A, we estimate the chromosome density as a step function.

$$\hat{\rho}(x) = \begin{cases} \frac{1}{2(1-\Delta x)}, & x \in [0, 1-\Delta x] \\ 0, & x \in (1-\Delta x, 1] \end{cases} \implies \bar{v} = \int_0^1 v(x)dx = v_0(1-\Delta x) + \Delta x, \tag{31}$$

where $v_0 = e^{-\frac{(r/r^*)^2}{2(1-\Delta x)}}$ and $\Delta x$ is the distance between the end of the chromosome and the cell poles (see Figure 1 in the Main Text). Its clear now, that our choice to define $\hat{\rho}(x) = \frac{\rho(x)}{2\bar{\rho}}$ was motivated by the fact that when $\Delta_x = 1/2$ (nucleoid evenly spread out between mid-cell and the halfway point between mid-cell and the cell poles), we have the convenient expressions $\hat{\rho}(0) = 1$ and $v_0 = e^{-(r/r^*)^2}$. Notice that $v_0 \to 0$ as $(r/r^*)^2 \to \infty$. Thus,

$$\hat{v}(x) = \frac{v(x)}{\bar{v}(x)} = \begin{cases} 0 \le \frac{v_0}{v_0(1-\Delta x)+\Delta x} \le 1, & x \in [0, 1-\Delta x] \\ 1 \le \frac{1}{v_0(1-\Delta x)+\Delta x} \le \frac{1}{\Delta x}, & x \in (1-\Delta x, 1] \end{cases}$$

**Bounds on $\theta_i^*$** : For the species $\mathrm{E_i}$ and S as described in the Main Text with radius of gyration $r_{e,i}$ and $r_s$, respectively. The available volume profiles are given by

$$v_{E_i}(x) = e^{-(r_{e,i}/r^*)^2\hat{\rho}(x)} \quad \text{and} \quad v_S(x) = e^{-(r_s/r^*)^2\hat{\rho}(x)},$$

respectively, We summarize the bounds on $\theta_i^*$ (Main Text Equation 15), assuming $\rho(x)$ is a step function as above. Let

$$v_{0,E_i} = v_{E_i}(0) = e^{-\frac{(r_{e,i}/r^*)^2}{2(1-\Delta x)}}, \quad \text{and} \quad v_{0,S} = v_S(0) = e^{-\frac{(r_s/r^*)^2}{2(1-\Delta x)}}, \tag{32}$$

and Case I-III (as in the Main Text, Equation 15) in the Main Text, the bounds on $\theta_i^*$ are given by

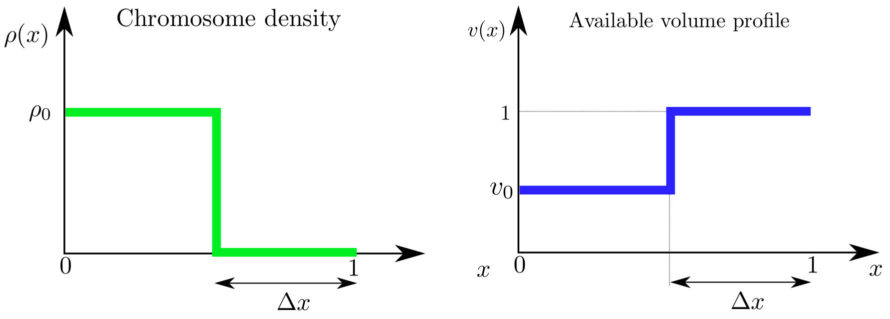


Figure A: **Idealization of the chromosome density which yields a simple estimate of the available volume profile.** The chromosome density is approximated as a step function implying by (30) that the available volume profile is also a step function. Here $\Delta x$ is the distance between the end of the chromosome and the cell poles.

- For **Case I**, this idealization implies that

$$\theta_i^* = \frac{v_{0,E_i} v_{0,S}(1-\Delta x) + \Delta x}{\left[v_{0,E_i}(1-\Delta x)+\Delta x\right]\left[v_{0,S}(1-\Delta x)+\Delta x\right]} \implies 1 \leq \theta_i \leq \frac{1}{\Delta x} \tag{33a}$$

The upper limit of $\theta_i^*$ is $\frac{1}{\Delta x}$ and is reached as $v_{0,E_i}$ and $v_{0,S}$ approach zero, which occurs as $r_{e,i}/r^* \to \infty$ and $r_s/r^* \to \infty$. The lower limit of $\theta_i^*$ is unity and is achieved if $v_{0,E_i}$ or $v_{0,S}$ approach one, which occurs if any of the two species is sufficiently small ($r_{e,i}/r^* \ll 1$ or $r_s/r^* \ll 1$ ). Since $\theta_i^* \geq 1$, it implies that the binding between $\mathrm{E_i}$ and S is always equal to or greater than that predicted by a well-mixed model (Main Text Equation 9).

- For **Case II-III**, let $x_i^*$ and $x_s^*$ as in Assumption 3 in the Main Text and thus

$$a_i = \begin{cases} v_{0,S}, & \text{Case II} \\ v_{0,E_i}, & \text{Case III} \end{cases}, \tag{33b}$$

$$\theta_i^* = \begin{cases} 0 \leq \dfrac{a_i}{a_i(1-\Delta x)+\Delta x} \leq 1, & x_i^* \leq 1-\Delta x \text{ for Case II and } x_s^* \leq 1-\Delta x \text{ for Case III} \\ 1 \leq \dfrac{1}{a_i(1-\Delta x)+\Delta x} \leq \dfrac{1}{\Delta x}. & x_i^* > 1-\Delta x \text{ for Case II and } x_s^* > 1-\Delta x \text{ for Case III} \end{cases} \tag{33c}$$

When $x_i^* \leq 1-\Delta x$ for Case II and $x_s^* \leq 1-\Delta x$ for Case III, we have that $\theta_i^* \leq 1$, the lower limit $\theta_i^* = 0$ is achieved when $r_e/r^* \to \infty$ for Case II ($r_s/r^* \to \infty$ for Case III), the upper limit $\theta_i^* = 1$ is achieved when $r_e/r^* \to 0$ for Case II ($r_s/r^* \to 0$ for Case III). When $x_i^* > 1-\Delta x$ for Case II and $x_s^* > 1-\Delta x$ for Case III, we have that $\theta_i^* \geq 1$, the lower limit $\theta_i^* = 1$ is achieved when $r_e/r^* \to 0$ for Case II ($r_s/r^* \to \infty$ for Case III), the upper limit $\theta_i^* = 1/\Delta x$ is achieved when $r_e/r^* \to \infty$ for Case II ($r_s/r^* \to \infty$ for Case III).

# 3 Protein Production: Transcription and Translation

We consider gene ($D$) being transcribed by RNAP ($S$) to form a DNA-RNAP complex ($c_s$) to produce mRNA ($m$) which is translated by ribosomes ($R$) to form mRNA-ribosome complex ($c_m$) which produces protein $P$. The mRNA's degrade at rate $\gamma$. The RNAP, and ribosomes are produced at rates $\alpha_s(t,x)$ and $\alpha_r(t,x)$, respectively. We assume all species dilute at rate $\mu$, the cells growth rate. The corresponding biochemical reactions are:

$$\underbrace{\mathrm{D+S \underset{d_s}{\overset{a_s}{\rightleftharpoons}} c_s \xrightarrow{\kappa_s} m+S+D}}_{\text{transcription, case II}}, \quad \underbrace{\mathrm{m+R \underset{d_m}{\overset{a_m}{\rightleftharpoons}} c_m \xrightarrow{\kappa_m} P+R+m}}_{\text{translation, case I}}$$

$$\emptyset \xrightarrow{\alpha_s} \mathrm{S}, \quad \emptyset \xrightarrow{\alpha_r} \mathrm{R}, \quad \mathrm{m} \xrightarrow{\gamma_i} \emptyset, \quad \mathrm{C_m} \xrightarrow{\gamma_i} \mathrm{R},$$

where $\gamma$ is the mRNA degradation rate, $a_s$ and $d_s$ are the association and dissociation rate constants, respectively, between RNAP and the gene D, $\kappa_s$ is the catalytic rate of formation of mRNA m, $a_m$ and $d_m$ are the association and dissociation rate constants, respectively, between ribosomes and mRNA , $\kappa_m$ is the catalytic rate of formation of protein P. We assume that the total concentration of D is conserved, so that $D_T(x) = D(t,x) + c_s(t,x)$ and that $D_T(x)$ is localized at $x = x^*$.

**Spatial-temporal Dynamics**: The dynamics corresponding to these biochemical reactions are given by:

$$\frac{\partial c_s(t,x)}{\partial t} = a_s S(t,x) D(t,x) - (d_s + \kappa_s) c_s(t,x) - c_s(t,x), \tag{34a}$$

$$\frac{\partial S(t,x)}{\partial t} = \mathcal{L}_s(S) + \alpha_s(t,x) + \big[ - a_{s,i} D(t,x) S(t,x) + (d_s + \kappa_s) c_s(t,x) \big] - S(t,x), \tag{34b}$$

$$\frac{\partial m(t,x)}{\partial t} = \mathcal{L}_m(m) + \kappa_s c_s(t,x) - a_m m(t,x) R(t,x) + (\kappa_m + d_m) c_m(t,x) - (1+\gamma) m(t,x), \tag{34c}$$

$$\frac{\partial c_m(t,x)}{\partial t} = \mathcal{L}_c(c_m) + a_m m(t,x) R(t,x) - (\kappa_m + d_m + 1 + \gamma) c_m, \tag{34d}$$

$$\frac{\partial R(t,x)}{\partial t} = \mathcal{L}_R(R) + \alpha_R(t,x) + \big[ a_m m(t,x) R(t,x) + (\kappa_m + d_m + \gamma) c_m(t,x) \big] - R(t,x). \tag{34e}$$

where the spatial variable has been normalized by $L$ (cell-length) and the time variable has been normalized by $1/\mu$ the time scale associated with dilution. The flux dynamics and boundary conditions are given by,

$$\mathcal{L}_m(m) = -\frac{d}{dx}\big[J_m(x,m)\big], \quad J_m(x,m) = -\chi_m v_m(x)^2 \frac{d}{dx}\big[v_m(x)^{-1} m\big], \quad J_m(x,m)\big|_{x=0,1} = 0, \tag{35a}$$

$$\mathcal{L}_c(c_m) = -\frac{d}{dx}\big[J_c(x,c_m)\big], \quad J_c(x,c_m) = -\chi_c v_c(x)^2 \frac{d}{dx}\big[v_c(x)^{-1} c_m\big], \quad J_c(x,c_m)\big|_{x=0,1} = 0, \tag{35b}$$

$$\mathcal{L}_R(R) = -\frac{d}{dx}\big[J_R(x,R)\big], \quad J_R(x,R) = -\chi_R v_R(x)^2 \frac{d}{dx}\big[v_R(x)^{-1} R\big], \quad J_R(x,R)\big|_{x=0,1} = 0, \tag{35c}$$

$$\mathcal{L}_s(S) = -\frac{d}{dx}\big[J_s(x,S)\big], \quad J_s(x,S) = -\chi_s v_s(x)^2 \frac{d}{dx}\big[v_s(x)^{-1} S\big], \quad J_s(x,S)\big|_{x=0,1} = 0. \tag{35d}$$

where $v_m(x)$, $v_c(x)$, $v_R(x)$, and $v_s(x)$ are the available volume profiles for the mRNA, mRNA-ribosome complex, ribosome, and RNAP, respectively and $\chi_m = D_m/(L^2\mu)$, $\chi_c = D_c/(L^2\mu)$, $\chi_R = D_R/(L^2\mu)$, and $\chi_s = D_s/(L^2\mu)$, are the dimensionless diffusion coefficients for the mRNA, mRNA-ribosome complex, ribosome, and RNAP, respectively. The space averaged protein concentration $\bar{P}(t)$ is given by

$$\frac{d\bar{P}(t)}{dt} = \kappa_m \bar{c}_m(t) - \bar{P}(t), \quad \text{with} \quad \bar{c}_m(t) = \int_0^1 c_m(t,x) dx.$$

**Values for dimensionless parameters**:

We set all production rates with respect to that of RNAP such that $\bar{\alpha}_S = \int_0^1 \alpha_S(x)dx = 1$. All time scales relative to $\mu = 0.5$ 1/hr, consistent with the experiments [10] The total number of RNAP ($N_{\text{RNAP}}$) ranges between 2,000 -10,000 we took it to be 5,000 [9]. The total number of ribosomes ($N_{\text{ribo}}$) was taken to be $10,000$ and since both RNAP and ribosomes and RNAP are stable, it implies $\bar{\alpha}_r = \int_0^1 \alpha_r(x)dx = \frac{N_{\text{ribo}}}{N_{\text{RNAP}}} = 2$. mRNA degradation is about 10 times faster than dilution [7], therefore, $\gamma = 10$.The rate of transcription (translation) is about 80 (40) times faster than dilution [7], thus we choose $\kappa_s = 80$ and $\kappa_m = 40$. We assumed that the DNA is on a high copy plasmid ($\approx 500$ copies) and thus $\bar{D}_T = \int_0^1 D_T(x)dx = \frac{N_{\text{DNA}}}{N_{\text{RNAP}}} = 0.1$.

The association and dissociation rate constants are varied as shown below to show that all of our results hold dispiite fast binding and unbinding but we maintain the ratio $d_s/a_s = d_m/a_m = 1$.

The length of the cell is about $3\mu$m and thus $L = 1.5\mu$m [8]. The diffusion coefficient of RNAP is taken to be $D_s = 0.22\mu m^2/s$ [11] and thus $\chi_s = 704$. The diffusion coefficient of free ribosomes is taken to be $D_r = 0.4\mu m^2/s$ [8] and thus $\chi_r = 1280$. In [9], the diffusion coefficient of polysomes is $0.05 \pm 0.02\mu m^2/s$, and thus we take the diffusion coefficient of a free mRNA to be the upper bound $0.07\mu m^2/s$ and thus $\chi_m = \chi_c = 224$.

For the following, the spatial profiles for the production rates are given proportional to their functional form since the constant that fully specifies them is such that the production rate per-cell satisfies the above values.

**Additional simulation details for Figure 5-A in the Main Text**: $D_T(x) \propto e^{-20x}$ when DNA near mid cell and $D_T(x) \propto e^{20(x-1)}$ when DNA at cell poles. The RNAP production was kept roughly spatially constant $\alpha_s(x) \propto e^{-.001x}$. The binding and unbinding coefficients for DNA-RNAP were $a_s = 1000$ and $d_s = 1000$. We set $a_m = d_m = 0$ such that mRNA did not bind to ribosomes and thus the free amount of mRNA is equivalent to the total mRNA.

**Additional simulation details for Figure 5-B in the Main Text**: $D_T(x) \propto e^{-.001x}$ is chosen to be roughly constant. The RNAP production was kept roughly spatially constant $\alpha_s(x) \propto e^{-.001x}$. The ribosome production was kept roughly spatially constant $\alpha_r(x) \propto e^{-.001x}$. The RNAP radius of gyration was taken to be $r_s/r^* = 0.001$ such that its excluded volume effects were negligible. The binding and unbinding coefficients for DNA-RNAP were $a_s = 1000$ and $d_s = 1000$. The binding and unbinding coefficients for ribosome-mRNA were $a_m = 10$ and $d_m = 10$.

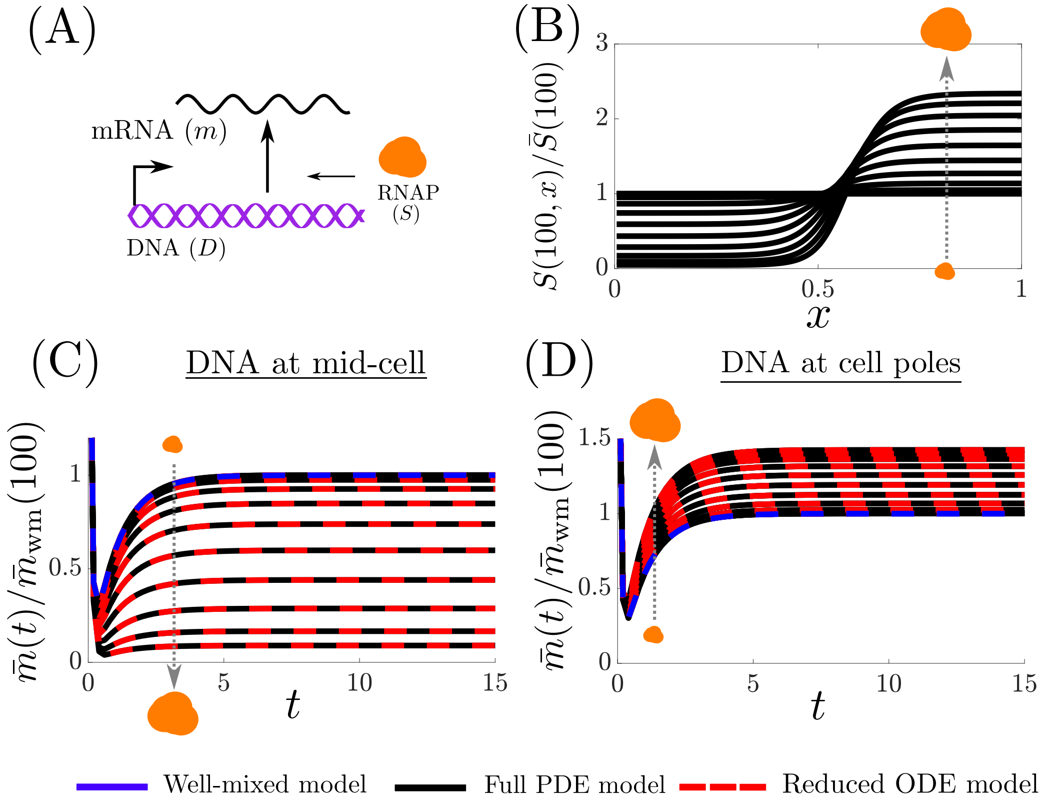


Figure B: **RNAP steady state spatial profiles and space averaged mRNA transients** For the following we refer to the well-mixed model as Equation 17 in the Main Text with $\theta_s = 1$ and $\theta_r = 1$. Here time is nondimensionalized with the time scale associated with dilution. (A) DNA transcribed by RNAP (S) to form mRNA (m) (B) The steady state RNAP spatial profile predicted by (34), normalized by spatial averaged value. From the results on the Main Text this should mirror the normalized available volume profile, which it does (Remark 1 in the Main Text). Note as the size of RNAP increases, it is further excluded from the chromosome. (C) The temporal space-average concentration of mRNA when the DNA is localized mid-cell for several sizes of RNAP for the well-mixed model, reduced ODE model (Equation 17 in the Main Text) , and PDE (34). (D) The temporal space-average concentration of mRNA when the DNA is localized near the cell poles for several sizes of RNAP for the well-mixed model, reduced ODE model(Equation 17 in the Main Text), and PDE (34). The simulation set up and parameters are identical to those of Figure 5-A in the Main Text.

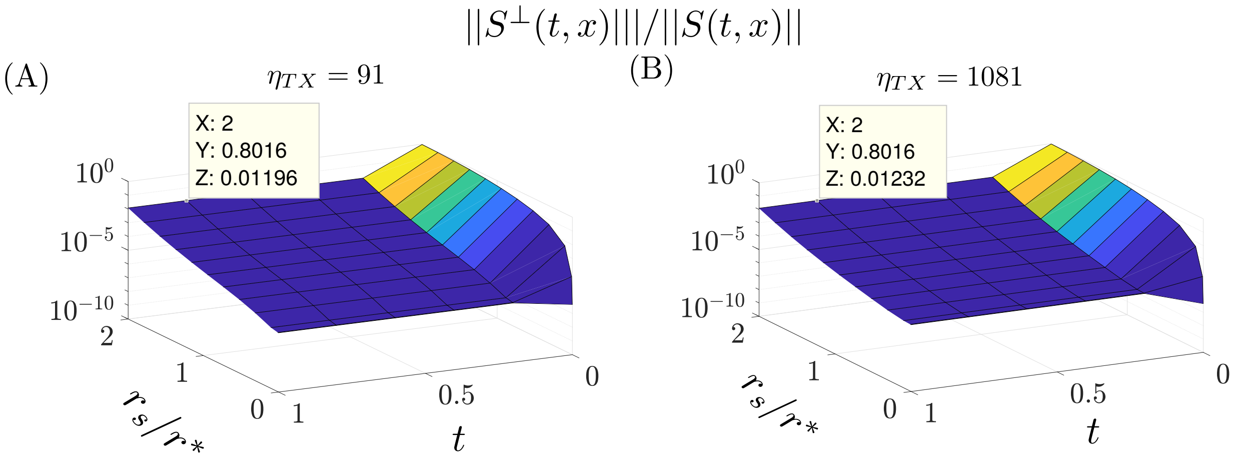


Figure C: **The error in the RNAP approximation for several binding and unbinding speeds between DNA and RNAP** Let $S(t,x)$ be as in (34) and $S^{\perp}(t,x) = S(t,x) - \bar{S}(t)\hat{v}_s(x)$ be the a measure of the error in our approximation, where $||y(t,x)|| = (\int_0^1 (y^2(t,x)))^{1/2}$, and $\eta_{\text{TX}} = 1/(\kappa_s + d_s + 1)$. The values of $\eta_{\text{TX}}$ are varied by modifying $d_s$ while maintaining $a_s/d_s = 1$. Here time is nondimensionalized with the time scale associated with dilution. (A) The relative error in time for several values of $r_s$ for $\eta_{\text{TX}} = 91 \implies d_s = 10$. (B) The relative error in time for several values of $r_s$ for $\eta_{\text{TX}} = 1081 \implies d_s = 1000$. For both values of $\eta_{\text{TX}}$ the error is high at $t = 0$ since the initial RNAP spatial profile is chosen to be a constant but quickly decays to less than 2%. The rest of the simulation set up and parameters are identical to those of Figure 5-A.

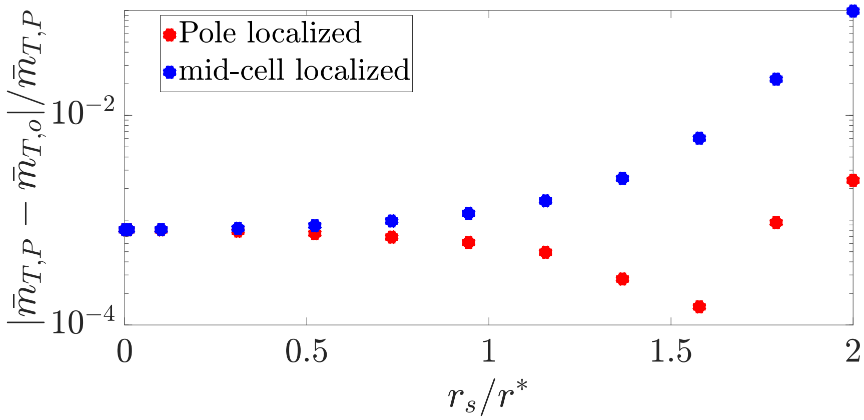


Figure D: **The relative error between the space averaged PDE model and the reduced ODE model from the data in Figure 5-A in the Main Text** The relative error in the steady state space averaged mRNA for the full PDE model ($\bar{m}_{T,P}$) and the reduced model ($\bar{m}_{T,o}$) from the data in Figure 5-A in the Main Text. When the DNA is pole localized the relative error is less than 1% and when the DNA is localized near mid-cell the error is less than 10%.

### 3.1 Multiple Ribosomes on a Single Strand of mRNA

The biochemical reactions that models a polysome with $N_r$ bound ribosomes are given by

$$\underbrace{\mathrm{m} + (\mathrm{N_r} - 1)\mathrm{R} \underset{\mathrm{d_l}}{\overset{\mathrm{a_l}}{\rightleftharpoons}} \mathrm{c_l},}_{\text{ribosome loading}} \quad \underbrace{\mathrm{c_l} + \mathrm{R} \underset{\mathrm{d_t}}{\overset{\mathrm{a_t}}{\rightleftharpoons}} \mathrm{c_t} \xrightarrow{\kappa_t} \mathrm{P} + \mathrm{R} + \mathrm{c_l},}_{\text{translation}} \tag{36}$$

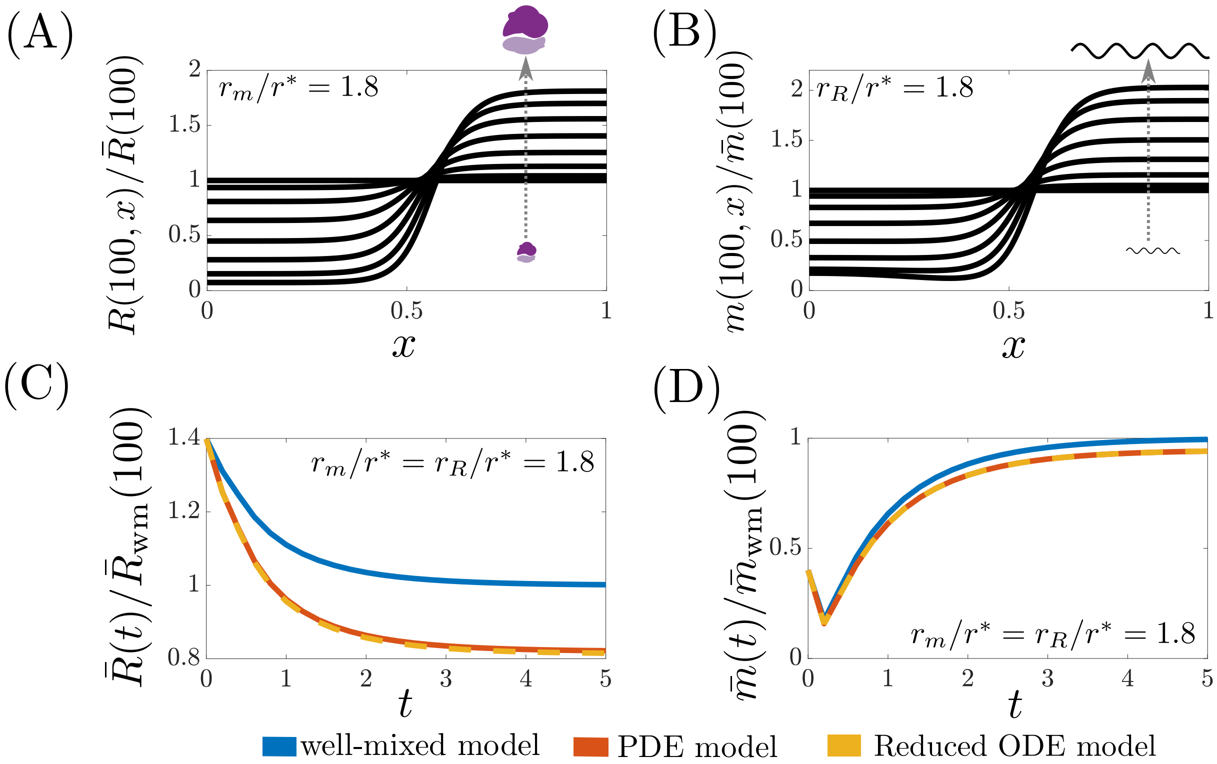


Figure E: **Ribosome and mRNA steady state spatial profiles and space averaged transients** For the following we refer to the well-mixed model as Equation 17 in the Main Text with $\theta_s = 1$ and $\theta_r = 1$. Here time is nondimensionalized with the time scale associated with dilution. (A) The steady state ribosome spatial profile predicted by (34), normalized by spatial averaged value. From the results on the Main Text this should mirror the normalized available volume profile, which it does. Note as the size of the ribosome increases, it is further excluded from the chromosome. (B) The steady state mRNA spatial profile predicted by (34), normalized by spatial averaged value. From the results on the Main Text this should mirror the normalized available volume profile, which it does. Note as the size of the mRNA increases, it is further excluded from the chromosome. (C) The temporal space averaged concentration of ribosomes normalized by the steady state of the well-mixed model for the reduced ODE model (Equation 17 in the Main Text) and the PDE model (34) when $r_m/r^* = r_R/r^* = 1.8$. (D)The temporal space averaged concentration of mRNA normalized by the steady state of the well-mixed model for the reduced ODE model (Equation 17 in the Main Text) and the PDE model (34) when $r_m/r^* = r_R/r^* = 1.8$. The simulation set up and parameters are identical to those of Figure 5-B in the Main Text.

where the first and second reaction model the loading and translation steps, respectively. The translation dynamics corresponding to (36) are given by

$$\frac{\partial m(t,x)}{\partial t} = \mathcal{L}_m(m) + \kappa_s c_s(t,x) - a_l m(t,x) R^{N_r-1}(t,x) + d_l c_l(t,x) - (1+\gamma)m(t,x), \tag{37a}$$

$$\frac{\partial c_l(t,x)}{\partial t} = \mathcal{L}_{c,l}(c_l) + a_l m(t,x) R^{N_r-1}(t,x) - d_l c_l(t,x) - a_t c_l(t,x) R(t,x) + (d_t+\kappa_t)c_t(t,x) - (\gamma+1)c_l(t,x), \tag{37b}$$

$$\frac{\partial R(t,x)}{\partial t} = \mathcal{L}_R(R) + \alpha_R(t,x) + (N_r-1)\left[a_l m(t,x) R^{N_r-1}(t,x) - d_l c_l(t,x)\right] \tag{37c}$$

$$- a_t c_l(t,x) R(t,x) + (d_t+\kappa_t)c_t(t,x) - R(t,x), \tag{37d}$$

$$\frac{\partial c_t(t,x)}{\partial t} = \mathcal{L}_{c,t}(c_t) + a_t c_l(t,x) R - (d_t+\kappa_t)c_t(t,x) - (\gamma+1)c_t(t,x), \tag{37e}$$

$$\tag{37f}$$

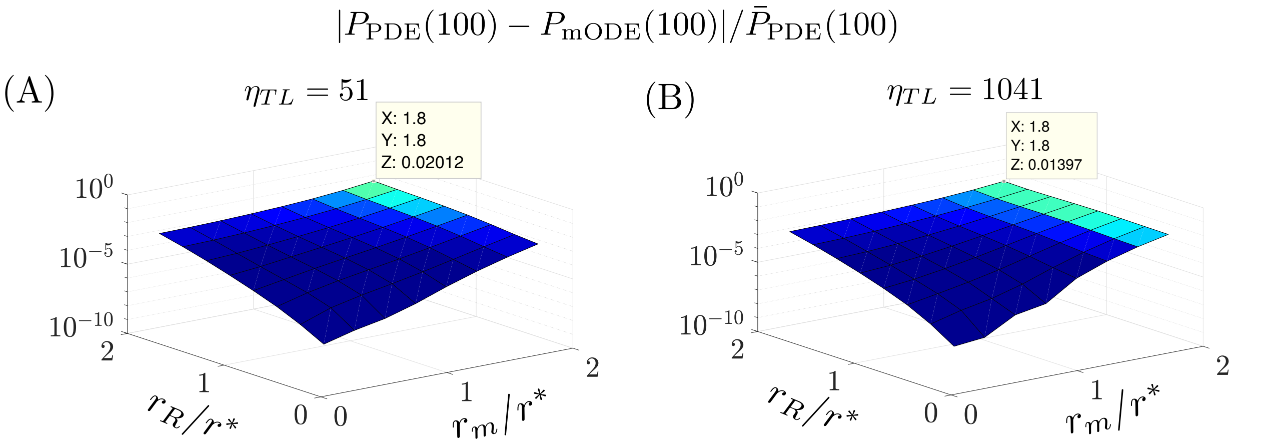


Figure F: **PDE and reduced ODE model agree well in protein production example** Let $P_{\mathrm{PDE}}(100)$ be the steady state protein space averaged concentration predicted by (34) and $P_{\mathrm{ODE}}(100)$ be the steady state protein space averaged concentration predicted by Equation 17 in the Main Text. Let $\eta_{\mathrm{TL}} = 1/(\kappa_m + d_m + 1 + \gamma_m)$. The values of $\eta_{\mathrm{TL}}$ are varied by modifying $d_m$ while maintaining $a_m/d_m = 1$. (A) The relative error for several values of $r_m$ and $r_s$ for $\eta_{\mathrm{TL}} = 51 \implies d_m = 10$. (B) The relative error for several values of $r_m$ and $r_s$ for $\eta_{\mathrm{TX}} = 1041 \implies d_m = 1000$. For both cases the relative error is less than 2.1% The rest of the simulation set up and parameters are identical to those of Figure 5-B.

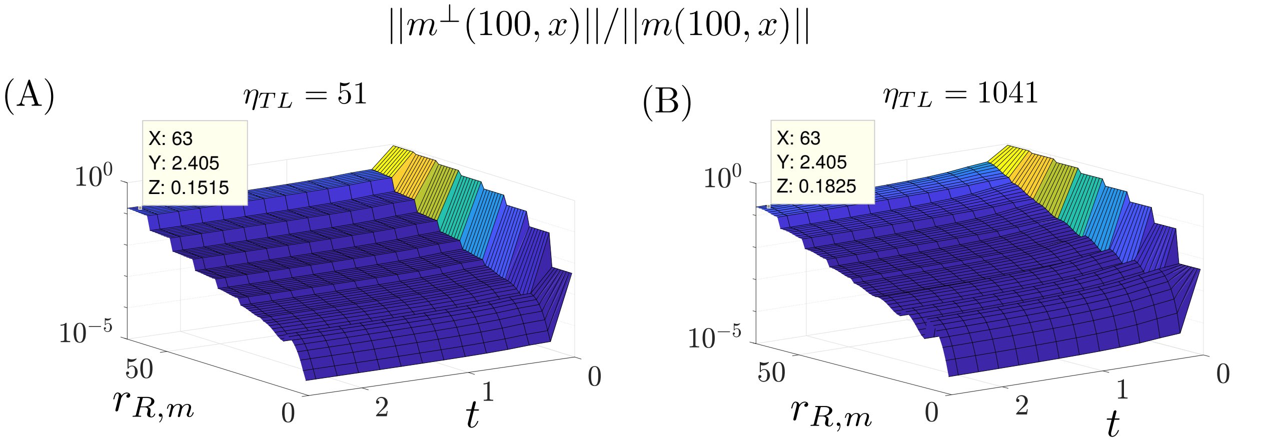


Figure G: **The error in the mRNA approximation for several binding and unbinding speeds between mRNA and ribosome** Let $m(t,x)$ be as in (34) and $m^{\perp}(t,x) = m(t,x) - \bar{m}(t)\hat{v}_m(x)$ be the a measure of the error in our approximation, where $||y(t,x)|| = (\int_0^1 (y^2(t,x)))^{1/2}$, and $\eta_{\mathrm{TL}} = 1/(\kappa_m + d_m + 1 + \gamma_m)$. The values of $\eta_{\mathrm{TL}}$ are varied by modifying $d_m$ while maintaining $a_m/d_m = 1$. Here $r_R, m$ is a sequence corresponding to the mRNA and ribosome pairs from Figure 5-B in the Main Text. Here time nondimensionalized with the time scale associated with dilution. (A) The relative error in time for several values of $r_R, m$ for $\eta_{\mathrm{TL}} = 51 \implies d_s = 10$. (B) The relative error in time for several values of $r_R, m$ for $\eta_{\mathrm{TL}} = 1041 \implies d_s = 1000$. For both values of $\eta_{\mathrm{TL}}$ the error is high at $t = 0$ since the initial mRNA spatial profile is chosen to be a constant but quickly decays to less than 20%. The rest of the simulation set up and parameters are identical to those of Figure 5-B.

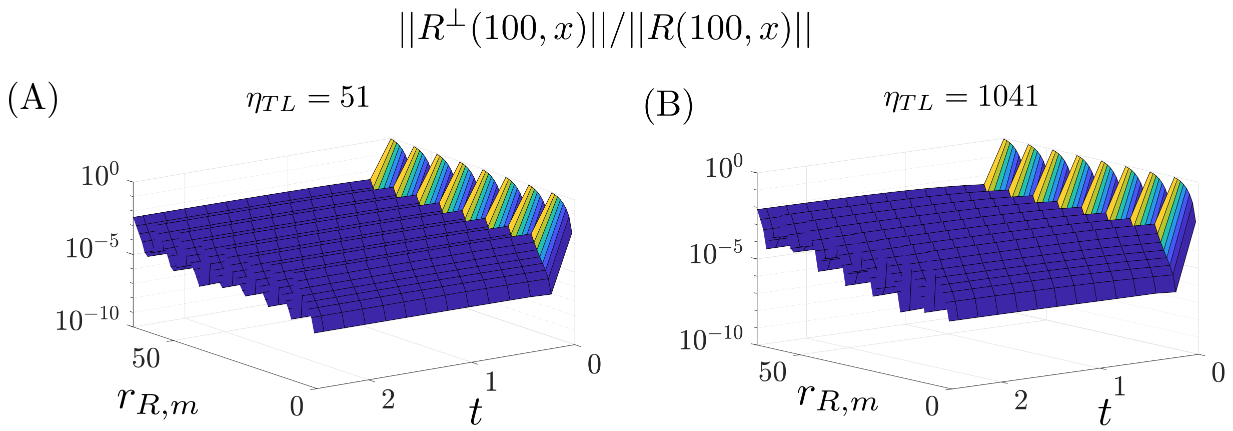


Figure H: **The error in the ribosome approximation for several binding and unbinding speeds between mRNA and ribosome** Let $m(t,x)$ be as in (34) and $R^{\perp}(t,x)=R(t,x)-\bar{R}(t)\hat{v}_r(x)$ be the a measure of the error in our approximation, where $||y(t,x)||=(\int_0^1(y^2(t,x)))^{1/2}$, and $\eta_{\text{TL}}=1/(\kappa_m+d_m+1+\gamma_m)$. The values of $\eta_{\text{TL}}$ are varied by modifying $d_m$ while maintaining $a_m/d_m=1$. Here $r_R,m$ is a sequence corresponding to the mRNA and ribosome pairs from Figure 5-B. Here time nondimensionalized with the time scale associated with dilution. (A) The relative error in time for several values of $r_R,m$ for $\eta_{\text{TL}}=51 \implies d_s=10$. (B) The relative error in time for several values of $r_R,m$ for $\eta_{\text{TL}}=1041 \implies d_s=1000$. For both values of $\eta_{\text{TL}}$ the error is high at $t=0$ since the initial mRNA spatial profile is chosen to be a constant but quickly decays to less than 1%. The rest of the simulation set up and parameters are identical to those of Figure 5-B.

where $c_s(t,x)$ is given by (34), $\mathcal{L}_m(m)$ and $\mathcal{L}_R(R)$ are given by (35) and

$$\mathcal{L}_{c,l}(c_l)=-\frac{d}{dx}\big[J_{c,l}(x,c_l)\big],\quad J_{c,l}(x,c_l)=-\chi_c v_l(x)^2\frac{d}{dx}\big[v_l(x)^{-1}c_l\big],\quad J_{c,l}(x,c_l)\big|_{x=0,1}=0,$$

$$\mathcal{L}_{c,t}(c_t)=-\frac{d}{dx}\big[J_{c,t}(x,c_t)\big],\quad J_{c,t}(x,c_t)=-\chi_c v_t(x)^2\frac{d}{dx}\big[v_t(x)^{-1}c_t\big],\quad J_{c,t}(x,c_t)\big|_{x=0,1}=0,$$

where $v_l(x)=v_m(x)v_R^{N_r-1}(x)$ and $v_t(x)=v_l(x)v_R(x)$. Integrating (37) in space yields:

$$\frac{d\bar{m}(t)}{dt}=\kappa_s\bar{c}_s(t)-a_l\theta_l(t)\bar{m}(t)\bar{R}^{N_r-1}(t)+d_l\bar{c}_l(t)-(1+\gamma)\bar{m}(t), \tag{38a}$$

$$\frac{d\bar{c}_l(t)}{dt}=a_l\theta_l(t)\bar{m}(t)\bar{R}^{N_r-1}(t)-d_l\bar{c}_l(t)-a_t\theta_t(t)\bar{c}_l(t)\bar{R}(t)+(d_t+\kappa_t)\bar{c}_t(t)-(\gamma+1)\bar{c}_l(t), \tag{38b}$$

$$\frac{d\bar{R}(t)}{dt}=\bar{\alpha}_R(t)+(N_r-1)\big[a_l\theta_l(t)\bar{m}(x)\bar{R}^{N_r-1}(t)-d_l\bar{c}_l(t)\big] \tag{38c}$$

$$-a_t\theta_t(t)\bar{c}_l(t)\bar{R}(t,x)+(d_t+\kappa_t)\bar{c}_t(t)-\bar{R}(t), \tag{38d}$$

$$\frac{d\bar{c}_t(t)}{dt}=a_t\theta_t(t)\bar{c}_l(t)\bar{R}(t)-(d_t+\kappa_t)\bar{c}_t(t)-(\gamma+1)\bar{c}_t(t), \tag{38e}$$

$$\frac{d\bar{P}(t)}{dt}=\kappa_t\bar{c}_t(t)-\bar{P}(t) \tag{38f}$$

where

$$\theta_l(t)=\frac{\int_0^1 m(t,x)R^{N_r-1}(t,x)dx}{[\int_0^1 m(t,x)dx][\int_0^1 R(t,x)dx]^{N_r-1}},\quad \theta_t(t)=\frac{\int_0^1 c_l(t,x)R(t,x)dx}{[\int_0^1 c_l(t,x)dx][\int_0^1 R(t,x)dx]}. \tag{39}$$

The production rate of $\bar{P}$ denoted by $\bar{\alpha}_P$ is given by $\bar{\alpha}_P=\kappa_t\bar{c}_t(t)$. From our analysis in Section 1.2, we expect that $R(t,x)\approx\bar{R}(t)\hat{v}_r(x)$, $m(t,x)\approx\bar{m}(t)\hat{v}_m(x)$, and $c_l(t,x)\approx\bar{c}_l(t)v_l(x)$ (this is verified computationally in Figure I), and thus we can estimate $\theta_l^*(t)$ and $\theta_t^*(t)$ by the constants

$$\theta_l(t)\approx\theta_l^*=\frac{\int_0^1 v_m(x)v_R^{N_r-1}(x)dx}{[\int_0^1 v_m(x)dx][\int_0^1 v_R(x)dx]^{N_r-1}},\quad \theta_t(t)\approx\theta_t^*=\frac{\int_0^1 v_l(x)v_R(x)dx}{[\int_0^1 v_l(x)dx][\int_0^1 v_R(x)dx]}, \tag{40}$$

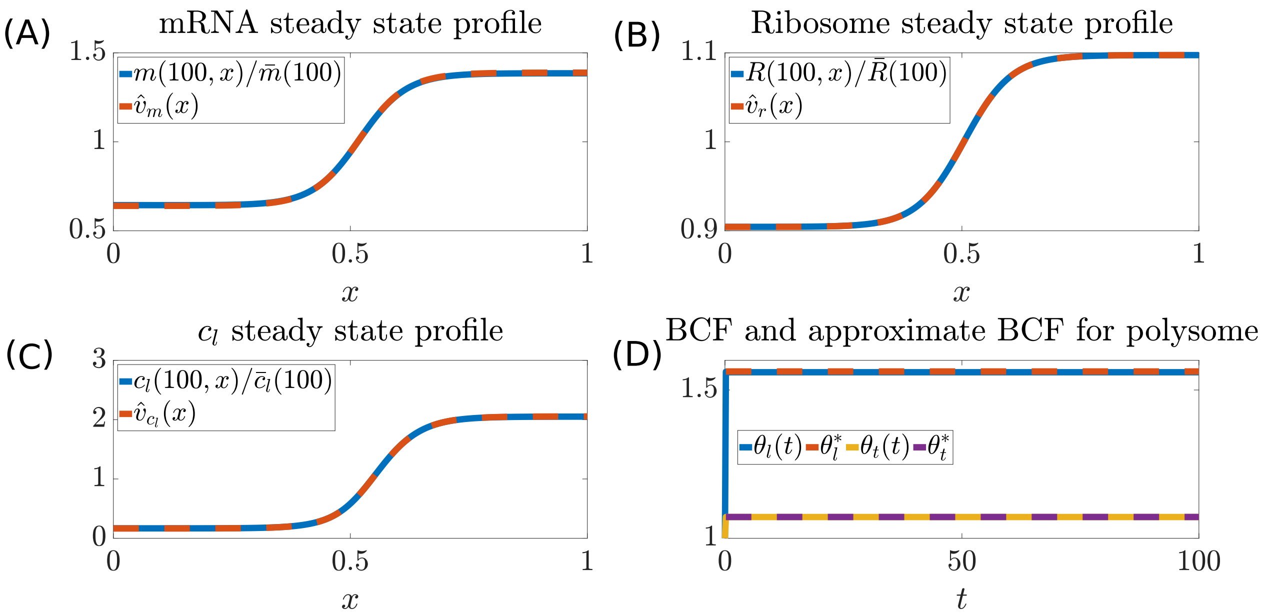


Figure I: **BCF for Polysome with 10 Ribosomes** (A) The steady state mRNA spatial concentration profile ($m(100,x)$) predicted by (37) normalized by the space averaged concentration along with the normalized mRNA available volume profile ($\hat{v}_m(x) = v_m(x)/\int_0^1 v_m(x)dx$). (B) The steady state ribosome spatial concentration profile ($R(100,x)$) predicted by (37) normalized by the space averaged concentration along with the normalized mRNA available volume profile ($\hat{v}_r(x) = v_r(x)/\int_0^1 v_r(x)dx$). (C) The steady state polysome (loaded with 9 ribosomes) spatial concentration profile ($c_l(100,x)$) predicted by (37) normalized by the space averaged concentration along with the normalized mRNA available volume profile ($\hat{v}_{c_l}(x) = v_{c_l}(x)/\int_0^1 v_{c_l}(x)dx$). (D) The BCF's $\theta_l(t)$ and $\theta_t(t)$ given by (39) and their constant approximation $\theta_l^*$ and $\theta_t^*$ given by (40). For these simulations, we do not model transcription directly but instead set $\kappa_s c_s(t,x) = 1$, such that at steady state $m_T(x) = m(x)+c_l(x)+c_t(x) = \kappa_s c_s(t,x)/(\gamma+1) = 0.09$ for $\gamma = 10$. The used parameter values are $N_r = 10$, $\chi_m = \chi_c = 224$ , $\chi_r = 1280$, $a_t = 10$, $d_t = 10$, $\kappa_t = 40$, $a_l = a_t^{N_r-1}$ $d_l = d_t^{N_r-1}$, $\alpha_r(t,x) = 1$, $r_m/r^* = 0.88$ and $r_R/r^* = 0.44$.

this is verified via simulation in Figure I)-D.

Let $K_d = (d_l/a_l)^{1/(N_r-1)}$, $K_t = (d_t+\kappa_t)/a_t$, $\beta_l = (\gamma+1)/d_l$, and $\beta_t = (\gamma+1)/(\kappa_t+d_t)$, if $\beta_l, \beta_t, \beta_t R/K_t \ll 1$ (dilution and mRNA degradation is much slower the rate of ribosome unbinding and $K_t$ is sufficiently large), then a simple expression for the steady state protein is given by

$$\bar{P} = \kappa_t \bar{c}_t = \kappa_t \bar{m}_T \underbrace{\theta_t^* \bar{R}/K_t}_{\text{translation}} \underbrace{\frac{\theta_l^* (\bar{R}/K_d)^{N_r-1}}{1+\theta_l^* (\bar{R}/K_d)^{N_r-1}}}_{\text{ribosome loading}},$$

where $\bar{m}_T = \bar{m} + \bar{c}_l + \bar{c}_t = \kappa_s \bar{c}_s/(\gamma+1)$ is the total mRNA.

# 4 Transcription Factor Regulation

Intracellular signaling to control gene expression is often done via transcription factors (TFs). In this section we model a general transcription factor architecture where the repressor $\mathrm{P_r}$ dimerizes to form $\mathrm{c_1}$ (e.g., TetR dimerizes before targeting gene [12]) and then blocks the transcription of gene $D$ that produces protein P. The biochemical reactions corresponding to this process are:

$$\emptyset \xrightarrow{\alpha} \mathrm{P_r}, \quad \underbrace{\mathrm{P_r} + \mathrm{P_r} \underset{\mathrm{d_1}}{\overset{\mathrm{a_1}}{\rightleftharpoons}} \mathrm{c_1}}_{\text{Case I}}, \quad \underbrace{\mathrm{c_1} + \mathrm{D} \underset{\mathrm{d_2}}{\overset{\mathrm{a_2}}{\rightleftharpoons}} \mathrm{c_2}}_{\text{Case II}}, \quad \mathrm{D} \xrightarrow{\kappa} \mathrm{P}, \tag{41}$$

where $\alpha$ is the production rate of $\mathrm{P_r}$, $a_1$ ($d_1$) is the association (dissociation) constant to form the $\mathrm{c_1}$ complex, $a_2$ ($d_2$) is the association (dissociation) constant to form the the $\mathrm{c_2}$ complex, and $\kappa$ is the catalytic rate to

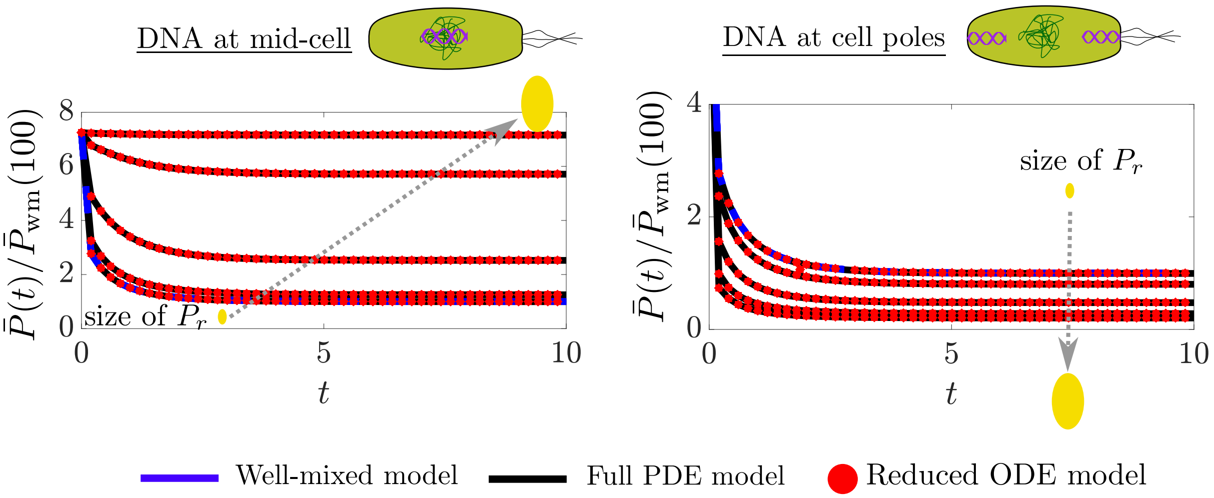


Figure J: **Transcription factor regulation transient response** The transient response corresponding to Figure 6 in the Main Text when $\bar{P}_r/K = 2.5$

produce protein P. Since the repressor $\mathrm{P_r}$, freely diffuses, the dimerization reaction belongs to Case I. The gene D is spatially fixed and it is repressed by the freely diffusing $\mathrm{c_1}$, thus this interaction falls under Case II. We assume that the total concentration of $D$ is conserved, so that $D_T(x) = D(t,x) + c_2(t,x)$. The reaction diffusion equations corresponding to (41) are

$$\frac{\partial P_r(t,x)}{\partial t} = \chi_r \frac{d}{dx}\left[v_{P_r}^2(x)\frac{d}{dx}\left[\frac{P_r(t,x)}{v_{P_r}(x)}\right]\right] + \alpha(t,x) - \gamma_r P_r(t,x), \tag{42a}$$

$$\frac{\partial c_1(t,x)}{\partial t} = \chi_c \frac{d}{dx}\left[v_{c_1}^2(x)\frac{d}{dx}\left[\frac{c_1(t,x)}{v_{c_1}(x)}\right]\right] + a_1 P_r^2(t,x) - d_1 c_1(t,x) - a_2 c_1(t,x) D(t,x) + d_2 c_2(t,x), \tag{42b}$$

$$\frac{\partial c_2(t,x)}{\partial t} = a_2 c_1(t,x) D(t,x) - d_2 c_2(t,x), \tag{42c}$$

$$D(t,x) = D_T(x) - c_2(t,x), \tag{42d}$$

where $v_{P_r}(x)$ ($\chi_r = D_r/(L^2\mu)$) and $v_{c_1}(x)$ ($\chi_c = D_{c_1}/(L^2\mu)$) are the available volume profiles (dimensionless diffusion coefficients) of $\mathrm{P_r}$ and $\mathrm{c_1}$, respectively, and from Equation 10 in the Main Text, $v_{c_1}(x) = v_{P_r}^2(x)$. The boundary conditions corresponding to (42) are

$$\left[v_{P_r}^2(x)\frac{d}{dx}\left[\frac{P_r(t,x)}{v_{P_r}(x)}\right]\right]_{x=0,1} = 0, \quad \left[v_{c_1}^2(x)\frac{d}{dx}\left[\frac{c_1(t,x)}{v_{c_1}(x)}\right]\right]_{x=0,1} = 0.$$

**Values for dimensionless parameters**: The growth rate we used to nondimensionalize the time scales was $\mu = 0.5$ 1/hr, consistent with the experiments [10]. The length of the cell is about $3\mu$m and thus $L = 1.5\mu$m [8]. The diffusion coefficient of the transcription factor is taken to be $D_r = D_{c_1} = 0.4\mu m^2/s$ (that of LacI) [13] and thus $\chi_r = \chi_c = 1280$. The transcription factor was assumed to be stale thus $\gamma_r = \mu$. The total concentration of D given by $\bar{D}_T$ was used to nondimensionalize the other concentration variables such that $\bar{D}_T = 1$.

**Additional simulation details for Figure 6 in the Main Text**: $D_T(x) \propto e^{-20x}$ when DNA near mid cell and $D_T(x) \propto e^{20(x-1)}$ when DNA at cell poles. The transcription factor production was kept roughly spatially constant $\alpha_s(x) \propto e^{-.001x}$. The binding and unbinding coefficients were chosen to be $a_1 = a_2 = 1000$ and $d_1 = d_2 = 1000$ such that the dissociations constants $K_{d,1} = \frac{d_1}{a_1} = K_{d,2} = \frac{d_2}{a_2} = 1$.

**Approxmating BCF from known parameter values:**

From Equations 2 and 10 in the Main Text, we observe that the effective radius of gyration of a dimer complex is $\sqrt{2}r$, where $r$ is the radius of gyration of the individual species. In [14] it was estimated that the radius of gyration for the Tet repressor dimer is 3.1 nm and thus we estimate the radius of gyration of the monomer as $r = 3.1/\sqrt{2}$. From the expression for $\theta^*$ given by Equation 26 in the Main Text, Figure 4-B in the Main Text, and $r = 3.1/\sqrt{2}$, we have that $\theta^* \approx 0.99$ and $\theta^* \approx 1.01$, when the target DNA is near mid-cell

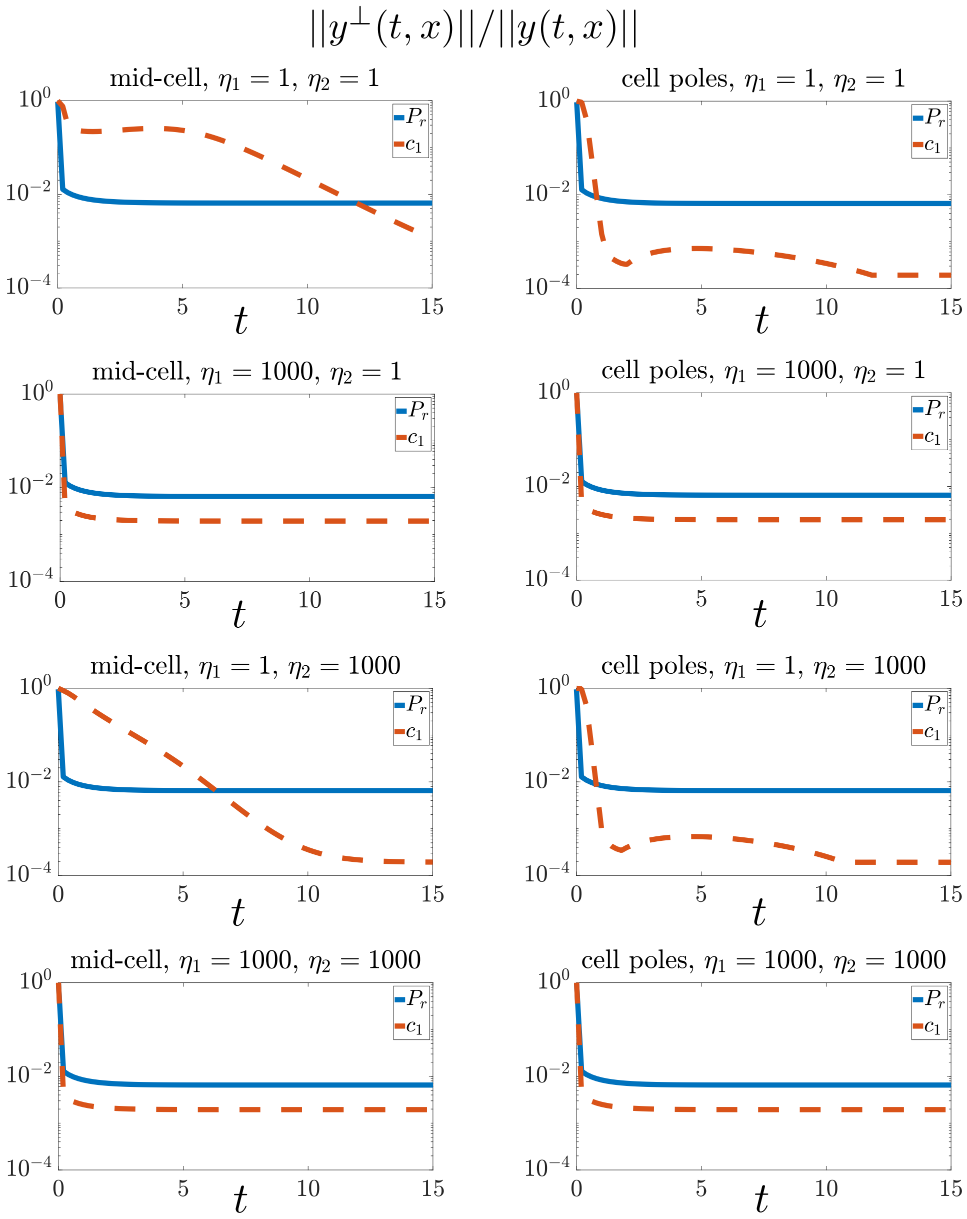


Figure K: **Infinite dimensional dynamics decay in time independently of binding/unbinding speed** Let $y(t,x)$ be as in (42) (represents $P_r(t,x)$ or $c_1(t,x)$) and let $y^{\perp}(t,x) = y(t,x) - \bar{y}(t)v(x)$ be the a measure of the error in our approximation, where $v(x)$ is the available volume profile of the species. Let $||y(t,x)|| = (\int_0^1 (y^2(t,x)))^{1/2}$, $\eta_1 = d_1/\mu$, and $\eta_2 = d_2/\mu$ and recall that in the simulations we keep $d_1/a_1 = d_2/a_2 = 1$. We show $||y^{\perp}||/||y||$, the relative error for several values of $\eta_1$ and $\eta_2$ over dimensionless time (with respect to $\mu$) both when the DNA is near mid-cell and the cell poles. The other simulation parameters are identical to those used to generate Figure 6 in the Main Text. For each time point shown, we took the max relative error with respect to the sizes of $\mathrm{P_r}$ used to generate Figure 6 in the Main Text. The error is high at $t = 0$ since the initial spatial profiles were chosen to be a constant but note that they quickly decay.

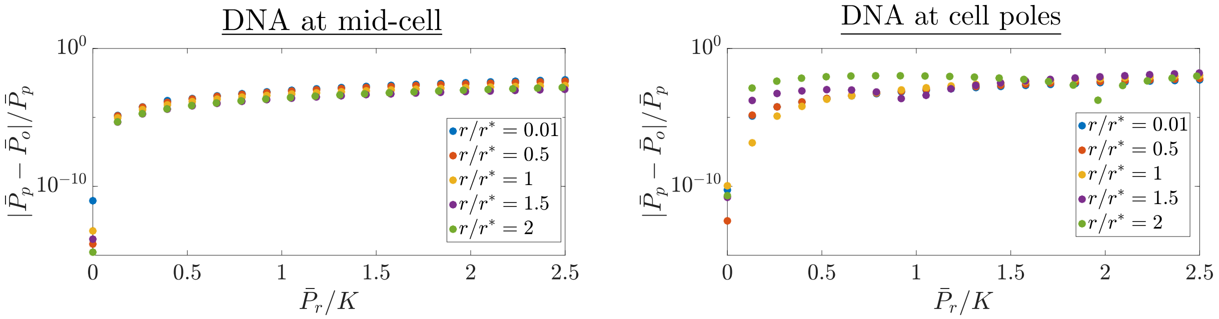


Figure L: **The relative error between the space averaged PDE model and the reduced ODE model from the data in Figure 6 in the Main Text** The relative error in the steady state space averaged protein for the full PDE model ($\bar{P}_P$) and the reduced model ($\bar{P}_o$) from the data in Figure 6 in the Main Text. The errors are less than 1.7% both when the DNA is localized at mid-cell and at the cell poles.

and the cell poles, respectively. Thus, for TetR, the binding strength between the repressor and the DNA varies by about 1% with respect to a well-mixed model in this parameter range. In [15] it was estimated that the radius of gyration for the Lac repressor tetramer is $r = 5.3$ nm. Assuming that the tetramer is made up of two dimers, then the radius of gyration of each individual dimer is given by $r = 5.3/\sqrt{2}$. From the expression for $\theta^*$ given by Equation 26 in the Main Text, Figure 4-B in the Main Text, and $r = 5.3/\sqrt{2}$, we have that $\theta^* \approx 0.97$ and $\theta^* \approx 1.03$, when the target DNA is near mid-cell and the cell poles, respectively. Thus, for the Lac repressor the binding strength between the transcription factor and the DNA varies by about 3% with respect to a well-mixed model in this parameter range.

While we could not find an exact value for the radius of gyration of the dcas9-gRNA complex, in [16] it was shown that the size of the Cas9-gRNA complex is roughly 10 nm. If we assume this value to be the radius of gyration, from Figure 4-B in the Main Text, we have that the BCF for this complex is $\theta^* = 0.9$ and $\theta^* = 1.1$ when the target DNA is near mid-cell and the cell poles, respectively. Thus, the binding strength between the Cas9-gRNA complex and the DNA varies s by about 10% with respect to a well-mixed model in this parameter range.

# 5 Oscillator

Now we consider the repressor activator clock genetic circuit [17]. This circuit produces sustained oscillations if tuned within an appropriate parameter range [18, 19]. The circuit consists of two proteins $\mathrm{P_a}$ and $\mathrm{P_r}$. Protein $\mathrm{P_a}$, is an activator which dimerizes to form $\mathrm{P_{a,2}}$ and then binds to its own gene $\mathrm{D_a}$ to form complex $\mathrm{c_{a,1}}$ to initiate transcription. The dimer $\mathrm{P_{a,2}}$ also finds to the gene $\mathrm{D_r}$, which transcribes $\mathrm{P_r}$ to form complex $\mathrm{c_{a,2}}$ and initiates transcription. Protein $\mathrm{P_r}$, dimerizes to form $\mathrm{P_{r,2}}$ and then represses $\mathrm{P_a}$ by binding to $\mathrm{D_a}$ to form complex $\mathrm{c_r}$. The biochemical equations corresponding to this circuit are:

$$\underbrace{\mathrm{P_a} + \mathrm{P_a} \underset{\mathrm{d_1}}{\overset{\mathrm{a_1}}{\rightleftharpoons}} \mathrm{P_{a,2}},}_{\text{Case I, P}_a\text{ diffuses}} \quad \underbrace{\mathrm{P_r} + \mathrm{P_r} \underset{\mathrm{d_2}}{\overset{\mathrm{a_2}}{\rightleftharpoons}} \mathrm{P_{r,2}},}_{\text{Case II, P}_r\text{ diffuses}} \quad \underbrace{\mathrm{P_{a,2}} + \mathrm{D_a} \underset{\mathrm{d_3}}{\overset{\mathrm{a_3}}{\rightleftharpoons}} \mathrm{c_{a,1}}, \quad \mathrm{P_{r,2}} + \mathrm{D_a} \underset{\mathrm{d_5}}{\overset{\mathrm{a_5}}{\rightleftharpoons}} \mathrm{c_r},}_{\text{Case III, P}_{a,2}\text{ and P}_{r,2}\text{ diffuse and D}_\mathrm{a}\text{ fixed}} \quad \underbrace{\mathrm{P_{a,2}} + \mathrm{D_r} \underset{\mathrm{d_4}}{\overset{\mathrm{a_4}}{\rightleftharpoons}} \mathrm{c_{a,2}},}_{\text{Case II, P}_{a,2}\text{ diffuse and D}_\mathrm{r}\text{ fixed}} \tag{43a}$$

$$\mathrm{D_a} \xrightarrow{\kappa_1} \mathrm{P_a}, \quad \mathrm{D_r} \xrightarrow{\kappa_2} \mathrm{P_r}, \quad \mathrm{P_a} \xrightarrow{\gamma_\mathrm{a}} \emptyset, \quad \mathrm{P_r} \xrightarrow{\gamma_\mathrm{r}} \emptyset, \quad \mathrm{c_{a,1}} \xrightarrow{\kappa_3} \mathrm{P_a}, \quad \mathrm{c_{a,2}} \xrightarrow{\kappa_4} \mathrm{P_r}, \tag{43b}$$

where $a_i$ ($d_i$) for $i = 1, \dots, 5$ are association (dissociation) rate constants, $\gamma_a$ ($\gamma_r$) is the degradation rate of $\mathrm{P_a}$ ($\mathrm{P_r}$ ) $\kappa_1$ ($\kappa_2$) is the basal rate at which gene $\mathrm{D_a}$ ($\mathrm{D_r}$) is transcribed, and $\kappa_3$ ($\kappa_4$) is the rate at which the DNA-transcription-factor complexes are transcribed for $\mathrm{D_a}$ ($\mathrm{D_r}$). We assume that the total concentration of $\mathrm{D_a}$ is conserved, so that $D_{a,T}(x) = D_a(t,x) + c_{a,1}(t,x) + c_r(t,x)$. Similarly, we assume that the total concentration of $\mathrm{D_r}$ is conserved, so that $D_{r,T}(x) = D_r(t,x) + c_{a,2}(t,x)$. The spatiotemporal dynamics describing (43) are given by

$$
\begin{aligned}
\frac{\partial P_a(t,x)}{\partial t} &= \chi_a \frac{d}{dx}\left[v_{P_a}^2(x)\frac{d}{dx}\left[\frac{P_a(t,x)}{v_{P_a}(x)}\right]\right] + \kappa_1 D_a(t,x) + \kappa_3 c_{a,1}(t,x) - \gamma_a P_a(t,x),\\
\frac{\partial P_r(t,x)}{\partial t} &= \chi_r \frac{d}{dx}\left[v_{P_r}^2(x)\frac{d}{dx}\left[\frac{P_r(t,x)}{v_{P_r}(x)}\right]\right] + \kappa_2 D_r(t,x) + \kappa_4 c_{a,2}(t,x) - \gamma_r P_r(t,x),\\
\frac{\partial P_{a,2}(t,x)}{\partial t} &= \chi_a \frac{d}{dx}\left[v_{P_{a,2}}^2(x)\frac{d}{dx}\left[\frac{P_{a,2}(t,x)}{v_{P_{a,2}}(x)}\right]\right] + a_1 P_a^2(t,x) - d_1 P_{a,2}(t,x)\\
&\quad - a_3 P_{a,2}(t,x)D_a(t,x) + d_3 c_{a,1}(t,x) - a_4 P_{a,2}(t,x) D_r(t,x) + d_4 c_{a,2}(t,x),\\
\frac{\partial P_{r,2}(t,x)}{\partial t} &= \chi_r \frac{d}{dx}\left[v_{P_{r,2}}^2(x)\frac{d}{dx}\left[\frac{P_{r,2}(t,x)}{v_{P_{r,2}}(x)}\right]\right] + a_2 P_r^2(t,x) - d_2 P_{r,2}(t,x)\\
&\quad - a_5 P_{r,2}(t,x) D_a(t,x) + d_5 c_r(t,x),\\
\frac{\partial c_{a,1}(t,x)}{\partial t} &= a_3 P_{a,2}(t,x)D_a(t,x) - d_3 c_{a,1}(t,x), \quad \frac{\partial c_{a,2}(t,x)}{\partial t} = a_4 P_{a,2}(t,x) D_r(t,x) - d_4 c_{a,2}(t,x),\\
\frac{\partial c_r(t,x)}{\partial t} &= a_5 P_{r,2}(t,x) D_a(t,x) - d_5 c_r(t,x),\\
D_a(t,x) &= D_{a,T}(x) - c_{a,1}(t,x) - c_r(t,x), \quad D_r(t,x) = D_{r,T}(x) - c_{a,2}(t,x)
\end{aligned}
\tag{44}
$$

where $v_{P_a}(x)$, $v_{P_r}(x)$, $v_{P_{a,2}}(x)$, and $v_{P_{r,2}}(x)$ are the available volume profiles of $\mathrm{P_a}$, $\mathrm{P_r}$, $\mathrm{P_{a,2}}$, and $\mathrm{P_{r,2}}$, respectively, $\chi_a = D_a/(L^2\mu)$ is the dimensionless diffusion coefficient of $\mathrm{P_a}$ and $\mathrm{P_{a,2}}$, $\chi_r = D_r/(L^2\mu)$ is the dimensionless diffusion coefficient of $\mathrm{P_r}$ and $\mathrm{P_{r,2}}$. From Equation 10 in the Main Text, $v_{P_{a,2}}(x) = v_{P_a}^2(x)$ and $v_{P_{r,2}}(x) = v_{P_r}^2(x)$. The boundary conditions corresponding to (44) are

$$
\left[v_{P_a}^2(x)\frac{d}{dx}\left[\frac{P_a(t,x)}{v_{P_a}(x)}\right]\right]_{x=0,1} = 0, \quad \left[v_{P_r}^2(x)\frac{d}{dx}\left[\frac{P_r(t,x)}{v_{P_r}(x)}\right]\right]_{x=0,1} = 0,
$$

$$
\left[v_{P_{a,2}}^2(x)\frac{d}{dx}\left[\frac{P_{a,2}(t,x)}{v_{P_{a,2}}(x)}\right]\right]_{x=0,1} = 0, \quad \left[v_{P_{r,2}}^2(x)\frac{d}{dx}\left[\frac{P_{r,2}(t,x)}{v_{P_{r,2}}(x)}\right]\right]_{x=0,1} = 0.
$$

**Parameters for Figure 7 in the Main Text**: The growth rate we used to nondimensionalize the time scales was $\mu = 0.5$ 1/hr, consistent with the experiments [10]. The length of the cell is about $3\mu$m and thus $L = 1.5\mu$m [8]. The diffusion coefficient of the transcription factor is taken to be $D_a = D_r = 0.4\mu m^2/s$ (that of LacI) [13] and thus $\chi_a = \chi_r = 1280$. The following dimensionless parameters were chosen such that the well-mixed model displayed sustained oscillations: $a_1 = 220, d_1 = 1000, a_2 = 1000, d_2 = 1000, a_3 = 1000, d_3 = 1000, a_4 = 1000, d_4 = 1000, a_5 = 1000, d_5 = 1000, \kappa_3 = 250, \kappa_1 = .04, \kappa_4 = 30, \kappa_2 = .004, \gamma_a = 1, \gamma_r = 0.5$. Furthermore, we choose $d_i$ and $a_i$ for $i = 1, \dots 5$ large, to demonstrate our results hold even for large binding and unbinding rates. The total concentration of $\mathrm{D_a}$ which is the same as $\mathrm{D_r}$ since we assume they are on the same plasmid, is given by $\bar{D}_T$ and it was used to nondimensionalize the other concentration variables such that $\bar{D}_T = 1$. The total DNA spatial profile was chosen as $D_T(x) \propto e^{50(x-1)}$ to model DNA at cell poles.

# 6 Numerical Method

In general, a closed form solution to nonlinear PDEs appearing in this work (e.g., Equation 4 in the Main Text) is not available. Therefore, we rely on numerical solutions to directly integrate the PDE. In particular, we utilize a finite difference method that is widely used to simulate PDEs [20]. For a general diffusing species with its concentration given by $y(t,x)$, and available volume profile $v(x)$, we make the following coordinate transformation $u(t,x) := y(t,x)/v(x)$. In this coordinate, the boundary conditions (if applicable) are Neumann [21]: $\frac{\partial u}{\partial x}\big|_{x=0,1} = 0$ and thus are simpler to implement. Furthermore, with this transformation we observe less stiffness in the numerical simulations. We discretized the spatial domain into $N+1$ equidistant points such that $\Delta = 1/N$. Using a second order finite difference method, we approximate the derivatives as:

$$
\frac{\partial u}{\partial x}\bigg|_{x=x_i} \approx \frac{u(t,x_{i+1}) - u(t,x_{i-1})}{2\Delta} \quad \text{and} \quad \frac{\partial^2 u}{\partial x^2}\bigg|_{x=x_i} \approx \frac{u(t,x_{i+1}) - 2u(t,x_i) + u(t,x_{i-1})}{\Delta^2},
$$

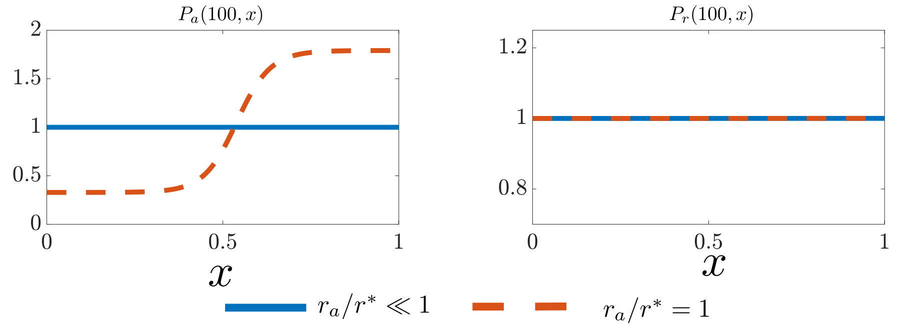


Figure M: **The activator is excluded from the chromosome as its size increases** The steady state spatial profiles normalized by the average values for $\mathrm{P_a}$ and $\mathrm{P_r}$, that is $P_a(\infty, x)/\bar{P}_a(\infty)$ and $P_r(\infty, x)/\bar{P}_a(\infty)$ for the results of Figure 7 in the Main Text. As the size of $\mathrm{P_a}$ increases it is excluded from the chromosome. The repressor remains homogeneously distributed throughout the cell. The parameter values and simulation details are identical to those of Figure 7 in the Main Text.

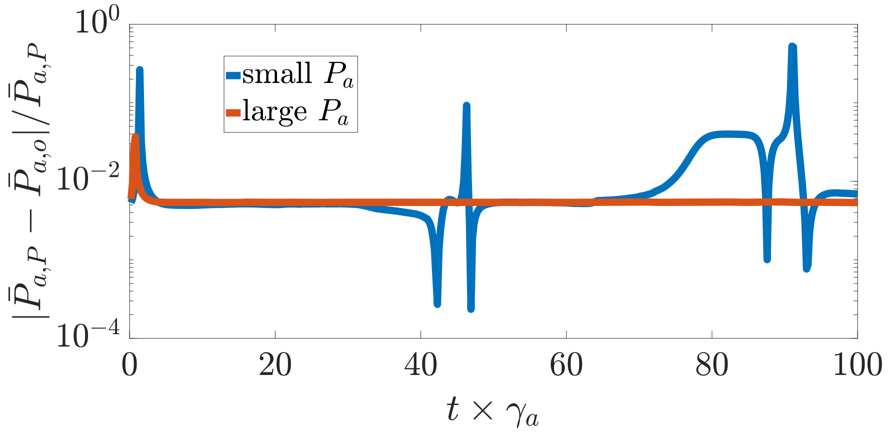


Figure N: **The relative error between the space averaged PDE model and the reduced ODE model from the data in Figure 7 in the Main Text** The relative error in the steady state space averaged activator protein concentration for the full PDE model ($\bar{P}_{a,P}$) and the reduced model ($\bar{P}_{a,o}$) from the data in Figure 7 in the Main Text. Note that for the case when $\mathrm{P_a}$ is small, large relative errors occur near when $\bar{P}_a(t)$ reaches a minimum during each period of oscillation. Otherwise all errors are less than 1%.

that appear in the flux terms (in the case where species freely diffuses). The new boundary conditions give rise to the following constraints:

$$\left.\frac{\partial^2 u}{\partial x^2}\right|_{x=0} \approx 2\frac{u(t, x_2) - u(t, 0)}{\Delta^2} \quad \text{and} \quad \left.\frac{\partial^2 u}{\partial x^2}\right|_{x=1} \approx 2\frac{u(t, x_N) - u(t, 1)}{\Delta^2}.$$

This discretization leads to a system of $N + 1$ ODEs. The resulting set of ODEs are then simulated with MATLAB, using the numerical ODE solvers ode23s. To calculate the space averaged concentrations, we implement a second oder trapezoidal integration scheme [21] given by:

$$\bar{y}(t) := \int_0^1 y(t, x)dx, \quad \bar{y}(t) \approx \frac{\Delta}{2}\sum_{j=1}^{N}\left[y(t, x_j) + y(t, x_{j+1})\right].$$

For all simulations in this paper $N = 200$.

Now we demonstrate the convergence rate of our numerical method. For the simulation in Figure 5-A in the Main Text when the DNA is localize at the cell-poles and $r_s/r^* = 1$, we varied the number of spatial nodes used to discretized the spatial domain to demonstrate the convergence rate of our numerical scheme. Let $N$ be the number of points used to discretize the spatial domain and let $\bar{m}_N(t)$ as the space averaged

mRNA concentration for that given discretization. We considered when $N = 1024$ to be the true solution $m_{1024}(t)$ and thus define the following relative error

$$e_\infty = \max_{t\in[0,T]} \left| \frac{\bar{m}_N(t) - \bar{m}_{1024}(t)}{\bar{m}_{1024}(t)} \right|, \tag{45}$$

where we took its max value over the time interval of the simulation $t \in [0, T]$ where $T = 100$. The results from this numerical experiment are shown in Figure O. The convergence rate of our numerical scheme is $\mathcal{O}(N^{-2})$ as expected for a second order finite difference numerical scheme.

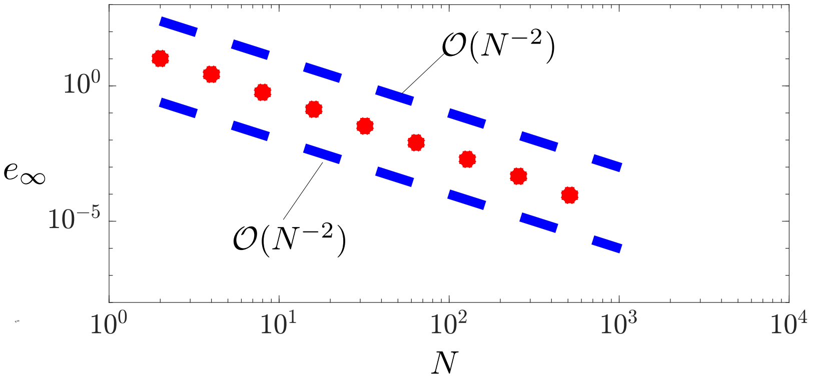


Figure O: **Convergence rate of numerical scheme used to simulate PDEs in this work** For the simulation in Figure 5-A in the Main Text when the DNA is localize at the cell-poles and $r_s/r^* = 1$, we varied the number of spatial nodes used to discretize the spatial domain to demonstrate the convergence rate of our numerical scheme. Let $N$ be the number of points used to discretize the spatial domain and let $\bar{m}_N(t)$ as the space average mRNA concentration for that given discretization. We considered when $N = 1024$ to be the true solution $m_{1024}(t)$ and the relative error $e_\infty$ is given by (45). The relative error is given by the red markers and the blue dashed lines serve as references for $\mathcal{O}(N^{-2})$ convergence rates.

# 7 Estimating $r^*$ from Concentration Profiles and Estimating the BFC

**Estimate $r^*$:** As discussed in Remark 1 in the Main Text, we expect the concentration profile of a freely diffusing species to mirror that of the normalized available volume profile. That is, for a freely diffusing species y, with concentration $y(t, x)$, and available volume profile $v(x)$, we expect

$$y(t,x) \approx \bar{y}(t)\hat{v}(x) \quad \text{where} \quad \bar{y}(t) = \int_0^1 y(t,x)dx, \quad \text{and} \quad \hat{v}(x) = \frac{v(x)}{\int_0^1 v(x)dx}. \tag{46}$$

Suppose the radius of gyration of y denoted by $r$, is known and as discussed in the Main Text, we have that $v(x) = e^{-(r/r^*)^2\hat{\rho}(x)}$. Approximating $\hat{\rho}(x)$ as a step function as in (31), we have that $v(1) \approx 1$ and $v(0) = e^{-\frac{(r/r^*)^2}{2(1-\Delta x)}}$, where $\Delta x$ is the distance between the end of the chromosome and the cell poles (see Figure 1 in the Main Text). Let $y^{\text{in}}$ and $y^{\text{out}}$ denote the the average concentration inside and outside the nucleoid, respectively, which are given by

$$y^{\text{in}} = \frac{1}{1-\Delta x}\int_0^{1-\Delta x} y(x)dx, \quad y^{\text{out}} = \frac{1}{\Delta x}\int_{1-\Delta x}^1 y(x)dx.$$

Let $\psi_y = y^{\text{out}}/y^{\text{in}}$ and from (46), we have that

$$\psi_y = y^{\text{out}}/y^{\text{in}} = v(0)/v(1) = v(0) = e^{-\frac{(r/r^*)^2}{2(1-\Delta x)}}, \tag{47}$$

then $r^*$ can be estimated assuming one knows $\Delta x$, that is, how far the dense nucleoid region extends beyond mid-cell and the average concentration of a species inside and outside nucleoid region. A similar calculation was done in [8], using the fact that the free ribosome concentration is 10% higher at the cell poles than mid-cell.

**Estimate the BCF:** The BCF provides a measure to determine the extent to which spatial effects modulate the biomolecular dynamics. Therefore, an experimental method to estimate the BCF is desirable. We propose a method that only requires knowing $\Delta x$ and the concentration of freely diffusing species inside and outside the nucleoid .

Suppose that for Case I and Case III, the concentration of $\mathrm{E_i}$ is measured inside and outside the nucleoid and denoted by $E_i^{\text{in}}$ and $E_i^{\text{out}}$, respectively. Similarly, for Case I and Case II, we assume that $S^{\text{in}}$ and $S^{\text{out}}$ is measured. If a fluorescence imaging method is used to measure these quantities (as in [9]), then we emphasize that the free $\mathrm{E_i}$ and S must be measured, not when they are in complex form ($\mathrm{c_i}$). Let $\psi_i = E_i^{\text{out}}/E_i^{\text{in}}$ and $\psi_s = S^{\text{out}}/S^{\text{in}}$. By (47), $\psi_i = v_{0,E_i}$ and $\psi_s = v_{0,S}$, where $v_{0,E_i}$ and $v_{0,S}$ are as in (32). Thus, using (33) we can estimate the BCF for Cases I-III by

- Case I:

$$\theta_i^* = \frac{\psi_i \psi_s (1-\Delta x) + \Delta x}{\left[\psi_i(1-\Delta x)+\Delta x\right]\left[\psi_s(1-\Delta x)+\Delta x\right]},$$

- Case II and Case III:

$$a_i = \begin{cases} \psi_s, & \text{Case II} \\ \psi_i, & \text{Case III} \end{cases},$$

$$\theta_i^* = \begin{cases} 0 \leq \dfrac{a_i}{a_i(1-\Delta x)+\Delta x} \leq 1, & x_i^* \leq 1-\Delta x \text{ for Case II and } x_s^* \leq 1-\Delta x \text{ for Case III} \\ 1 \leq \dfrac{1}{a_i(1-\Delta x)+\Delta x} \leq \dfrac{1}{\Delta x} & x_i^* > 1-\Delta x \text{ for Case II and } x_s^* > 1-\Delta x \text{ for Case III.} \end{cases}$$

As $r_{e,i}/r^* \to \infty$ ($r_s/r^* \to \infty$) we have that $\psi_i \to 0$ ($\psi_s \to 0$), thus $\psi_i$ and $\psi_s$ are a measure of the excluded volume effects on $\mathrm{E_i}$ and S, respectively. Physically, this is expected because when $\mathrm{E_i}$ is severely expelled from the nucleoid by available volume effects, we have that $E_i^{\text{out}} \ll E_i^{\text{in}}$ and similarly for S.

# 8 Experimental Setups to Verify the Role of Spatial Effects Predicted by Model

A potential experiment to test our hypothesis that genes near the poles our transcribed more effectively than gene near mid-cell, is to measure the rate of transcription (via Quantitative PCR) of a gene under the control of the T7 promoter. This promoter is solely transcribed by the T7 RNAP which specifically targets the promoter, thus this system can be considered orthogonal to the endogenous transcription machinery [22]. By appending random base pairs (BPs) to the sequence of T7 RNAP that do not affect its functionally, we can control its size and thus the extent of excluded volume effects. We can then measure the transcription rate of the gene regulate by the T7 promoter when it is localized in the cell-poles and mid-cell. The results of this experiment should look similar to Figure 5-A in the Main Text, as the size of the T7 RNAP increases the mid-cell (pole) gene has lower (higher) transcription rate. For the mid-cell localized gene, this can be repeated in parts of the chromosome which are known to be dense to amplify these effects.

To experientially validate our analytical prediction that protein steady state levels will increase with mRNA size, we propose expressing a florescence protein from a plasmid with an appended sequence of base pairs added downstream of the stop codon. The appended sequence should have a low affinity to recruit ribosome such that the amount of ribosomes sequestered by the mRNA are the same as without the appended sequence. Assuming this appended sequence does not affect the lifetime of the mRNA, then it should yield the same functional protein which can be used to quantify the mRNA excluded volume effects. This appended sequence of base pairs will allow us to control the size of the mRNA without increasing its ribosome usage. From our theory, for longer appended sequences, more protein expression is expected.

To validate the hypothesis that a transcriptional repressor regulates genes near the poles more effectively than gene near mid-cell, we propose a genetic circuit on a plasmid expressing a repressor that targets a gene expressing a florescence protein. The transcription factor chosen should be large enough or dimerize to have

considerate excluded volume effects. The target DNA expressing protein should be placed on several axial locations in the cell (under the same promoter) achieved by using backbones with different localization profiles and/or different chromosomal integration sites. We should observe that the effective disassociation constant of the repression curve increases as the target genes location is closer to the mid-cell. The disassociation constant is proportional to the amount of repressor necessary to cause the genes expression to decrease by half.

# 9 Cell Division: Time Varying Cell Length and Chromosome Profile

As the cell divides it partitions molecular species amongst daughter cells, this along with changes in the cell length cause dilution effects on intracellular concentrations. Furthermore, early in the cell division cycle, the chromosome density is highest mid-cell, but as the cell divides the peak chromosome density tends towards the cell-poles [9, 8] (to distribute genes evenly among daughter cells). From the results in the Main Text, we expect this temporal changes in the chromosome density will effect the BCF since species are repelled away from regions with high chromosome density via excluded volume effects. In this section, we provide the modeling framework to account for dilution effects and temporal fluctuations in the chromosome density.

**Dilution effects on spaced average concentrations:** Here we demonstrate how cell division and a time varying cell length effects space average concentrations. Let $\bar{N}_p(t)$ be the total molecular count in a cell population of a molecule of interest (i.e., ribosomes) as the cell expands and divides. To model dilution from cell division, assume that $\bar{N}_p(t)$ is identically distribute among $N_{\text{cells}}(t)$ number of cells such that $N_{\text{cells}}(t) = N_{\text{cells}}(0)e^{\mu t}$, where $\mu$ is the cell growth rate and each cell has a volume given by $V_c(t) = 2\pi R^2 L(t)$, where $R$ is the cell radius and $2L(t)$ is the cell length. The total population volume is then given by $V(t) = N_c(t)V_c(t)$ and letting $\bar{c}(t)$ be the number of molecules per total volume (concentration), this quantity is given by $\bar{c}(t) = \frac{\bar{N}_p(t)}{V(t)}$, note that this is identical to the concentration per cell volume (since we assume all cells in the population have identical averaged concentrations). This implies that

$$\dot{\bar{c}}(t) = \frac{\dot{\bar{N}}_p(t)}{V(t)} - \underbrace{\frac{\dot{V}(t)}{V(t)}\bar{c}(t)}_{\text{dilution effects}} = \frac{\dot{\bar{N}}_p(t)}{V(t)} - (\underbrace{\mu}_{\text{cell division}} + \underbrace{\frac{\dot{L}}{L}}_{\text{varying length}})\bar{c}(t).$$

**Dilution effects on local concentrations:** Let $N(t,x)$ be the number of molecules per unit length of a cell such that that $\int_0^{L(t)} N(t,x)dx = \frac{\bar{N}_p(t)}{N_{\text{cells}}(t)}$. The temporal evolution of $N(t,x)$ in the presence of dilution and a moving boundary, which introduces an advective term [23] (to account for the extra diffusion as the cell length varies), is given by

$$\frac{\partial N(t,x)}{\partial t} = \underbrace{D\frac{d}{dx}\big[v^2(x)\frac{d}{dx}[\frac{N(t,x)}{v(x)}]\big]}_{\text{diffusion}} \underbrace{-\frac{d}{dx}(u(t,x)N(t,x))}_{\text{advection}} + \underbrace{f(t,x,N)}_{\text{reaction}} - \underbrace{\mu N(t,x)}_{\text{dilution}}, \tag{49}$$

with boundary conditions

$$D\Big[v^2(x)\frac{d}{dx}[\frac{N(t,x)}{v(x)}]\Big]_{x=0,x=L(t)} = 0,$$

where $u(t,x) = \frac{x}{L(t)}\dot{L}(t)$ is the velocity of a material point induced by the increase in cell length. Notice that our current finite difference method with a stationary mesh cannot be applied directly to (49), thus we propose the following spatial coordinate transformation $y(t,x) = \frac{x}{L(t)}$ (and to be consistent with the nondimensionalization from the Main Text), which renders a stationary domain. Let $c(t,y) := N(t, yL(t))$ and thus $\frac{dN}{dt} = \frac{dc}{dt} = \frac{\partial c}{\partial t} + \frac{\partial c}{\partial y}\frac{\partial y}{\partial t} = \frac{\partial c}{\partial t} - \frac{\partial c}{\partial y}y\frac{\dot{L}}{L}$, finally

$$\begin{aligned}\frac{\partial c}{\partial t} =& \frac{\partial c}{\partial y}y\frac{\dot{L}}{L} + \frac{D}{L^2(t)}\frac{d}{dy}\big[v^2(y)\frac{d}{dy}[\frac{c}{v(y)}]\big] - \frac{1}{L(t)}\frac{d}{dy}(uc) + f(t,c) - \mu c,\\ =& \frac{D}{L^2(t)}\frac{d}{dy}\big[v^2(y)\frac{d}{dy}[\frac{c}{v(y)}]\big] + f(t,c) - (\mu + \frac{\dot{L}}{L})c.\end{aligned}$$

Notice that the effective dilution coefficient is now given by $\frac{D}{L^2(t)}$, which as expected increases as cell length increases and $2\int_0^1 c(t,y)dy = 2\int_0^{L(t)} \frac{N(t,x)}{L(t)}dx = \dot{\bar{c}}(t)$, thus the space averaged under this coordinate system provides us the concentration per cell volume. The boundary conditions are

$$D\left[v^2(y)\frac{d}{dy}[\frac{c(t,y)}{v(y)}]\right]_{y=0,y=1} = 0.$$

**Time varying chromosome density:** We now model the chromosome density varying in time $\hat{\rho} := \hat{\rho}(t,x)$ as the cell divides. This implies that the available volume profiles will also depend on time since $v(x,t) = e^{-\frac{r}{r^*}\hat{\rho}(x,t)}$, and thus

$$\frac{\partial c}{\partial t} = \frac{D}{L^2(t)}\frac{d}{dy}\left[v^2(t,y)\frac{d}{dy}[\frac{c}{v(t,y)}]\right] + f(t,c) - \tilde{\mu}(t)c,$$

where $\tilde{\mu}(t)$ is the effective dilution rate given by

$$\tilde{\mu}(t) = \mu + \frac{\dot{L}(t)}{L(t)} \tag{50}$$

The quantities $L(t)$ and $v(t,x)$ will vary with a time scale related to cell growth, for example, let $T_{1/2} = \frac{\ln(2)}{\mu}$ be the cell doubling time, then one possibility is

$$L(t) = L_0(1 - \Delta_L \cos(2\pi t/T_{1/2})), \tag{51}$$

for this choice of $L(t)$, the effective dilution rate (50) is graphically shown in Figure P. In [8] it was shown the cell length late in the cell division cycle was $4.4\mu m$ (compare to its nominal length $3\mu m$), thus for our simulations we take $\Delta_L = 0.2$.

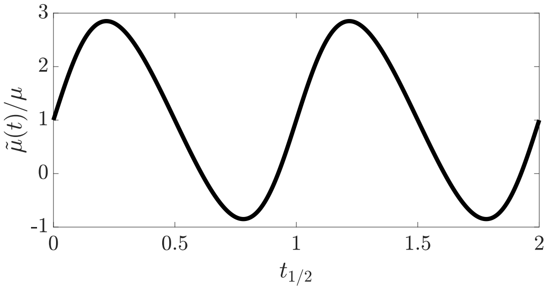


Figure P: **Varying cell length modulates dilution rate** The effective dilution rate (50) is given for $L(t)$ given by (51) and $\Delta_L = 0.2$, where $t_{1/2}$ is time normalized by the cell doubling time ($t_{1/2} = t/T_{1/2}$).

In [9] it was experimentally shown how the chromosome density varies with time and a model for the density in early ($\hat{\rho}_1(y)$) and late ($\hat{\rho}_2(y)$) in the cell division process were provided in [8]

$$\hat{\rho}_1(y) = c_1\frac{1}{1+e^{20(y-1/2)}} \quad \hat{\rho}_2(y) = c_2\frac{e^{-6(1/2-y)^2} + e^{-6(1/2+y)^2}}{1+e^{20(y-2/3)}}$$

where $c_1$ and $c_2$ are chosen such that $\int_0^1 \hat{\rho}_1 dy = \int_0^1 \hat{\rho}_2 dy = 1/2$. To capture the transition between $\hat{\rho}_1$ and $\hat{\rho}_2$ as the cell divides we propose the following model

$$\hat{\rho}(t,y) = 1/2\left(\hat{\rho}_1(y)\cos^2(\pi t/T_{1/2}) + \hat{\rho}_2(y)\sin^2(\pi t/T_{1/2})\right), \tag{52}$$

notice that $\int_0^1 \rho(t,y)dy = 1/2, \forall t \geq 0$. The model for the cell length and the chromosome density is shown in Figure Q. The model for the chromosome density is consistent with experimental observations where late in the division phase the chromosome is in the form of two lobes, where each lobe of DNA will correspond to a daughter cell.

**Time scale separation:** When the scale associated with diffusion is much fast than dilution $D/L_0 \gg \mu$ (and any other time scale associated with the reaction dynamics), we can treat $L(t)$ and $v(t,y)$ as constant

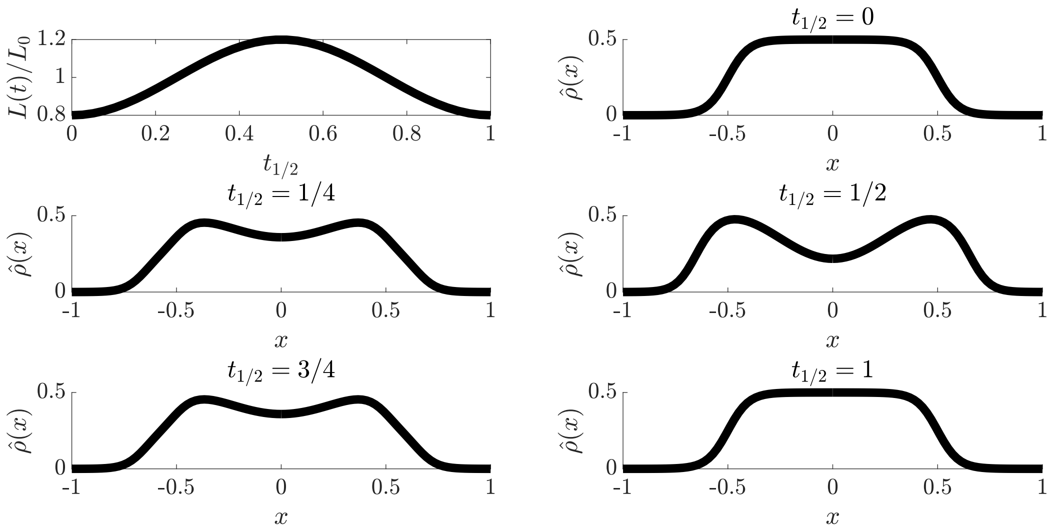


Figure Q: **The cell length and chromosome density varies over time** The normalized cell length $L(t)/L_0$ (51) with $\Delta_L = 0.2$ and the chromosome density $\hat{\rho}(t, y)$ (52) shown over one cell division cycle, where $t_{1/2}$ is time normalized by the cell doubling time. The model for the chromosome density is consistent with experimental observations [9, 8] where late in the division phase the chromosome is in the form of two lobes, where each lobe of DNA will correspond to a daughter cell.

in time when performing model reduction as in the "Time scale separation" section in the Main Text, thus we expect (similar to the results of the Main Text)

$$c(t, y) \approx \bar{c}(t)\hat{v}(t, y), \tag{53}$$

where $\bar{c}(t) = \int_0^1 c(t, y)dy$ and $\hat{v}(t, y) = v(t, y)/\int_0^1 v(t, y)dy$. So all of our previous analysis still holds except that the BCF will vary slowly (slow with respect to the time scale of diffusion) as the cell divides.
**Example:** We verify via simulations the prediction that (53) holds and that the BCF can be treated as a slowly (with respect to diffusion) varying parameter. Consider the simple bimolecular reaction:

$$\mathrm{E} + \mathrm{S} \underset{\mathrm{d}}{\overset{\mathrm{a}}{\rightleftharpoons}} \mathrm{c}, \quad \emptyset \xrightarrow{\alpha_e} \mathrm{E}, \qquad \emptyset \xrightarrow{\alpha_s} \mathrm{S}, \tag{54}$$

with dynamics given by

$$\begin{aligned}
\frac{\partial E(t, y)}{\partial t} &= \frac{D_e}{L^2(t)}\frac{d}{dy}\left[v_e^2(t, y)\frac{d}{dy}\left[\frac{E(t, y)}{v_e(t, y)}\right]\right] + \alpha_e(y) - aE(t, y)S(t, y) + dc(t, y) - \tilde{\mu}(t)E(t, y), \\
\frac{\partial S(t, y)}{\partial t} &= \frac{D_s}{L^2(t)}\frac{d}{dy}\left[v_s^2(t, y)\frac{d}{dy}\left[\frac{S(t, y)}{v_s(t, y)}\right]\right] + \alpha_s(y) - aE(t, y)S(t, y) + dc(t, y) - \tilde{\mu}(t)S(t, y), \\
\frac{\partial c(t, y)}{\partial t} &= \frac{D_c}{L^2(t)}\frac{d}{dy}\left[v_c^2(t, y)\frac{d}{dy}\left[\frac{c(t, y)}{v_c(t, y)}\right]\right] + aE(t, y)S(t, y) - \tilde{\mu}(t)c(t, y),
\end{aligned} \tag{55}$$

where $\alpha_e(y)$ and $\alpha_s(y)$ are the production rates of E and S, respectively. The space averaged dynamics ($\bar{E}(t) = \int_0^1 E(t, y)dy$, $\bar{S}(t) = \int_0^1 S(t, y)dy$, and $\bar{c}(t) = \int_0^1 c(t, y)dy$) are given by

$$\begin{aligned}
\frac{d\bar{E}(t)}{dt} &= \bar{\alpha}_e - a\theta(t)\bar{E}(t)\bar{S}(t) + d\bar{c}(t) - \tilde{\mu}(t)\bar{E}(t), \\
\frac{d\bar{S}(t)}{dt} &= \bar{\alpha}_s - a\theta(t)\bar{E}(t)\bar{S}(t) + d\bar{c}(t) - \tilde{\mu}(t)\bar{S}(t), \\
\frac{d\bar{c}(t)}{dt} &= a\theta(t)\bar{E}(t)\bar{S}(t) - \tilde{\mu}(t)\bar{c}(t),
\end{aligned} \tag{56}$$

where the BCF is given by

$$\theta(t) = \frac{\int_0^1 E(t,y)S(t,y)dy}{\left[\int_0^1 E(t,y)dy\right]\left[\int_0^1 S(t,y)dy\right]}. \tag{57}$$

We first focus on the $E(t,y)$ dynamics when $a = 0$ and $d = 0$

$$\frac{\partial E(t,y)}{\partial t} = \frac{D_e}{L^2(t)}\frac{d}{dy}\left[v_e^2(t,y)\frac{d}{dy}\left[\frac{E(t,y)}{v_e(t,y)}\right]\right] + \alpha_e(y) - \tilde{\mu}(t)E(t,y) \tag{58}$$

to show the effects of having time varying dilution $\tilde{\mu}(t)$ and available volume profiles on the expression level. Figure R shows how the space averaged concentration $\bar{E}(t)$ is modulated by the time varying dilution $\tilde{\mu}(t)$. We observe that the concentration reaches a periodic steady state centered at unity where the oscillations have a period that coincides with the doubling time. Furthermore, in Figure S, we verify that

$$E(t,x) \approx \bar{E}(t)\hat{v}_e(t,y)$$

where $\hat{v}_e(t,y) = v_e(t,y)/\int_0^1 v_e(t,y)dy$ as expected (since diffusion much faster than dilution $D_e/(L_0\mu) \gg 1$). Thus, even with a time varying $v_e(t,y)$, the enzyme will be expelled from the chromosome to areas of higher available volume.

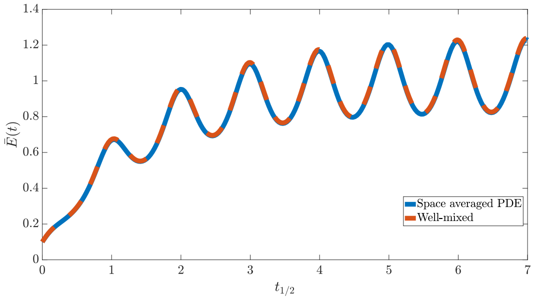


Figure R: **Enzyme expression as cell length varies** The space averaged enzyme expression (58) there is no binding/unbinding with S ($a = 0$ and $d = 0$). The oscillations arise due to changes in the cell length during cell division. The simulation parameters are: $r_e/r^* = 2$ $\mu = 1$ $D_e/(L_0\mu) = 13 \times 10^3$ $\alpha_e(y) = 1$, $\Delta_l = 0.2$.

Next, we demonstrate how the binding dynamics are effected by having a time varying available volume profile, therefore $a \neq 0$ in (55). Similar to "Case 1" in the Main Text, we consider the case when $D_e, D_s, D_c \neq 0$ (all species freely diffuse), where we expect the BCF to be approximated by

$$\theta^*(t) = \frac{\int_0^1 v_e(t,y)v_s(t,y)dy}{\left[\int_0^1 v_e(t,y)dy\right]\left[\int_0^1 v_s(t,y)dy\right]}. \tag{59}$$

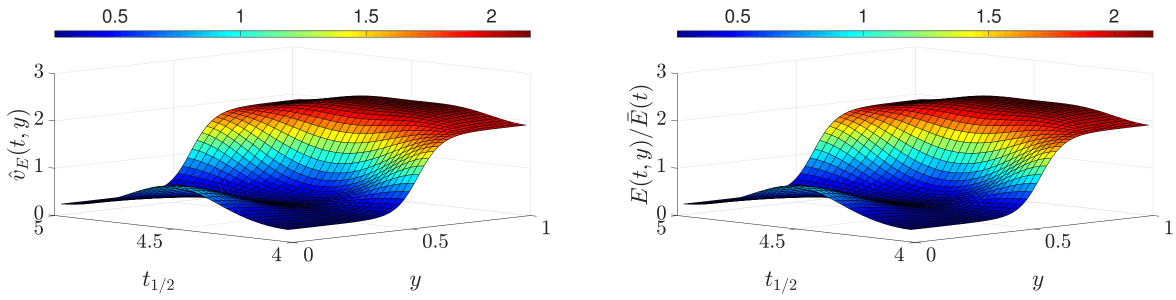


(a) Normalized available volume profile

(b) Normalized enzyme concentration

Figure S: **The normalized enzyme spatial profile matches that of its normalized available volume profile.** The enzyme concentration spatial profile normalized by its space average shown over one cell division cycle after four cell division cycles ("steady state") matches its available volume as expected (58). The simulation parameters are: $r_e/r^* = 2$ $\mu = 1$ $D_e/(L_0\mu) = 13 \times 10^3$ $\alpha_e(y) = 1$, $\Delta_l = 0.2$.

This is verified in Figure T, where $\theta(t)$ given by (57) and $\theta^*(t)$ given by (59) are shown after three doubling times and are shown to be in good agreement. The BCF varies periodically in time (with the period consistent with the doubling time) and oscillates near a nominal value of 1.5 with amplitude 0.04.

Next we look at the the case when S and c are spatially fixed ($D_s = D_c = 0$) and localized near $y^*$, which is similar to "Case 2" in the Main Text. For this scenario we expect the BCF to be approximated by

$$\theta^*(t) = \hat{v}_s(t, y^*). \tag{60}$$

The results are shown in Figure U when $y^* = 0$ and $y^* = 1$ after three doubling times, there is good agreement between the BCF and its approximation. When $y^* = 0$ (S localized near mid-cell), the BCF is less than unity and oscillates near a nominal value of 0.55 with amplitude 0.3. When $y^* = 1$ (S localized near the cell poles), the BCF is greater than unity and oscillates near a nominal value of 2 with amplitude 0.1.

These results suggest that the BCF for a species localized near mid-cell will vary significantly as the cell density varies during cell division. This is expected because as shown in Figure 1 in the Main Text, the chromosome density is initially high near mid-cell but decreases by half as the cell divides, thus no longer excluding from that region.

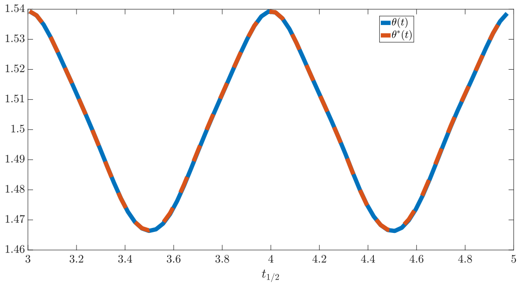


Figure T: **The BCF for the case when all species freely diffuse** The binding correction factor $\theta(t)$ (57) and its approximation $\theta^*$ (59) over two cell division cycles. The BCF oscillates around a nominal value of 1.5 with amplitude 0.04 and period consistent with the doubling time. The simulation parameters are $r_e/r^* = r_s/r^* = 2$, $r_c/r^* = 2\sqrt{2}$, $\mu = 1$, $D_e/L_0 = D_s/L_0 = D_c/L_0 = 13 \times 10^3$ $\alpha_e(y) = \alpha_s(y) = 1$, $\Delta_l = 0.2$, $d = 100$, $a = 100$.

## 10 Exclusion Effects from Plasmid DNA Density

The genome of *E. coli* MG1655 has 4.6 Mbp [24]. Comparatively, a single plasmid can have .01 Mbp a copy number as high as 500-700 (e.g. pUC19). Therefore, the total plasmid and chromosome basepair count may be comparable in applications with high copy number plasmids. In these applications, it may be necessary to account how plasmid DNA repels freely diffusing species and "excludes" them. To do so we modify our model of the DNA density $\hat{\rho}(x)$ as shown in Figure V to account for plasmid DNA. For the DNA density profiles from Figure V, we calculate the approximate BCF $\theta^*$ (Equation 5 in the Main Text), these are shown in Figure W. For Case 1 where the reactants freely diffuse. We observe that the BCF decreases as the plasmid DNA density increases (as shown in Figure V). This occurs because as the plasmid DNA increases the overall density profile becomes more uniform. Note that when the plasmid DNA is sufficiently high to render an almost uniform DNA density profile, the BCF is unity as expected. For Case 2, where one reactant freely diffuses and the other is fixed at $x^*$. As the plasmid density increases (as shown in Figure V) we observe that the BCF decreases at the cell poles (as expected since species are excluded from dense plasmid DNA mesh) and increases at region near $x^* \approx 0.65$ (where there is minimal overlap between chromosome and plasmid DNA). When the plasmid DNA density is similar to that of the chromosome rending a uniform DNA distribution, we observe that the BCF is unity everywhere (as expected since there are no exclusion effects).

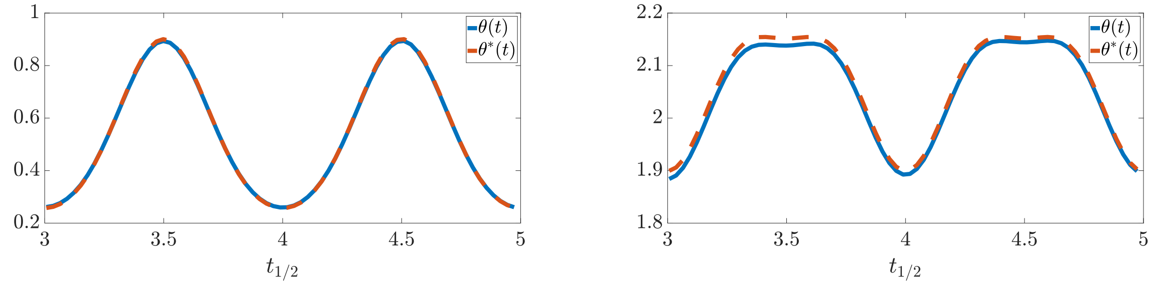


(a) $y^* = 0$ (b) $y^* = 1$

Figure U: **The BCF for the case when S is spatially fixed at** $y^*$ The binding correction factor $\theta(t)$ (57) and its approximation $\theta^*$ (60) over two cell division cycles. The BCF oscillates with a period consistent with the doubling time. When $y^* = 0$ (S localized near mid-cell), the BCF is less than unity and oscillates near a nominal value of 0.55 with amplitude 0.3. When $y^* = 1$ (S localized near the cell poles), the BCF is greater than unity and oscillates near a nominal value of 2 with amplitude 0.1. This is shown for a molecule localized near mid-cell $y^* = 0$ and near the cell poles $y^* = 1$. The simulation parameters are $r_e/r^* = r_s/r^* = 2$, $r_c/r^* = 2\sqrt{2}$, $\mu = 1$, $D_e/L_0 = D_s/L_0 = D_c/L_0 = 13 \times 10^3$ $\alpha_e(y) = \alpha_s(y) = 1$, $\Delta_l = 0.2$, $d = 100$, $a = 100$.

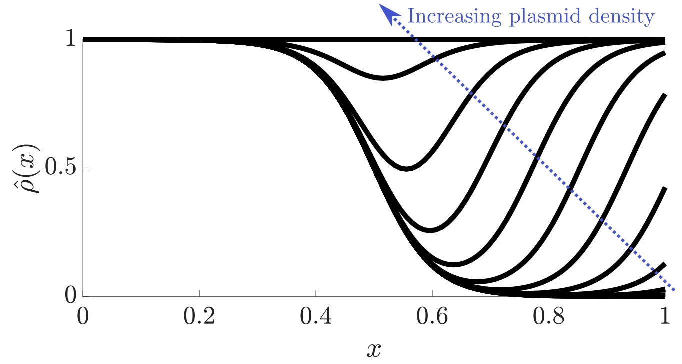


Figure V: **DNA density with plasmid contributions**. The DNA density now includes contributions from plasmid DNA. We show several profiles with increasing plasmid density. For this results we had $\hat{\rho}(x) = \frac{1}{1+e^{20(x-1/2)}} + \frac{1}{1+e^{20(-x+x_s)}}$ where $x_s \in [1/2, 3/2]$ is the parameter we varied to get difference plasmid densities ($x_s = 3/2$ lowest plasmid density and $x_s = 1/2$ highest plasmid density).

# 11 Codes

A copy of the codes used in this study can be found at:
https://drive.google.com/drive/folders/11tJ9rn8QLs-G2xOsOhpXnOSqPJvI804y?usp=sharing

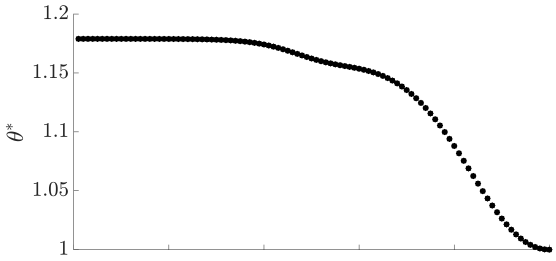


(a) Case 1: Reactants freely diffuse

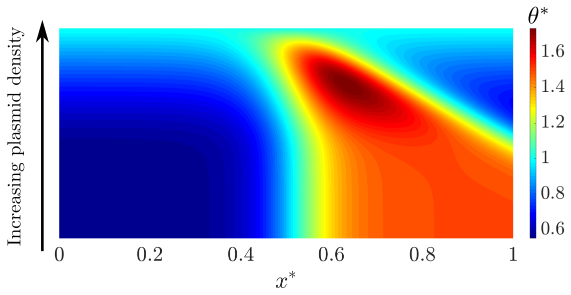


(b) Case 2: One reactant diffuses and the other fixed at $x^*$

Figure W: **The approximate BCF $\theta^*$ when plasmid DNA is accounted for** (a) Case 1 where the reactants freely diffuse. We observe that the BCF decreases as the plasmid DNA density increases (as shown in Figure V). This occurs because as the plasmid DNA increases the overall density profile becomes more uniform. Note that when the plasmid DNA is sufficiently high to render an almost uniform DNA density profile, the BCF is unity as expected. (b) Case 2 where one reactant freely diffuses and the other is fixed at $x^*$. As the plasmid density increases (as shown in Figure V) we observe that the BCF decreases at the cell poles (as expected since species are excluded from dense plasmid DNA mesh) and increases at region near $x^* \approx 0.65$ (where there is minimal overlap between chromosome and plasmid DNA). When the plasmid DNA density is similar to that of the chromosome rending a uniform DNA distribution, we observe that the BCF is unity everywhere (as expected since there are no exclusion effects). The simulation parameters are $r/r^* = 1$ and $\hat{\rho}(x)$ as in Figure V.

# References


[1] Renardy M, Rogers RC. An Introduction to Partial Differential Equations. Differential Equations. 2003;13(2):1–5. doi:10.2307/2312165.

[2] Lohmiller W, Slotine JJE. On Contraction Analysis for Non-linear Systems. Automatica. 1998;34(6):683–696. doi:10.1016/S0005-1098(98)00019-3.

[3] Russo G, Slotine JJE. Symmetries, stability, and control in nonlinear systems and networks. Physical Review E - Statistical, Nonlinear, and Soft Matter Physics. 2011;84(4). doi:10.1103/PhysRevE.84.041929.

[4] Horn RA, Johnson CR. Matrix Analysis. Cambridge: Cambridge University Press; 2012. Available from: http://ebooks.cambridge.org/ref/id/CBO9781139020411.

[5] Weinberger H. Invariant sets for weakly coupled parabolic and elliptic systems. Rend Mat Univ Roma. 1975;8(l):295–310.

[6] Trench WF. Trench_Real_Analysis; 2010. Available from: papers2://publication/uuid/618DB599-A621-415F-A7CA-2269F9743C0F.

[7] Alon U. An Introduction to Systems Biology: Design Principles of Biological Circuits; 2007.

[8] Castellana M, Hsin-Jung Li S, Wingreen NS. Spatial organization of bacterial transcription and translation. Proceedings of the National Academy of Sciences. 2016;113(33):9286–9291. doi:10.1073/pnas.1604995113.

[9] Bakshi S, Siryaporn A, Goulian M, Weisshaar JC. Superresolution imaging of ribosomes and RNA polymerase in live Escherichia coli cells. Molecular Microbiology. 2012;85(1):21–38. doi:10.1111/j.1365-2958.2012.08081.x.

[10] Gyorgy A, Jiménez JI, Yazbek J, Huang HH, Chung H, Weiss R, et al. Isocost Lines Describe the Cellular Economy of Genetic Circuits. Biophysical Journal. 2015;109(3):639–646. doi:10.1016/j.bpj.2015.06.034.

[11] Bratton BP, Mooney RA, Weisshaar JC. Spatial distribution and diffusive motion of rna polymerase in live Escherichia coli. Journal of Bacteriology. 2011;193(19):5138–5146. doi:10.1128/JB.00198-11.

[12] Ramos JL, Mart M, Molina-henares AJ, Tera W, Brennan R, Tobes R. TetR family of transcriptional. 2005;69(2):1–31. doi:10.1128/MMBR.69.2.326.

[13] Kuhlman TE, Cox EC. Gene location and DNA density determine transcription factor distributions in Escherichia coli. Molecular Systems Biology. 2012;8(610):1–13. doi:10.1038/msb.2012.42.

[14] Lederer H, Tovar K, Baer G, May RP, Hillen W, Heumann H. The quaternary structure of Tet repressors bound to the Tn10-encoded tet gene control region determined by neutron solution scattering. The EMBO Journal. 1989;8(4):1257–1263. doi:10.1002/j.1460-2075.1989.tb03499.x.

[15] Pilz I, Goral K, Kratky O, Wade-Jardetzky NG, Jardetzky O, Bray RP. Small-Angle X-Ray Studies of the Quaternary Structure of the Lac Repressor from Escherichia Coli. Biochemistry. 1980;19(17):4087–4090. doi:10.1021/bi00558a028.

[16] Harrington LB, Doxzen KW, Ma E, Liu JJ, Knott GJ, Edraki A, et al. A Broad-Spectrum Inhibitor of CRISPR-Cas9. Cell. 2017;170(6):1224–1233.e15. doi:10.1016/j.cell.2017.07.037.

[17] Atkinson MR, Savageau MA, Myers JT, Ninfa AJ. Development of genetic circuitry exhibiting toggle switch or oscillatory behavior in Escherichia coli. Cell. 2003;113(5):597–607. doi:10.1016/S0092-8674(03)00346-5.

[18] Del Vecchio D. Design and analysis of an activator-repressor clock in E. Coli. In: Proceedings of the American Control Conference; 2007. p. 1589–1594.

[19] Del Vecchio D, Murray RM. Biomolecular Feedback Systems; 2014.

[20] Grossmann C, Roos HG, Stynes M. Numerical Treatment of Partial Differential Equations; 2007.

[21] Strang G. Computational science and engineering. Wellesley-Cambridge Press Wellesley; 2007.

[22] Tabor S. Expression Using the T7 RNA Polymerase/Promoter System. Current Protocols in Molecular Biology. 2004;doi:10.1002/0471142727.mb1602s11.

[23] Knobloch E, Krechetnikov R. Problems on time-varying domains: Formulation, dynamics, and challenges. Acta Applicandae Mathematicae. 2015;137(1):123–157. doi:10.1007/s10440-014-9993-x.

[24] Hayashi K, Morooka N, Yamamoto Y, Fujita K, Isono K, Choi S, et al. Highly accurate genome sequences of Escherichia coli K-12 strains MG1655 and W3110. Molecular Systems Biology. 2006;doi:10.1038/msb4100049.
